# Origins of genome-editing excisases as illuminated by the somatic genome of the ciliate *Blepharisma*

**DOI:** 10.1073/pnas.2213887120

**Published:** 2023-01-20

**Authors:** Minakshi Singh, Brandon K.B. Seah, Christiane Emmerich, Aditi Singh, Christian Woehle, Bruno Huettel, Adam Byerly, Naomi A. Stover, Mayumi Sugiura, Terue Harumoto, Estienne C. Swart

**Affiliations:** ^a^Max Planck Institute for Biology, Tuebingen, 72072 Germany; ^b^Max Planck Genome Center Cologne, Max Planck Institute for Plant Breeding, Cologne, 50829 Germany; ^c^Department of Computer Science and Information Systems, Bradley University, Peoria, IL 61625; ^d^Department of Biology, Bradley University, Peoria, IL 61625; ^e^Department of Chemistry, Biology, and Environmental Sciences, Faculty of Science, Nara Women’s University, Nara, 630-8506 Japan

**Keywords:** natural genome editing, transposase, transposon, PiggyBac, PiggyMac

## Abstract

Massive DNA excision occurs regularly in ciliates, ubiquitous microbial eukaryotes with somatic and germline nuclei in the same cell. Tens of thousands of internally eliminated sequences (IESs) scattered throughout the ciliate germline genome are deleted during the development of the streamlined somatic genome. The genus *Blepharisma* represents one of the two high-level ciliate clades (subphylum Postciliodesmatophora) and, unusually, has dual pathways of somatic nuclear and genome development. This makes it ideal for investigating the functioning and evolution of these processes. Here we report the somatic genome assembly of *Blepharisma stoltei* strain ATCC 30299 (41 Mbp), arranged as numerous telomere-capped minichromosomal isoforms. This genome encodes eight PiggyBac transposase homologs no longer harbored by transposons*.* All appear subject to purifying selection, but just one, the putative IES excisase, has a complete catalytic triad. We hypothesize that PiggyBac homologs were ancestral excisases that enabled the evolution of extensive natural genome editing.

## Significance

Genome editing in ciliates is an extensive, natural process that produces radically restructured somatic genomes. This process is strikingly different between the ciliates investigated, with distinct origins for the proposed DNA excisases. PiggyBac transposase homologs are implicated in DNA deletion in the model ciliates *Paramecium* and *Tetrahymena.* Here we describe the somatic genome of a distant relative, *Blepharisma stoltei*. This genome contains multiple PiggyBac transposase homologs, and its deleted DNA resembles that of *Paramecium*. Phylogenetic analysis suggests ciliate PiggyBac-derived excisases descended from a single domestication event that preceded widespread DNA excision. This work sets the stage for distinguishing expression associated with meiosis and fertilization from that directly associated with genome editing, by comparing *Blepharisma*’s alternative pathways of somatic genome development.

**Fig. 1.**
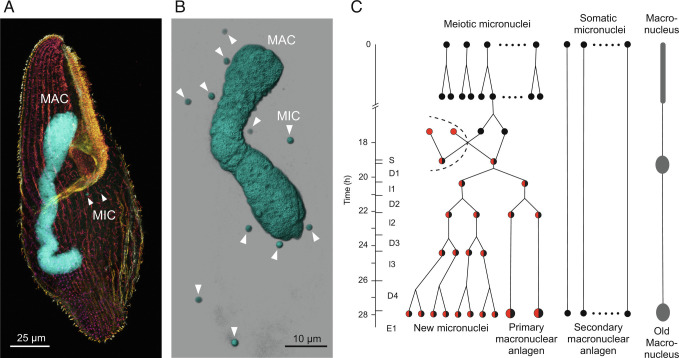
*Blepharisma* nuclei and nuclear development during conjugation. (*A*) Cell of *B. stoltei* strain ATCC 30299 stained with anti-alpha-tubulin-Alexa488 (depth color-coded) and the dsDNA dye DAPI (cyan). (*B*) Snapshot of a 3D reconstruction (Imaris, Bitplane) from CLSM fluorescence images of a cell stained with the dsDNA dye Hoechst 33342 (Invitrogen). (*C*) Schematic of the nuclear processes occurring during conjugation in *Blepharisma*, classified according to, and modified from figure 45 of ref. 6 (copyright, Elsevier). During conjugation, half of the MICs in each cell undergo meiosis (meiotic MICs), and the rest do not (somatic MICs). One of the meiotic MICs eventually gives rise to two haploid gametic nuclei, one of which (the migratory nucleus) is exchanged with that of its partner. Subsequently, the migratory and stationary haploid nuclei fuse to generate a zygotic nucleus (synkaryon), which, after successive mitotic divisions, gives rise to both new MICs and new MACs (known as primary anlagen). The new MACs continue to mature, eventually growing in size and DNA content (6). In parallel, secondary macronuclear anlagen develops directly, and with time, the old MAC condenses and degrades. After karyogamy, cells are classified into ten stages: S (synkaryon), D1 (first mitosis), I1 (first interphase), D2 (second mitosis), I2 (second interphase), D3 (third mitosis), I3 (third interphase), D4 (fourth mitosis), E1 (first embryonic stage), and E2 (second embryonic stage; not shown).

DNA excision in ciliates is a spectacular and widespread form of natural genome editing (1–4). To advance the investigation of such editing and tackle questions about its origins, we focused in this study on the ciliate species *Blepharisma stoltei*. Like all ciliates, cells of *Blepharisma* contain two types of nuclei (Fig. 1 *A* and *B*): larger, somatic macronuclei (MACs) which are transcriptionally active during vegetative growth, and smaller, generally transcriptionally inactive, germline micronuclei (MICs) (5, 6). In model ciliates, such as *Paramecium* and *Tetrahymena,* copies of the genomes of zygotic MICs reorganize to form the new MAC genomes during development (5). In *Blepharisma,* new MACs are also able to develop in this manner (Fig. 1*C*). In this process, internally eliminated sequences (IESs) distributed across the germline genome are systematically removed, and the intervening macronuclear-destined sequences are joined together (5, 7). Unlike in model ciliates, there is an alternative developmental pathway for new MACs in *Blepharisma*, in which they form directly, from distinct MICs called somatoMICs (Fig. 1*C*) (6).

DNA elimination occurs in numerous organisms, frequently removing transposon-derived and other repetitive DNA to form somatic genomes (8). The forms of DNA elimination found in ciliates to date are highly distinctive. For example, the ends of *Paramecium* IESs are terminal inverted repeats that include TA dinucleotides, whereas IESs boundaries in *Oxytricha* are more complex and flanked by direct repeats called pointers (7). IESs can be very short, as in *Paramecium*, with a peak of around 26 to 28 bp (4), or much longer, as in *Tetrahymena*, where they are typically kilobases long (3). IESs are predominantly intergenic in *Tetrahymena* (3), whereas most IESs in *Paramecium* are intragenic and must be removed from DNA to form functional coding sequences (4), analogous to the removal of introns from mRNAs.

Due to some resemblances between ciliate IESs and transposons, such as their terminal inverted repeats, IESs have been hypothesized to originate from transposons (9) and to be excised by domesticated transposases (4, 10–14). In the best-studied ciliate models, genome editing is thought to be coordinated or assisted by small RNAs (sRNAs) (7). Large-scale genome-wide amplification provides the substrates for, and accompanies, genome editing, eventually producing thousands of DNA copies in mature MACs of larger ciliate species (8).

**Fig. 2.**
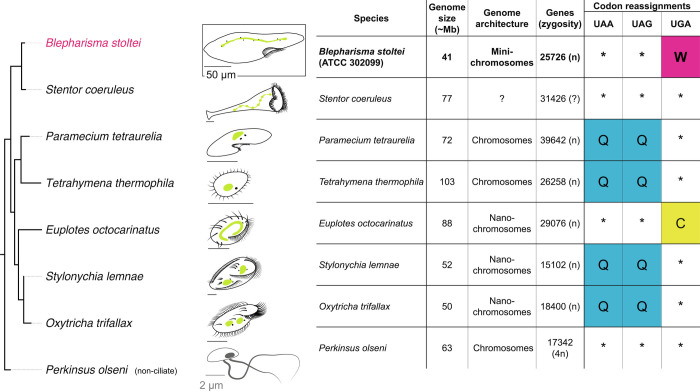
Comparison of basic properties of ciliate MAC genomes. In cell diagrams, MACs are green and MICs are small black dots in close proximity to MACs. Citations for genome properties are in Dataset S1.

Knowledge of the mechanisms of genome editing in ciliates is dominated by *Tetrahymena* and *Paramecium*, with additional input from *Oxytricha*, *Stylonychia* and *Euplotes* (Fig. 2 and *SI Appendix*, Table S1). Investigations of these ciliates have shown that transposases are responsible for IES excision. In *Paramecium* and *Tetrahymena*, the primary IES excisases are domesticated PiggyBac transposases (11, 14). In *Oxytricha*, transposases encoded by MIC genome-specific “telomere-bearing elements” (TBEs), which are autonomous TC1-family transposons, have been hypothesized to be involved in IESexcision (13). The genomes of these ciliates also encode additional transposases, present either as genes of transposons limited to the MIC genomes or as genes apparently not borne by transposons present in the MAC genome (e.g., refs. 2 and 15). Many of these transposases show pronounced upregulation in a developmental time frame similar to that of the presumed IES excisases (2, 15), but await experimental investigation.

Ciliates have been classified into two major subphyla, Postciliodesmatophora and Intramacronucleata (16). Postciliodesmatophora comprises the class Heterotrichea, to which *Blepharisma* belongs, and the class Karyorelictea. Current models are all from subphylum Intramacronucleata: *Tetrahymena* and *Paramecium* belong to class Oligohymenophorea, whereas *Oxytricha*, *Stylonychia,* and *Euplotes* belong to class Spirotrichea (16). Ciliates from Postciliodesmatophora are distantly related to these. Karyorelicts, as their name suggests, were formerly hypothesized to represent the “dawn” of ciliates, with “ancestral” nuclear features (16–18), in contrast to heterotrichs that exhibit “modern” nuclear features and development (16, 19, 20). Though the phylogenetic placement of heterotrichs and karyorelicts no longer supports such hypotheses (21), their nuclear and genomic development are still of much interest.

In recent years, publication of a draft genome for the heterotrich *Stentor coeruleus* has facilitated the revival of this genus for investigations of cellular regeneration (22–24). However, significant hurdles still need to be overcome to investigate genome editing in *Stentor*, as the requisite cell mating has not been observed in the cells corresponding to the reference somatic genome, and very high lethality has been reported for other strains in which mating occurred (25). In contrast, in *Blepharisma*, there is controllable induction of mating (26), and there are established procedures for investigating cellular and nuclear development from more than a century of meticulous cytology, with recent advances made in this effort by Akio Miyake et al. (20, 26–30) (Fig. 1*C*).

Two mating types are known in *Blepharisma*, mediated by pheromone-like molecules called gamones (31, 32). Cells of complementary mating types can form conjugative pairs that undergo sexual reproduction. The strains used in the present study were originally isolated in Germany (strain ATCC 30299) (33) and Japan (strain HT-IV), with the former continuously cultured for over fifty years and the latter for over a decade. They represent two complementary mating types (34): mating type 1 (ATCC 30299) which secretes gamone 1, a 30 kDa glycoprotein (31, 35), and mating type 2 (HT-IV), which secretes gamone 2, a small-molecule derivative of tryptophan (32).

In the conventional development pathway in *Blepharisma*, primary developing new MACs (primary anlagen) mature from zygotic nuclei (Fig. 1*C*). In the alternative pathway, somatoMICs that have not undergone meiosis give rise to secondary anlagen that develop into mature MAC (6). This alternative pathway occurs in strains with a high selfing frequency, where monoclonal cells readily form conjugants among themselves (6), and has also been observed following primary MAC anlagen removal by microsurgery to generate new MACs that eventually mature and replace the old MACs (6). In principle, DNA editing needs to occur in both primary and secondary anlagen to produce functional MAC genomes, since the *B*. *stoltei* MIC genome has numerous gene-interrupting IESs (36).

In this study, we provide the essential somatic genome and developmental-transcriptomic resources for *B. stoltei.* As in model ciliates, MIC-limited sequences are removed to form the functional MAC genome (36). The resulting MAC genome appears to be largely, but not completely, clear of mobile elements and other forms of junk DNA contained in the MIC genome. Among *Blepharisma*’s MAC genome-encoded transposase genes are PiggyBac transposase homologs, some of which are substantially up-regulated during MAC development, including the main candidate IES excisase.

## Results

### A Compact Somatic Genome with Numerous Alternative Telomere Addition Sites (ATASs).

**Fig. 3.**
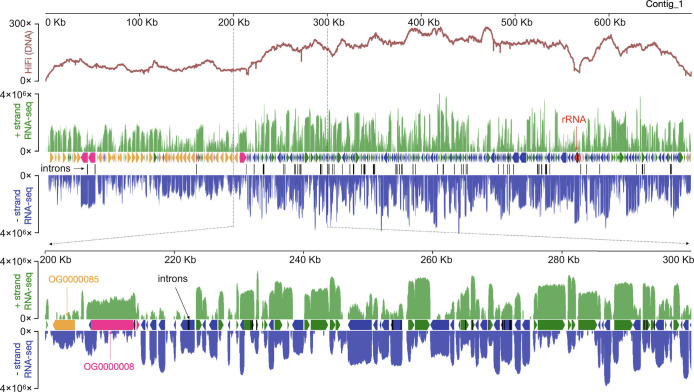
Gene-dense somatic genome. HiFi (DNA) and RNA-seq coverage across a representative *B. stoltei* ATCC 30299 MAC genome contig (Contig_1). Y scale is linear for HiFi reads and logarithmic (base 10) for RNA-seq. Plus strand (relative to the contig) RNA-seq coverage is green; minus strand RNA-seq coverage is blue. Between the RNA-seq coverage graphs, each horizontal arrow represents a predicted gene. Two orthogroups classified by OrthoFinder are shown.

The draft *B. stoltei* ATCC 30299 MAC genome is compact (41 Mbp) and AT rich (66%), like most sequenced ciliate MAC genomes (Fig. 2 and *SI Appendix*, Fig. S1 and Tables S1 and S2). As judged by high BUSCO scores (<2% missing orthologs), it is also relatively complete (*SI Appendix*, Fig. S2*A*). The genome is gene-dense (Fig. 3; 25,711 predicted genes), with short intergenic regions, tiny, predominantly 15 and 16 bp introns (*SI Appendix*, Fig. S3 and *Tiny spliceosomal introns*), and untranslated regions. *B. stoltei* uses an alternative nuclear genetic code with UGA codons reassigned from stops to tryptophan (*SI Appendix*, Fig. S2*B*).

From joint variant calling of reads from strains ATCC 30299 and HT-IV, strain ATCC 30299 appears to be virtually homozygous, with only 1,277 heterozygous single-nucleotide polymorphisms (SNPs) compared to 193,725 in strain HT-IV (i.e., individual heterozygosity of 3.08 × 10^−5^ vs. 4.67 × 10^−3^, respectively). Low SNP levels were likely beneficial for overall genomic contiguity since heterozygosity poses significant algorithmic challenges for assembly software (37). For brevity’s sake, we refer to this genome as the *Blepharisma* MAC genome and “*Blepharisma*” for the associated strain. Though the final assembly comprises 64 telomere-to-telomere sequences (*SI Appendix*, Table S1 and S2 and Fig. S1*A*), it is not possible to define MAC chromosome boundaries given the extensive natural fragmentation of the *Blepharisma* MAC genome (characterized in the next section); hence, we simply refer to “contigs”.

Telomeric reads are distributed across the entire genome (*SI Appendix*, Fig. S4*A*). With a moderately strict definition of possessing at least three consecutive telomeric repeats (each repeat is a permutation of CCCTAACA; SI: “Telomeres in *Blepharisma*”; *SI Appendix*, Fig. S4*D*), one in eight reads in the *Blepharisma* HiFi library were telomere-bearing. In comparison, the telomere-bearing reads of the model ciliate *Tetrahymena thermophila* predominantly map to chromosome ends, with only one in fifty-nine *T. thermophila* CLR reads containing telomeres (at least three consecutive telomeric subunit repeats, 3×CCCCAA). Typically, a minority of mapped reads are telomere-bearing at individual internal positions, so we term them ATASs (*SI Appendix*, Fig. S4*A*). We identified 46,705 potential ATASs, the majority of which (38,686) were represented by only one mapped HiFi read.

The expected distance between telomeres, and hence the average MAC DNA molecule length, is about 130 kbp. This is consistent with the raw input MAC DNA lengths, which were mostly longer than 10 kbp and as long as 1.5 Mbp (*SI Appendix*, Fig. S5 *A* and *B*), and the small fraction (1.3%) of *Blepharisma*’s HiFi reads bound by telomeres on both ends. Excluding the length of the telomeres, telomere-bound reads may be as short as 4 kbp (*SI Appendix*, Fig. S4*C*). Given the frequency of telomere-bearing reads, we expect many additional two-telomere DNA molecules longer than 12 kbp, the maximum length of the HiFi reads excluding telomeres (*SI Appendix*, Fig. S4*B*).

### Key Features of Gene Expression during New MAC Development.

**Fig. 4.**
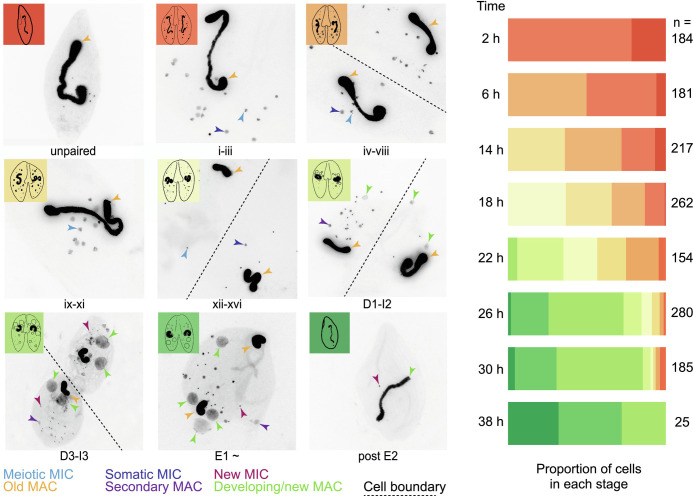
Developmental staging of *B. stoltei* for RNA-seq. Classification of nuclear morphology into stages is according to previous descriptions (6). Nuclear events occurring before and up to, but not including fusion of the gametic nuclei (syngamy) are classified into sixteen stages indicated by roman numerals. These are the pre-gamic stages of conjugation where the MICs undergo meiosis and the haploid products of meiotic MICs are exchanged between the conjugating cells. Stages after syngamy are classified into ten stages as in Fig. 1. Illustration of various cell stages (adapted from ref. 39). Stacked bars show the proportion of cells at each time point at different stages of development, preceded by the number of cells inspected (n).

We obtained an overview of possible molecular processes during *Blepharisma* genome editing from gene expression profiles across development. Complementary *B. stoltei* strains were treated with gamones of the opposite mating type, before mixing to initiate conjugation (6, 38). Samples for morphological staging and RNA-seq were taken at intervals from the time of mixing (“0 h” time point) up to 38 h (Fig. 4 and *SI Appendix*, Fig. S6). During *Blepharisma* conjugation, meiosis begins around 2 h after conjugating cell pairs form and continues up to 18 h, by when gametic nuclei generated by meiosis have been exchanged (Figs. 1*C* and 4). This is followed by karyogamy and mitotic multiplication of the zygotic nucleus (22 h). At 26 h, new, developing primary MACs can be observed in the conjugating pairs as large, irregular bodies (Fig. 4). These nuclei mature into the new MACs of the exconjugant cell by 38 h, after which cell division generates two daughter cells. Smaller secondary MACs, derived directly from MICs without all the intermediate nuclear stages, can also be seen from 22 h, eventually disappearing, and giving way to the primary MACs (Fig. 4).

Examining gene expression at 26 h, when the majority of cells are forming a new MAC (Fig. 4), we observe two broad trends: relatively stable constitutive gene expression (*SI Appendix*, Table S3 and Dataset S3), e.g., an actin homolog (ENA accession: BSTOLATCC_MAC19444) and a bacteria-like globin protein (BSTOLATCC_MAC21846), vs. pronounced development-specific upregulation (*SI Appendix*, Table S4 and Dataset S3), e.g., a histone (BSTOLATCC_MAC21995), an HMG box protein (BSTOLATCC_MAC14030), and a translation initiation factor (eIF4E, BSTOLATCC_MAC5291).

In descending order, ranking the relative gene expression at 26 h vs the average expression of starved, gamone-treated, and 0 h cells revealed numerous genes of interest, including homologs of proteins involved in genome editing in model ciliates (*SI Appendix*, Table S4). Among the top 100 genes (69× to 825× upregulation), nine contain transposase domains from PFAM: DDE_Tnp_1_7, DDE_3, MULE, and DDE_Tnp_IS1595. We also observe sRNA biogenesis and transport proteins, notably a Piwi protein (BSTOLATCC_MAC5406) and a Dicer-like protein (BSTOLATCC_MAC1138; SI “Homologs of sRNA-related proteins involved in ciliate genome editing” and *SI Appendix*, Fig. S7) and a POT1 telomere-binding protein homolog (POT1.4; BSTOLATCC_MAC1496; SI “Telomere-binding protein paralogs” and *SI Appendix*, Fig. S5*C*).

Numerous homologs of genes involved in DNA repair and chromatin are also present among these highly developmentally up-regulated genes (*SI Appendix*, *Development-specific upregulation of proteins associated with DNA repair and chromatin*). The presence of proteins involved in either transcription initiation or translation initiation among these highly up-regulated genes suggests a possible manner in which regulation of development-specific gene expression may be coordinated (*SI Appendix*, *Development-specific upregulation of proteins associated with the initiation of transcription and translation*).

### A Single *Blepharisma* PiggyBac Homolog has a Complete Catalytic Triad.

In *Paramecium tetraurelia* and *T. thermophila*, PiggyBac transposases are responsible for IES excision during genome editing (11, 14). These transposases appear to have been domesticated, no longer being contained in transposons but present in the somatic genome, where they play an essential genome development role (11, 14). PiggyBac homologs typically have a DDD catalytic triad rather than the more common DDE triad of other DDE/D transposases (40). The DDD catalytic motif is present in *Paramecium* PiggyMac (Pgm) and *Tetrahymena* PiggyBac homologs Tpb1 and Tpb2 (10, 11). Among ciliates, domesticated PiggyBac transposases have so far only been reported in these model oligohymenophorean genera. Notably, they have not been detected in either the MAC or MIC genome of the spirotrich *Oxytricha trifallax* (2, 15).

**Fig. 5.**
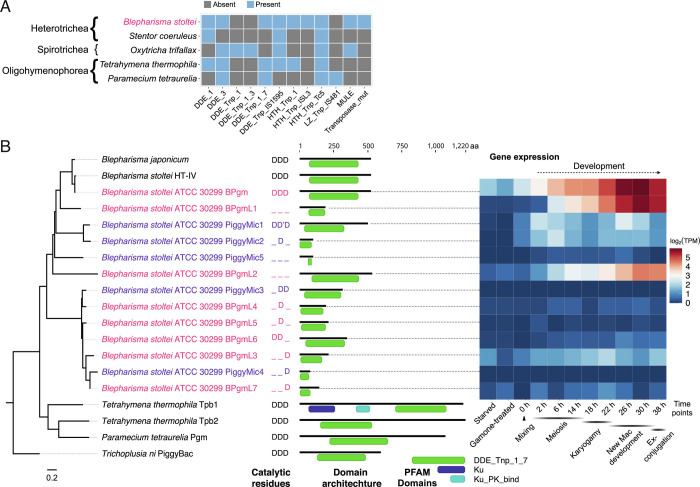
MAC genome-encoded transposases in ciliates and properties of a putative *Blepharisma* IES excisase. (*A*) Presence/absence matrix of PFAM transposase domains detected in predicted MAC genome-encoded ciliate proteins. Ciliate classes are indicated before the binomial species names. (*B*) DDE_Tnp_1_7 domain phylogeny with PFAM domain architecture and gene expression heatmap for *Blepharisma*. “Mixing” indicates when cells of the two complementary mating types were mixed. Outgroup: PiggyBac element from *Trichoplusia ni*. Catalytic residues: D—aspartate, D'—aspartate residue with 1 aa translocation.

We detected more transposase domains (nine distinct PFAM identifiers) in *Blepharisma* than any other ciliate species we examined (Fig. 5*A*). Using HMMER searches with the domain characteristic of PiggyBac homologs, DDE_Tnp_1_7 (PF13843), we found eight homologs in *B. stoltei* ATCC MAC genome and five additional ones within IESs, none of which were flanked by terminal repeats (identified by RepeatModeler). We also found PiggyBac homologs in preliminary assemblies of the MAC genomes of *B. stoltei* HT-IV (ENA: SAMEA9202786) and *Blepharisma japonicum* R1072 (ENA: SAMEA9533699).

Reminiscent of *P. tetraurelia*’s single PiggyMac paralog with a complete catalytic triad among ten paralogs (10), the complete triad is preserved in just a single *Blepharisma* PiggyBac paralog (Fig. 5*B*; Contig_49.g1063, BSTOLATCC_MAC17466). Expression of this gene ramps up from early development, peaking between 22 h and 38 h, when new MACs develop and IES excision is required (Fig. 5*B*). Low levels of expression of this PiggyBac homolog and a few others can be observed even in starved cells. In a multiple sequence alignment, the canonical catalytic triad second aspartate of a lower-expressed, MIC-limited PiggyBac is offset by one amino acid (Datasets S4 and S5).

There are significant similarities in the basic properties of *Blepharisma* and *Paramecium* IESs, detailed in the *Blepharisma* MIC genome report (36). Consequently, adopting the *Paramecium* nomenclature, we refer to the primary candidate IES excisase as *Blepharisma* PiggyMac (BPgm) and the other somatic homologs as BPgm-Likes (BPgmLs). By extension, we refer to their germline-limited counterparts as PiggyMics (Fig. 5*B*).

Other than the PFAM DDE_Tnp_1_7 domain, three *Blepharisma* MAC genome-encoded PiggyBac homologs also possess a short, characteristic cysteine-rich domain (CRD) (*SI Appendix*, Fig. S8), which is absent from the other BPgmLs and PiggyMics. This CRD bears a closer resemblance to the CRD of human PiggyBac-derived protein (PGBD) 4 and the PiggyBac-like element of the fall armyworm moth (*Spodoptera frugiperda*) than that of the PiggyBac homologs of *Paramecium* and *Tetrahymena*.

### *Blepharisma* and *Paramecium* PiggyBac Transposase Homologs Are Subject to Purifying Selection.

Previous experiments involving individual or paired gene knockdowns of most of the ten *P. tetraurelia* PiggyMac(-like) paralogs led to substantial IES retention, even though only one PiggyMac gene (Pgm) has the complete catalytic triad, indicating that all these proteins are functional (10). To examine functional constraints on *Paramecium* PiggyMac homologs, we examined non-synonymous (d_N_) to synonymous substitution rates (d_S_), estimating ω = d_N_/d_S_, for pairwise codon sequence alignments, using two closely related *Paramecium* species (*P*. *tetraurelia* and *P*. *octaurelia*). All d_N_/d_S_ values for pairwise comparisons of each of the catalytically incomplete *P. tetraurelia* PgmLs vs. the complete Pgm were less than 1, ranging from 0.01 to 0.25 (*SI Appendix*, Table S5). All d_N_/d_S_ values for pairwise comparisons between *P. tetraurelia* and *P. octaurelia* PiggyBac orthologs were also substantially less than 1, ranging from 0.02 to 0.11 (*SI Appendix*, Table S6).

Only one of *Blepharisma*’s eight MAC and five MIC PiggyBac homologs has the complete, characteristic DDD triad necessary for catalysis. In pairwise comparisons of each of the MAC homologs with incomplete/missing triads vs. the complete one d_N_/d_S_ ranges from 0.0076 to 0.1351 (*SI Appendix*, Table S7). The pairwise non-synonymous to synonymous substitution rates of the PiggyMics in comparison with the BPgm were also much less than 1 (range 0.007 to 0.2), indicating they are also subject to similar purifying selection. d_N_/d_S_ = 1 indicates genes evolving neutrally (41), which suggests none of the *Blepharisma* PiggyBac homologs genes are likely pseudogenes. However, most of the short BPgmLs and PiggyMics are expressed at low to very low levels, suggestive of pseudogenization (Fig. 5*B*). d_N_/d_S_ estimates of the shorter genes should also be interpreted with caution, since our search procedure required sufficient conservation for homologs to be detected, and because the regions they are calculated across exclude less-conserved flanking ones with insufficient homology.

### PiggyBac Homologs originated Early in Ciliate Evolution.

**Fig. 6.**
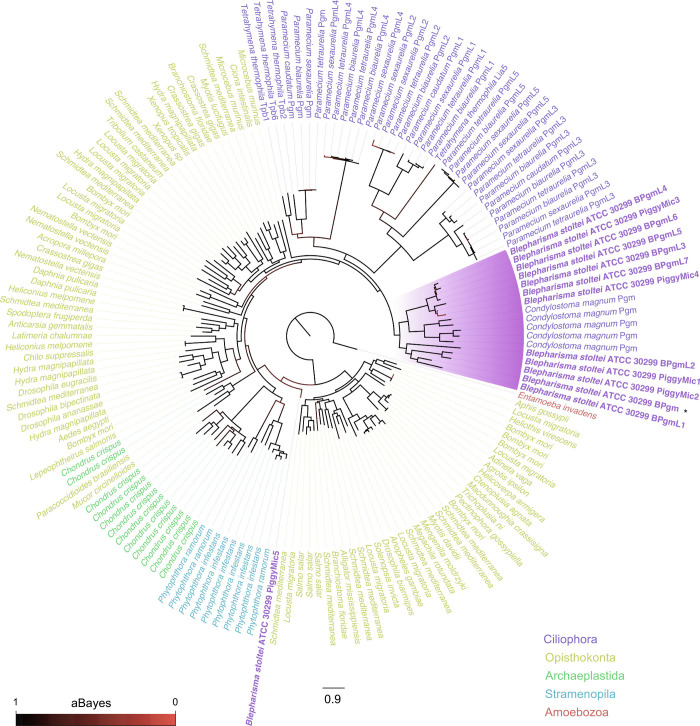
Phylogeny of ciliate PiggyBac homologs and eukaryotic PBLEs. The highlighted clade contains all PiggyBac homologs found in Heterotrichea, containing MAC and MIC-limited homologs of PiggyMac from *Blepharisma* and PiggyMac homologs of *C. magnum.* The tree is rooted at the PiggyBac-like element of *Entamoeba invadens.*

We detected PiggyBac homologs in two other heterotrich species, *S. coeruleus* and *Condylostoma magnum*, but not the oligohymenophorean *Ichthyophthirius multifiliis* (*SI Appendix*, *PiggyBac homologs in other heterotrichs, but not the oligohymenophorean,* Ichthyophthirius multifiliis). To determine whether the *Blepharisma* PiggyBac homologs share a common ciliate ancestor with the oligohymenophorean PiggyBacs, or whether they arose from independent acquisitions in major ciliate groups, we created a large phylogeny of PiggyBac homologs representative of putative domesticated transposases from *B. stoltei* ATCC 30299, *C. magnum*, *Paramecium* spp., *T. thermophila*, as well as PiggyBac-like elements [(PBLEs (42)] from diverse eukaryotes (Fig. 6 and Dataset S1). All the heterotrichous ciliates PiggyBac homologs: BPgm, BPgmLs 1-7, and PiggyMics grouped together with the *Condylostoma* Pgms. The ciliate Pgms and PgmLs largely cluster as a single clade, with the exception of PiggyMic5, which appears as a low-support outgroup to opisthokont, archaeplastid, and stramenopile PiggyBac-like elements. PiggyMic5 had the shortest detected DDE_Tnp_1_7 domain (26 a.a.) and appeared to be poorly aligned relative to the other homologs.

### *Blepharisma*’s MAC Genome Encodes Additional Domesticated Transposases.

As summarized in Fig. 5*A*, *Blepharisma*’s MAC genome encodes a range of additional potential proteins with transposase domains (*SI Appendix*, Blepharisma’s *MAC genome encodes additional domesticated transposases*). All the genes encoding these proteins lack flanking terminal repeats characteristic of active transposons, suggesting they are further classes of domesticated transposases. Many of these are also strongly up-regulated during development and contain complete catalytic triads (present in proteins with DDE_3, DDE_Tnp_IS1595, and MULE transposase domains) (*SI Appendix*, Figs. S9 and S10). In the future, molecular experiments would need to be conducted in *Blepharisma* and other ciliates to characterize the role of these transposase-derived proteins.

In addition to cut-and-paste transposases, we detected a family (>30 copies) of APE-type non-LTR retrotransposon genes encoding proteins with two characteristic domains present on adjacent genes: an APE endonuclease domain (PFAM “exo_endo_phos_2”; PF14529) and a reverse transcriptase domain (PFAM “RVT_1”; PF00078). Unlike the conventional transposase-derived genes in *B. stoltei*, the expression of all these genes throughout the conditions we examined is negligible, and some also appear to be truncated pseudogenes (Dataset S3; workbook “RVT1 + exo_endo_phos_2”). Since it is necessary to understand the relationship of these sequences with respect to IESs, and that they are not due to residual MIC DNA contamination, their analysis is reported in the context of the *B. stoltei* MIC genome (36).

## Discussion

*Blepharisma* species, as representatives of one of the two ciliate subphyla, provide a valuable vantage point from which to view the evolution of nuclear and genomic dimorphism in ciliates, particularly the extensive genomic editing occurring during MAC development. The annotated draft *B. stoltei* ATCC 30299 MAC genome and associated transcriptomic data provide the basis for comparative studies of genome editing.

### The *Blepharisma* MAC Genome has a Minichromosomal Architecture.

Since the lengths of the sequenced two-telomere MAC DNA molecules of *Blepharisma*, on average, imply that they encode tens to a few hundred genes, we propose classifying them as “minichromosomal”. This places them between the “nanochromosomes” of ciliates like *Oxytricha* and *Stylonychia*, which are typically a few kilobases long and encode single genes (15, 43), and *P. tetraurelia* and *T. thermophila* MAC chromosomes which are >50 kbp to megabases long (570 kbp average for *T. thermophila*) (44–46).

The *Paramecium bursaria* MAC genome was shown to be considerably more fragmented than those of other previously examined *Paramecium* species, with greater sequence coverage variation that implies greater DNA copy number variation. This species’ DNA molecules have thus also been classified as minichromosomes (47). We also observed considerable variability in sequence coverage in *Blepharisma* (Fig. 3), suggesting an association between minichromosomes and some DNA copy number variability. The designation of MAC genome architectures as “nanochromosomal”, “minichromosomal”, and “chromosomal” is necessarily rough, with overlap in the size ranges. Nevertheless, it is conceptually useful in considering the nature and spectrum of variability of the underlying DNA.

### *Blepharisma* PiggyMac is the Primary Candidate IES Excisase.

A considerable body of evidence implicates PiggyBac homologs in IES excision of the oligohymenophorean ciliates *Tetrahymena* and *Paramecium* (4, 10–12, 14). The responsible IES excisases in the less-studied spirotrichs *Oxytricha*, *Stylonychia,* and *Euplotes* are not as evident. *Oxytricha*’s TBE transposases are considered to be involved in IES excision but are encoded by full-length germline-limited transposons and are absent from the MAC (13), unlike the primary, MAC genome-encoded IES excisase (Tpb2) in *Tetrahymena* and *Paramecium* PiggyMac and PiggyMac-likes. The pronounced developmental upregulation of numerous additional MAC- and MIC-encoded transposases in *Oxytricha* raises the possibility that transposases other than those of TBEs could also be involved in IES excision (2, 15). Knowledge of IESs in other ciliates is sparse, primarily confined to the phyllopharyngean *Chilodonella uncinata* (48, 49). As far as we are aware, no specific IES excisases have been proposed for this ciliate species.

In current models of IES excision, MIC-limited sequence demarcation by deposition of methylation marks on histones occurs in a sRNA-dependent process (7). These sequences are recognized by domesticated transposases whose excision is supported by additional proteins that somehow recognize these marks (7). Together with MIC sequencing, we observed abundant development-specific sRNA production in *Blepharisma* resembling other model ciliates (36). Homologs of proteins implicated in ciliate genome editing were present among the genes most highly differentially up-regulated during new MAC development, notably including Dicer-like and Piwi proteins, which are candidate genes responsible for development-specific sRNA biogenesis and transport (*SI Appendix*, Fig. S7).

Since the oligohymenophorean PiggyBac homologs are clear IES excisases, we sought and found eight homologs of these genes in the *Blepharisma* MAC genome and five in the MIC genome. *Blepharisma* is the first ciliate genus aside from *Tetrahymena* and *Paramecium* in which such proteins have been reported and distantly related to both. Additional searches revealed clear PiggyBac homologs in *C. magnum* (Fig. 6) and a weaker pair of matches in *S. coeruleus*, suggesting that these are a common feature of heterotrich ciliates. Reminiscent of *P. tetraurelia*, in which just one of the nine PiggyBac homologs, PiggyMac, has a complete DDD catalytic triad (10), a single *Blepharisma* PiggyBac homolog has a complete canonical DDD catalytic triad. As is characteristic of PiggyBac homologs, each of these three proteins also has a C-terminal, cysteine-rich, zinc finger domain. The organization of the heterotrich PiggyBac homolog zinc finger domains is more similar to comparable domains of *Homo sapiens* PGBD2 and PGBD3 homologs than the zinc finger domains in *Paramecium* and *Tetrahymena* PiggyBac homologs.

Like *Paramecium* PiggyMac, *Blepharisma* PiggyMac is also strongly up-regulated in development during new MAC formation. *Paramecium* PiggyMac expression begins very early in development, during meiosis, and this protein does not appear to localize to old MAC (14). If there were some BPgm expression prior to primary anlagen formation, and the proteins similarly did not localize in the old MAC, this would likely be inconsequential. On the other hand, it is also possible that some BPgm is transported to and gets used earlier during development of the secondary anlagen than in the primary ones. In the future, it will be necessary to investigate this experimentally.

Since the discovery of multiple PiggyBac homologs (PiggyMac-likes) in *Paramecium,* there have been questions about their role. Aside from PiggyMac, all PiggyMac-likes have incomplete catalytic triads and are thus likely catalytically inactive, but their gene knockdowns nevertheless lead to pronounced IES retention (10). It has therefore been proposed that the PiggyMac-likes may support PiggyMac during DNA excision, co-assembling in multi-subunit complexes (10). On the other hand, cryo-EM structures available for moth PiggyBac transposase support a model in which these proteins function as a homodimeric complex in vitro (50). Furthermore, the primary *Tetrahymena* PiggyBac, Tpb2, is reported to perform cleavage in vitro alone (11). In other eukaryotes, domesticated PiggyBacs without complete catalytic triads are thought to be retained due to co-option of their DNA-binding domains (51). One possibility for such purely DNA-binding transposase-derived proteins in ciliates could be in competitively regulating (taming) the excision of DNA by the catalytically active transposases. Future experimental analyses of the BPgm and the BPgm-likes could aid in understanding possible interactions between catalytically active and inactive transposases.

### *Blepharisma* has Additional Domesticated Transposases whose Roles await Determination.

All ciliate species have additional MAC genome-encoded transposase families other than those proposed to be involved in IES excision (Fig. 5*A*). Though upregulation of some of these homologs in model ciliates has been noted (2, 15, 52), their roles remain to be determined. In addition to the PiggyBac homologs, we found potentially domesticated MAC genome-encoded transposases with the PFAM domains “DDE_1”, “DDE_3”, “DDE_Tnp_IS1595”, and “MULE” in *Blepharisma* (*SI Appendix*, Blepharisma’s *MAC genome encodes additional domesticated transposases*).

Aside from the timing of IES excisase expression, coinciding with new MAC genome formation, the manner in which the excisases perform excision is also crucial. Upon excision, classical cut-and-paste transposases in eukaryotes typically leave behind additional bases, notably including the target-site duplication arising when they were inserted, forming a “footprint” (53). PiggyBac homologs are unique in performing precise, “seamless” excision in eukaryotes (54), conserving the number of bases at the site of transposon insertion after excision, a property that makes them popular for genetic engineering (50). *Tetrahymena* Tpb2 is the one exception among PiggyBac homologs associated with imprecise excision (11). Since intragenic IESs are abundant in *Blepharisma*, like *Paramecium* and unlike *Tetrahymena*, it is essential that these are excised precisely.

Though there are clearly numerous additional domesticated transposases with complete catalytic triads and whose genes are substantially up-regulated during *Blepharisma* development, the extent to which they are capable of precise excision needs to be established. *Tetrahymena* has distinct domesticated transposases that excise different subsets of IESs, namely those that are predominant, imprecisely excised and intergenic (by Tpb2) (11), vs. those that are rare, precisely excised and intragenic (by Tpb1 and Tpb6) (12, 55). Consequently, if the additional *Blepharisma* domesticated transposases are still capable of excision, but not a precise form, we could envisage an involvement in excision of a subset of the numerous intergenic IESs.

Just as it is not possible to preclude the involvement of additional transposases other than PiggyBac homologs in IES excision in *Blepharisma*, it is not possible to preclude the existence and involvement of additional IES excisases in the ciliate common ancestor. As transposons are vehicles of horizontal gene transfer (HGT), it will be necessary to scrutinize the phylogenies of all the ciliate transposases once a broader representation of sequenced ciliate genomes becomes available. Furthermore, such trees should be considered in relation to genes less likely to be subject to HGT, but involved in genome editing, particularly those associated with development-specific sRNAs.

### A Single Origin of PiggyBac Homologs within Ciliates is the most Parsimonious Scenario.

Phylogenetic analyses indicate *Tetrahymena*, *Paramecium,* and *Blepharisma* PiggyBac homologs form a monophyletic clade. However, the lack of PiggyBac homologs in some ciliate classes (and potentially the oligohymenophorean *Ichthyophthirius multifiliis*) raises the question whether PiggyBac IES excisases were lost or replaced in these lineages, or rather gained independently from the same source by heterotrichs and a subset of oligohymenophoreans. We think the former is more likely and consistent with a long-standing hypothesis that favors ancestral IES excisase substitution in particular ciliate lineages (9). However, the alternative cannot be dismissed, because non-model ciliates with sufficient genome assembly quality and reliable gene and domain annotations have only been sparsely sampled.

### Future Directions.

This research pays tribute to the memory of Akio Miyake and his decades of inspirational *Blepharisma* research. The *B. stoltei* ATCC 30299 MAC genome and the corresponding MIC genome (36) pave the way for investigations of a unique, direct pathway to new MAC genome development he revealed (6). This pathway skips most of the upstream complexity of the standard pathway in other ciliates (6). The pair of *B. stoltei* strains used are both now strains in which intraclonal conjugating pairs form infrequently (low-frequency selfers). In these cells, the conventional MAC development pathway dominates. In the future, high-frequency *Blepharisma* selfers, in which the direct MAC development pathway dominates, will need to be collected from the wild. With such cells, gene expression between the two pathways can be compared. This will enable expression upregulation due to meiosis and fertilization-specific processes to be distinguished from that of genes directly involved in genome editing.

## Materials and Methods

Materials and Methods are given in full in *SI Appendix*. Briefly, high molecular weight DNA was isolated from the subcellular fractions of enriched MAC and MIC of *B. stoltei* strain ATCC 30299, separated using sucrose-gradient centrifugation (56). The MAC-enriched fraction was sequenced using PacBio HiFi reads and the MAC genome assembled with Flye (version 2.7-b1585) (19). The MIC-enriched fraction was sequenced with PacBio Continuous Long Reads (CLR) and analyzed using the BleTIES pipeline (57) to identify MIC-limited genomic regions.

MAC and MIC-limited genes were predicted with “Intronarrator” (https://github.com/Swart-lab/Intronarrator) and functionally annotated using HMMER3 (hmmscan) (58), Pannzer2 (59), and eggNOG (60). Repeat elements in the MAC and MIC-limited genomes were predicted using RepeatModeler v2.0.1 (61) and classified using RepeatClassifier v2.0.1.

Gene expression at during conjugation and nuclear development was determined by complementing gene annotations with RNA-seq data gathered at different time points during synchronized conjugation between the two *B. stoltei* mating type strains ATCC 30299 (mating type 1) and HT-IV (mating type 2). Conjugation between the two mating types was synchronized by pre-treating both mating types with complementary gamones before mixing the cells (38). Upon mixing the two gamone-treated mating types, the cells form mating pairs. The paired cells were maintained up to 38 h after mixing, and samples for imaging and RNA extraction were taken immediately after mixing the mating types (0 h) and subsequently at 2, 6, 14, 18, 22, 26, 30, and 38 h after mixing. For each time point, cells were counted and classified according to their stage of nuclear development through imaging. RNA extracted at the different time points was used for RNA-seq.

Genes annotated with the PFAM DDE_Tnp_1_7 domain were identified as homologs of PiggyBac in the MAC genome assembly and the MIC-limited genomic regions. dN/dS analyses were performed using PAML (62). For phylogenetic analysis, protein sequences of the regions adjacent to and containing the PFAM DDE_Tnp_1_7 domain of *Blepharisma* PiggyBac homologs in the MAC and MIC-limited genomes together with PiggyBac-like elements from other eukaryotic lineages and domesticated PiggyBac homologs from other ciliates were aligned using MAFFT (63) and used to generate the phylogenetic tree using FastTree2 (64), using the Geneious bioinformatic software (65) plug-ins for both tools.

## Supplementary Material

Appendix 01 (PDF)

## Data Availability

The draft *B. stoltei* ATCC 30299 MAC genome assembly is accessible from https://bleph.ciliate.org/ and from the European Nucleotide Archive (ENA) Bioproject PRJEB40285 (66) under the accession GCA_905310155. PacBio CCS reads (ERR5873783 and ERR5873334) and subreads (ERR5962314) used to assemble the genome are also available from ENA. Illumina DNA-seq data for the *B. stoltei* ATCC 30299 and HT-IV strains are available from accessions ERR6061285 and ERR6064674, respectively. The RNA-seq developmental time course is available from the bioproject PRJEB45374 (67) (accessions ERR6049461 to ERR6049485). Illumina and PacBio Sequel sequencing data for *B. japonicum* strain R1702 are available from the ENA Bioproject PRJEB46921 (68) (Illumina accessions: ERR6473251, ERR6474356; PacBio accession: ERR6474383). Code availability for software we generated or modified is indicated in place in *SI Appendix*. Supplemental data are available from EDMOND (69): https://doi.org/10.17617/3.8c.
