# Supplementary material for: Origins of genome-editing excisases as illuminated by the somatic genome of the ciliate Blepharisma
Source: Proc Natl Acad Sci U S A. 2023 Jan 20;120(4):e2213887120. doi: 10.1073/pnas.2213887120 (PMC9942806; doi:10.1073/pnas.2213887120)
Supplement: Supplementary file 1 — Appendix 01 (PDF) [file pnas.2213887120.sapp.pdf]

Supporting Information for

Origins of genome-editing excisases as illuminated by the somatic genome of the ciliate *Blepharisma*

Minakshi Singh, Kwee Boon Brandon Seah, Christiane Emmerich, Aditi Singh, Christian Woehle, Bruno Huettel, Adam Byerly, Naomi Alexandra Stover, Mayumi Sugiura, Terue Harumoto, Estienne Carl Swart*

* Correspondence: Estienne Carl Swart
Email: estienne.swart@tuebingen.mpg.de

This PDF file includes:

Supporting text
Figures S1 to S11
Tables S1 to S10
SI References

Supporting Information Text

SI Materials and Methods

Strains and localities

The strains used and their original isolation localities were: *Blepharisma stoltei* ATCC 30299, Lake Federsee, Germany (1); *Blepharisma stoltei* HT-IV, Aichi prefecture, Japan; *Blepharisma japonicum* R1072, from an isolate from Bangalore, India (2).

Cell cultivation, harvesting and cleanup

For genomic DNA isolation *B. stoltei* ATCC 30299 and HT-IV cells were cultured in Synthetic Medium for *Blepharisma* (SMB) (3) at 27˚C. Belpharismas were fed *Chlorogonium elongatum* grown in Tris-acetate phosphate (TAP) medium (4) at room temperature. *Chlorogonium* cells were pelleted at 1500 g at room temperature for 3 minutes to remove most of the TAP medium, and resuspended in 50 mL SMB. 50 ml of dense *Chlorogonium* was used to feed 1 litre of *Blepharisma* culture once every three days.

Blepharisma stoltei ATCC 30299 and HT-IV cells used for RNA extraction were cultured in Lettuce medium inoculated with *Enterobacter aerogenes* and maintained at 25˚C (5).

Blepharisma cultures were concentrated by centrifugation in pear-shaped flasks at 100 g for 2 minutes using a Hettich Rotanta 460 centrifuge with swing out buckets. Pelleted cells were washed with SMB and centrifuged again at 100 g for 2 minutes. The washed pellet was then transferred to a cylindrical tube capped with a 100 µm-pore nylon membrane at the base and immersed in SMB to filter residual algal debris from the washed cells. The cells were allowed to diffuse through the membrane overnight into the surrounding medium. The next day, the cylinder with the membrane was carefully removed while attempting to minimize dislodging any debris collected on the membrane. Cell density after harvesting was determined by cell counting under the microscope.

DNA isolation, library preparation and sequencing

B. stoltei macronuclei were isolated by sucrose gradient centrifugation (6). DNA was isolated with a Qiagen 20/G genomic-tip kit according to the manufacturer's instructions. Purified DNA from the isolated MACs was fragmented, size selected and used to prepare libraries according to standard PacBio HiFi SMRTbell protocols. The libraries were sequenced in circular consensus mode to generate HiFi reads.

Total genomic DNA from *B. stoltei* HT-IV and *B. stoltei* ATCC 30299 was isolated with the SigmaAldrich GenElute Mammalian genomic DNA kit. A sequencing library was prepared with a NEBnext FS DNA Library Prep Kit for Illumina and sequenced on an Illumina HiSeq 3000 sequencer, generating 150 bp paired-end reads.

Total genomic DNA from *B. japonicum* was isolated with the Qiagen MagAttract HMW DNA kit. A long-read PacBio sequencing library was prepared using the SMRTbell Express Template Preparation Kit 2.0 according to the manufacturers' instructions and sequenced on an PacBio Sequel platform with 1 SMRT cell. Independently, total genomic DNA form *B. japonicum* was isolated with the SigmaAldrich GenElute Mammalian genomic DNA kit and a sequencing library was prepared with the TruSeq Nano DNA Library Prep Kit (Illumina) and sequenced on an Illumina NovaSeq6000 to generate 150 bp paired-end reads.

Gamone 1/ Cell-Free Fluid (CFF) isolation and conjugation activity assay

Blepharisma is one of only two ciliate genera, along with *Euplotes* (7–10), where conjugation has been shown to be mediated through pheromone-like substances called gamones. *Blepharisma* has two mating types, distinguished by their gamone production. Mating type I cells release gamone 1, a ~30 kDa glycoprotein (11, 12); mating type II cells release gamone 2, calcium-3-(2'-

formylamino-5’-hydroxybenzoyl) lactate, a small-molecule effector (13). *Blepharisma* cells commit to conjugation when complementary mating types recognize each other's gamones, with the cells remaining paired while meiosis and then fertilization occur, and eventually new MACs begin to form.

B. stoltei ATCC 30299 cells were cultured and harvested and concentrated to a density of 2000 cells/mL according to the procedure described in “Cell cultivation, Harvesting and Cleanup”. This concentrated cell culture was incubated overnight at 27˚C. The next day, the cells were harvested, and the supernatant collected and preserved at 4˚C at all times after extraction. The supernatant was then filtered through a 0.22 µm-pore filter. BSA (10 mg/mL) was added to produce the final CFF at a final BSA concentration of 0.01%.

To assess the activity of the CFF, serial dilutions of the CFF were made to obtain the gamone activity in terms of units (U) (14). The activity of the isolated CFF was 2^{10} U.

Conjugation time course and RNA isolation for high-throughput sequencing

B. stoltei cells for the complementary strains, ATCC 30299 and HT-IV, were cultivated and harvested by gentle centrifugation to achieve a final cell concentration of 2,000 cells/ml for each strain. Non-gamone treated ATCC 30299 (A1) and HT-IV cells (H1) were collected (time point: -3 hours). Strain ATCC 30299 cells were then treated with synthetic gamone 2 (final concentration 1.5 µg/mL) and strain HT-IV cells were treated with cell-free fluid with a gamone 1 activity of ~2^{10} U/ml for three hours (Figure S6).

Homotypic pair formation in both cultures was checked after three hours. More than 75% of the cells in both cultures formed homotypic pairs. At this point the samples A2 (ATCC 30299) and H2 (HT-IV) were independently isolated for RNA extraction as gamone-treated control cells just before mixing. For the rest of the culture, homotypic pairs in both cultures were separated by pipetting them gently with a wide-bore pipette tip. Once all pairs had been separated, the two cultures were mixed together. This constitutes the experiment’s 0-h time point. The conjugating culture was observed and samples collected for RNA isolation or cell fixation at 2 h, 6 h, 14 h, 18 h, 22 h, 26 h, 30 h and 38 h. Further details of the sample staging approach are described in (15) and (16). For each sample 7 mL of culture was harvested for RNA-extraction using Trizol. The total RNA obtained was then separated into a small RNA fraction < 200 nt and a fraction with RNA fragments > 200 nt using the Zymo RNA Clean and Concentrator-5 kit according to the manufacturer's instructions. RNA-seq libraries were prepared by BGI according to their standard protocols and sequenced on a BGISeq 500 instrument.

Separate 2 mL aliquots of cells at each time point for which RNA was extracted were concentrated by centrifuging gently at 100 rcf. 50 µL of the concentrated cells were fixed with Carnoy’s fixative (ethanol:acetic acid, 6:1), stained with DAPI and imaged to determine the state of nuclear development (15).

Cell fixation and imaging

B. stoltei cells were harvested as above (“Cell cultivation, harvesting and cleanup”), and fixed with an equal volume of “ZFAE” fixative (17), containing zinc sulfate (0.25 M, Sigma Aldrich), formalin, glacial acetic acid and ethanol (Carl Roth), freshly prepared by mixing in a ratio of 10:2:2:5. Fixed cells were pelleted (1000 g; 1 min), resuspended in 1% TritonX-100 in PHEM buffer to permeabilize (5 min; room temperature), pelleted and resuspended in 2% (w/v) formaldehyde in PHEM buffer to fix further (10 min; room temp.), then pelleted and washed twice with 3% (w/v) BSA in TBSTEM buffer (~10 min; room temp.). For indirect immunofluorescence, washed cells were incubated with primary antibody rat anti-alpha tubulin (Abcam, ab6161; 1:100 dilution in 3% w/v BSA/TBSTEM; 60 min; room temp.) then secondary antibody goat anti-rat IgG H&L labeled with AlexaFluor 488 (Abcam, ab150157, 1:500 dilution in 3% w/v BSA/TBSTEM; 20 min; room temp.). Nuclei were counterstained with DAPI (1 µg/mL) in 3% (w/v) BSA/TBSTEM. A z-stack of images was acquired using a confocal laser scanning microscope (Leica TCS SP8), equipped

with a HC PL APO 40× 1.30 Oil CS2 objective and a 1 photomultiplier tube and 3 HyD detectors, for DAPI (405 nm excitation, 420-470 nm emission) and Alexa Fluor 488 (488 nm excitation, 510-530 nm emission). Scanning was performed in sequential exposure mode. Spatial sampling was achieved according to Nyquist criteria. ImageJ (Fiji) (18) was used to adjust image contrast and brightness and overlay the DAPI and AlexaFluor 488 channels. The z-stack was temporally color-coded.

For a nuclear 3D reconstruction (Fig. 1B), cells were fixed in 1% (w/v) formaldehyde and 0.25% (w/v) glutaraldehyde. Nuclei were stained with Hoechst 33342 (Invitrogen) (5 µM in the culture media), and imaged with a confocal laser scanning microscope (Zeiss, LSM780) equipped with an LD C-Apochromat 40x/1,1 W Korr objective and a 32 channel GaAsP array detector, with 405 nm excitation and 420-470 nm emission. Spatial sampling was achieved according to Nyquist criteria. The IMARIS (Bitplane) software v8.0.2 was used for three-dimensional reconstructions and contrast adjustments.

Genome assembly

Two MAC genome assemblies for *B. stoltei* ATCC 30299 (70× and 76× coverage) were produced with Flye (version 2.7-b1585) (19) for the two separate PacBio Sequel II libraries (independent replicates) using default parameters and the switches: --pacbio-hifi -g 45m. The approximate genome assembly size was chosen based on preliminary Illumina genome assemblies of approximately 40 Mb. Additional assemblies using the combined coverage (145×) of the two libraries were produced using either Flye version 2.7-b1585 or 2.8.1-b1676, and the same parameters. Two rounds of extension and merging were then used, first comparing the 70× and 76× assemblies to each other, then comparing the 145× assembly to the former merged assembly. Assembly graphs were all relatively simple, with few tangles to be resolved (Fig. S1A). Minimap2 (20) was used for pairwise comparison of the assemblies using the parameters: -x asm5 --frag=yes --secondary=no, and the resultant aligned sequences were visually inspected and manually merged or extended where possible using Geneious (version 2020.1.2) (21).

Visual inspection of read mapping to the combined assembly was then used to trim off contig ends where there was little correspondence between the assembly consensus and the mapped reads, which we classify as "cruft". Read mapping to cruft regions was often lower or uneven, suggestive of repeats. Alternatively, these features could be due to trace MIC sequences, or sites of alternative chromosome breakage during development which lead to sequences that are neither purely MAC nor MIC. A few contigs with similar dubious mapping of reads at internal locations, which were also clear sites of chromosome fragmentation (evident by abundant telomere-bearing reads in the vicinity) were split apart and trimmed back as for the contig ends. Telomere-bearing reads mapped to the non-trimmed region nearest to the trimmed site were then used to define contig ends, adding representative telomeric repeats from one of the underlying sequences mapped to each of the ends. The main genome assembly with gene predictions can be obtained from the European Nucleotide Archive (ENA) (PRJEB40285; accession GCA_905310155). "Cruft" sequences are also available from the same accession.

Two separate assemblies were generated for *Blepharisma japonicum.* A genome assembly for *Blepharisma japonicum* strain R1072 was generated from Illumina reads, using SPAdes genome assembler (v3.14.0) (22). An assembly with PacBio Sequel long reads was produced with Ra (v0.2.1) (23), which uses the Overlap-Layout-Consensus paradigm. The assembly produced with Ra was more contiguous, with 268 contigs, in comparison to 1,510 contigs in the SPAdes assembly, and was chosen as the reference assembly for *Blepharisma japonicum* (ENA accession: ERR6474383).

Condylostoma magnum genomic reads (study accession PRJEB9019) from a previous study (24) were reassembled to improve contiguity and remove bacterial contamination. Reads were trimmed with bbduk.sh from the BBmap package v38.22 (https://sourceforge.net/projects/bbmap/), using minimum PHRED quality score 2 (both ends) and k-mer trimming for Illumina adapters and Phi-X phage sequence (right end), retaining only reads

≥25 bp. Trimmed reads were error-corrected and reassembled with SPAdes v3.13.0 (22) using k-mer values 21, 33, 55, 77, 99. To identify potential contaminants, the unassembled reads were screened with phyloFlash v3.3b1 (25) against SILVA v132 (26); the coding density under the standard genetic code and prokaryotic gene model were also estimated using Prodigal v2.6.3 (27). Plotting the coverage vs. GC% of the initial assembly showed that most of the likely bacterial contigs (high prokaryotic coding density, lower coverage, presence of bacterial SSU rRNA sequences) had >=40% GC, so we retained only contigs with <40% GC as the final *C. magnum* genome bin. The final assembly is available from the ENA bioproject PRJEB48875 (accession GCA_920105805).

All assemblies were inspected with the quality assessment tool QUAST (28).

Variant calling

Illumina total genomic DNA-seq libraries for *B. stoltei* strains ATCC 30299 (ENA accession: ERR6061285) and HT-IV (ERR6064674) were mapped to the ATCC 30299 reference assembly with bowtie2 v2.4.2 (29). Alignments were tagged with the MC tag (CIGAR string for mate/next segment) using samtools (30) fixmate. The BAM file was sorted and indexed, read groups were added with bamaddrg (commit 9baba65, https://github.com/ekg/bamaddrg), and duplicate reads were removed with Picard MarkDuplicates v2.25.1 (http://broadinstitute.github.io/picard/). Variants were called from the combined BAM file with freebayes v1.3.2 (31) in diploid mode, with maximum coverage 1000 (option -g). The resultant VCF file was combined and indexed with bcftools v1.12 (30), then filtered to retain only SNPs with quality score > 20, and at least one alternate allele.

Comparison of telomere-bearing read fraction of *Blepharisma* and *Tetrahymena*

A simple regular expression search for three successive telomeric subunit repeats ("3xCCCTAACA" for *Blepharisma*, "3xCCCCAA" for *Tetrahymena*) was used to extract and estimate the proportion of telomere-bearing reads. Visual inspections of these reads mapped with minimap2 to the respective *B. stoltei* ATCC 30299 MAC genome assembly and *T. thermophila* MAC genome assembly (32) suggested that most (> 90%) reads naively classified this way were correct. For *B. stoltei* ATCC 30299 we obtained two estimates for the two HiFi read libraries used (12.4% and 11.8% of all reads). For *T. thermophila* we combined all the deposited PacBio subreads used to generate the most recent MAC genome assembly (32), obtaining an estimate of 1.7% of all reads being telomere-bearing.

Annotation of alternative telomere addition sites

Alternative telomere addition sites (ATASs) were annotated by mapping PacBio HiFi reads to the curated reference MAC assembly described above, using minimap2 and the following flags: -x asm20 --secondary=no --MD. We expect reads representing alternative telomere additions to have one portion mapping to the assembly (excluding telomeric regions), with the other portion containing telomeric repeats being soft-clipped in the BAM record. For each mapped read with a soft-clipped segment, we extracted the clipped sequence, and the coordinates and orientation of the clip relative to the reference. We searched for ≥ 24 bp tandem direct repeats of the telomere unit (i.e., ≥3 repeats of the 8 bp unit) in the clipped segment with NCRF v1.01.02 (33), which can detect tandem repeats in the presence of noise, e.g., from sequencing error. The orientation of the telomere sequence, the distance from the end of the telomeric repeat to the clip junction ('gap'), and the number of telomere-bearing reads vs. total mapped reads at each junction were also recorded. Junctions with zero gap between telomere repeat and clip junction were annotated as ATASs. The above procedure was implemented in the MILTEL module of the software package BleTIES v0.1.3 (34).

MILTEL output was processed with Python scripts depending on Biopython (35), pybedtools (36), Bedtools (37), and Matplotlib (38), to summarize statistics of junction sequences and telomere permutations at ATAS junctions, and to extract genomic sequences flanking ATASs for sequence

logos. Logos were drawn with Weblogo v3.7.5 (39), with sequences oriented such that the telomere would be added on the 5' end of the ATAS junctions.

To calculate the expected minichromosome length, we assumed that ATASs were independent and identically distributed in the genome following a Poisson distribution. About 47×10^3 ATASs were annotated, supported on average by a single read. Given a genome of 42 Mbp at 145× coverage, the expected rate of encountering an ATAS is 47×10^3 / (145 × 42 Mbp), so the distance between ATASs (i.e., the minichromosome length) is exponentially distributed with expectation (145 × 42 Mbp) / 47×10^3 = 130 kbp.

RNA-seq read mapping

To permit correct mapping of tiny introns, RNA-seq data was mapped to the *B. stoltei* ATCC 30299 MAC genome using a version of HISAT2 (40) with modified source code, with the static variable minIntronLen in hisat2.cpp lowered to 9 from 20 (change available in the HISAT2 github fork: https://github.com/Swart-lab/hisat2/; commit hash 86527b9). HISAT2 was run with default parameters and parameters --min-intronlen 9 --max-intronlen 500. It should be noted that RNA-seq from timepoints in which *B. stoltei* ATCC 30299 and *B. stoltei* HT-IV cells were mixed together were only mapped to the former genome assembly, and so reads for up to three alleles may map to each of the genes in this assembly.

Genetic code prediction

We used the program PORC (Prediction Of Reassigned Codons; available from https://github.com/Swart-lab/PORC), previously written to predict genetic codes in protist transcriptomes (24), to predict the *B. stoltei* genetic code. This program was used to translate the draft *B. stoltei* ATCC 30299 genome assembly in all six frames (with the standard genetic code). Like the program FACIL (41) that inspired PORC, the frequencies of amino acids in PFAM (version 34.0) protein domain profiles aligned to the six-frame translation by HMMER 3.1b2 (42) (default search parameters; domains used for prediction with conditional E-values < 1e-20), and correspondingly also to the underlying codon, were used to infer the most likely amino acid encoded by each codon (Fig. S2B).

Gene prediction

We created a wrapper program, Intronarrator, to predict genes in *Blepharisma* and other heterotrichs, accommodating their tiny introns. Intronarrator can be downloaded and installed together with dependencies via Conda from GitHub (https://github.com/Swart-lab/Intronarrator). Intronarrator directly infers introns from spliced RNA-seq reads mapped by HISAT2 from the entire developmental time course we generated. RNA-seq reads densely cover almost the entire *Blepharisma* MAC genome, aside from intergenic regions, and most potential protein-coding genes (Fig. 4B). After predicting the introns and removing them to create an intron-minus genome, Intronarrator runs AUGUSTUS (version 3.3.3) using its intronless model. It then adds back the introns to the intronless gene predictions to produce the final gene predictions.

Introns are inferred from "CIGAR" string annotations in mapped RNA-seq BAM files, using the regular expression "[0-9]+M([0-9][0-9])N[0-9]+M" to select spliced reads. For intron inference we only used primary alignments with: MAPQ >= 10; just a single "N", indicating one potential intron, per read; and at least 6 mapped bases flanking both the 5' and 3' intron boundaries (to limit spurious chance matches of a few bases that might otherwise lead to incorrect intron prediction). The most important parameters for Intronarrator are a cut-off of 0.2 for the fraction of spliced reads covering a potential intron, and a minimum of 10 or more spliced reads to call an intron. The splicing fraction cut-off was chosen based on the overall distribution of splicing (Figs. S3A-C). From our visual examination of mapped RNA-seq reads and gene predictions, values less than this were typically "cryptic" excision events (43) which remove potentially essential protein-coding sequence regions, rather than genuine introns. Intronarrator classifies an intron as sense (7389 in total, excluding alternative splicing), when the majority of reads (irrespective of splicing)

mapping to the intron are the same strand, and antisense (554 in total) when they are not. The most frequently spliced intron was chosen in rare cases of overlapping alternative intron splicing.

To eliminate spurious prediction of protein-coding genes overlapping ncRNA genes, we also incorporated ncRNA prediction in Intronarrator. Infernal (44) (default parameters; e-value < 1e-6) was used to predict a restricted set of conserved ncRNAs models (i.e., tRNAs, rRNAs, SRP, and spliceosomal RNAs) from RFAM 14.0 (45). These ncRNAs were hard-masked (with "N" characters) before AUGUSTUS gene prediction. Both Infernal ncRNA predictions (excluding tRNAs) and tRNA-scan SE 2.0 (46) (default parameters) tRNA predictions are annotated in the *B. stoltei* ATCC 30299 assembly deposited in the European Nucleotide Archive.

Since we found that *Blepharisma stoltei*, like *Blepharisma japonicum* (24), uses a non-standard genetic code, with UGA codon translated as tryptophan, gene predictions use the "The Mold, Protozoan, and Coelenterate Mitochondrial Code and the Mycoplasma/Spiroplasma Code (transl_table=4)" from the NCBI genetic codes. The default AUGUSTUS gene prediction parameters override alternative (mitochondrial) start codons permitted by NCBI genetic code 4, other than ATG. So, all predicted *B. stoltei* gene coding sequences begin with ATG.

RNA-seq read mapping relative to gene predictions of Contig_1 of *B. stoltei* ATCC 30299 was visualized with PyGenomeTracks (47).

Assessment of genome completeness

A BUSCO (version 4.0.2) (48) analysis of the assembled MAC genomes of *B. stoltei* and *B. japonicum* was performed on the set of predicted proteins (BUSCO mode -prot) using the BUSCO Alveolata database. The completeness of the *Blepharisma* genomes was compared to the protein-level BUSCO analysis of the published genome assemblies of ciliates *T. thermophila*, *P. tetraurelia*, *S. coeruleus* and *I. multifiliis* (Fig. S2A).

Gene annotation

Pannzer2 (49) (default parameters) and EggNog (version 2.0.1) (50) were used for gene annotation. Annotations were combined and are available from the Max Planck Society's Open Research Repository, Edmond (https://dx.doi.org/10.17617/3.8c). Protein domain annotations were performed using hmmscan from HMMER3 (version 3.3, Nov 2019) (42) vs. the PFAM database (Pfam-A.full, 33.0, retrieved on June 23, 2020) with default parameters.

Gene expression analysis

Features from RNA-seq reads mapped to the *B. stoltei* ATCC 30299 MAC and MAC+IES genomes over the developmental time-course were extracted using featureCounts from the Subread package (51). Further analysis was performed using the R software environment. Genes with a total read count of less than 50 across all timepoints were filtered out of the dataset. The remaining genes were passed as a DGElist object to edgeR (52). Each time point, representing one library, was normalized for library size using the edgeR function calcNormFactors. The normalized read counts were transformed into TPM (transcripts per million) values (53, 54). The TPM-values for different genes were compared across timepoints to examine changes in gene expression. Heatmaps showing log2(TPM) changes across timepoints were plotted using the tidyverse collection of R packages (https://www.tidyverse.org/) and RColorBrewer (https://rdrr.io/cran/RColorBrewer/). Tabulated gene expression estimates together with protein annotations are available from Edmond (https://dx.doi.org/10.17617/3.8c).

We eschewed a shallow Gene Ontology (GO) enrichment analysis, instead favoring close scrutiny of a smaller subset of genes strongly upregulated during new MAC formation. For this, computational gene annotations in combination with BLASTP searches and examination of literature associated with homologs was used.

Sequence visualization and analysis

Nucleotide and amino acid sequences were visualized using Geneious Prime (Biomatters Ltd.) (21). Multiple sequence alignments were performed with MAFFT version 7.450 (55, 56). Phylogenetic trees were constructed with PhyML version 3.3.20180621 (57).

Orthogroup inference and analysis of orthogroup clusters

OrthoFinder version 2.5.2 with default parameters (i.e., using Diamond for searching, MAFFT for multiple alignment and FastTree for phylogenies) was used to define orthogroups, i.e., sets of genes descended from the last common ancestor of the chosen species. Proteomes for the following ciliate species were used: *Tetrahymena thermophila*, *Oxytricha trifallax*, *Stentor coeruleus* (data from ciliate.org (58)); *Euplotes octocarinatus* (EOGD (59)); *Paramecium tetraurelia*, *Paramecium caudatum* (data from ParameciumDB (60)); plus *Perkinsus marinus* ATCC 50983 (GenBank accession: AAXJ00000000) as a non-ciliate outgroup. Orthogroup clusters are available as Data S2, or from Edmond (https://dx.doi.org/10.17617/3.8c).

Identification and correction of MIC-encoded PiggyBac homologs

We sought coding regions present within *Blepharisma* IESs to gauge the expression and type of MIC-limited genes (IES assembly and gene prediction described in Seah et al. 2022). After gene prediction within IESs with Intronarrator, predicted protein domains were annotated by HMMER (v3.3) (42). Several transposase families were represented in protein domains identified with coding regions of IESs. However, gene prediction within IESs was hampered by the presence of intermittent A-residues in the consensus sequence which occur due to the inaccuracy inherent in long-reads, from which the IES regions were assembled. These errors cause IES gene prediction to falter by generating inaccurate ORFs. To circumvent this, a six-frame translation of the MIC-limited genome regions was performed using a custom script, which was then used to detect PFAM domains, using HMMER and the Pfam-A database 32.0 (release 9) (61). Domain annotations for diagrams were generated with the InterproScan 5.44-79.0 pipeline (62)

Four instances of the Pfam domain DDE_Tnp_1_7, characteristic of PiggyBac transposases, were detected in an initial gene prediction within *Blepharisma* IESs. The four genes corresponding to the DDE_Tnp_1_7 domain had high RNA-seq coverage of combined reads from all timepoints across development. The IESs with the PiggyBac domains on Contig 17 and Contig 39 each had two ORFs with a partial DDE_1_7 domain, separated by a few hundred bp. Alignment of short-read MIC-enriched DNA reads mapped to the IES regions containing the putative PiggyBac homologs indicated that several A-nucleotides in the assembled IESs were insertion errors in the IES assembly, which were corrected with the short-read alignment. Open reading frames of predicted genes in these corrected regions were adjusted accordingly. The prefix “cORF” (corrected ORFs) was used to indicate the short-read corrected sequences of the PiggyMics.

Short-read MIC-enriched DNA sequences were aligned to the IES regions containing putative PiggyBac homologs with Hisat2 (2.0.0-beta) with modified source code (described in “RNA-seq read mapping”). Indel errors in the IES assembly were corrected manually, then used to predict coding regions. Pfam domains were annotated on MIC PiggyBac homologs with corrected ORFs using the InterproScan (v. 1.1.4) (63) plugin in Geneious v11.1.5 (Biomatter Ltd.). DDE_Tnp_1_7 domains were detected in the corrected ORFs, which in some cases spanned IES regions lacking predicted genic regions before correction. A multiple sequence alignment of the correct MIC PiggyBac homologs with other ciliate PiggyBac-derived proteins (PGBDs) and eukaryotic PiggyBac-like elements (PBLEs) that contain the PiggyBac transposase domain DDE_Tnp_1_7 (PF13843) was performed with MAFFT (v4.1) via the Geneious plugin (algorithm L-INS-i, BLOSUM62 scoring matrix, gap open penalty 1.53, offset value 0.123). A phylogenetic tree was constructed using the FastTree (v 2.1.11) plugin for Geneious (Whelan-Goldman model).

d_N/d_S estimation

We generated pairwise coding sequence alignments of PiggyMac paralog nucleotide sequences from *P. tetraurelia* and *P. octaurelia* using MAFFT version 7.450 (56) (55) (algorithm: "auto", scoring matrix: 200PAM/k=2, gap open penalty 1.53, offset value 0.123) using the "translation align" panel of Geneious Prime (version 2020.1.2) (21). PAML version 4.9 (64) was used to estimate d_N/d_S values in pairwise mode (runmode = -2, seqtype = 1, CodonFreq = 2). For *Blepharisma stoltei*, we generated pairwise coding sequence alignments of the *Blepharisma* PiggyMac homolog, BPgm (Contig_49.g1063; BSTOLATCC_MAC17466), with the *Blepharisma* Pgm-likes (BPgmLs) using Translation Align panel of Geneious v11.1.5 (Genetic code: *Blepharisma*, Protein alignment options: MAFFT alignment (v7.450) (56), scoring matrix: BLOSUM62, Gap open penaly: 1.53, offset value: 0.1). PAML version 4.9 was used to estimate dN/dS values in pairwise mode (runmode = -2, seqtype = 1, CodonFreq = 2).

Phylogenetic analysis

Protein sequences of PBLEs were obtained from Bouallègue et al (65). Protein sequences of *Paramecium* and *Tetrahymena* Pgms and PgmLs were obtained from ParameciumDB (60) (PGM, PGMLs1-5) and ciliate.org (58) (Tpb1, Tpb2, Tpb7, LIA5), respectively. *Condylostoma* and *Blepharisma* Pgms and PgmLs were obtained from genome assemblies (accessions GCA_920105805 and GCA_905310155, respectively). Sequence organization and manipulation was done using Geneious (Biomatters Ltd.). The Geneious plug-in for InterProScan (62) was used to identify DDE_Tnp_1_7 domains using the PFAM-A database (61). The DDE_Tnp_1_7 domain and regions adjacent to it were extracted and aligned using the MAFFT plug-in (v7.450) for Geneious (56) (algorithm: L-INS-i, scoring matrix: BLOSUM62, gap opening penalty: 1.53, offset value: 0.123). Phylogenetic trees using this alignment were generated with the FastTree2 (v2.2.11) Geneious plug-in using the Whelan-Goldman model. The phylogenetic trees were visualized with FigTree (v1.4.4) (Andrew Rambaut, http://tree.bio.ed.ac.uk/).

Repeat annotation

Interspersed repeat element families were predicted with RepeatModeler v2.0.1 (default settings, random number seed 12345) with the following dependencies: rmblast v2.9.0+ (http://www.repeatmasker.org/RMBlast.html), TRF 4.09 (Benson, 1999), RECON (Bao and Eddy, 2002), RepeatScout 1.0.6 (Price et al., 2005), RepeatMasker v4.1.1 (http://www.repeatmasker.org/RMDownload.html). Repeat families were also classified in the pipeline by RepeatClassifier v2.0.1 through comparison against RepeatMasker's repeat protein database and the Dfam database. Consensus sequences of the predicted repeat families, produced by RepeatModeler, were then used to annotate repeats with RepeatMasker, using rmblast as the search engine.

Terminal inverted repeats (TIRs) of selected repeat element families were identified by aligning the consensus sequence from RepeatModeler, and/or selected full-length elements, with their respective reverse complements using MAFFT (Katoh and Standley, 2013) (plugin version distributed with Geneious). TIRs from the Dfam DNA transposon termini signatures database (v1.1, https://www.dfam.org/releases/dna_termini_1.1/dna_termini_1.1.hmm.gz) (Storer et al., 2021) were searched with hmmsearch (HMMer v3.2.1) against the IES sequences, to identify matches to TIR signatures of major transposon subfamilies.

SI Results

Additional assembly considerations and inspection

Compared to assemblies of independent, replicate libraries with 70× or 76× coverage there was a modest improvement (e.g., from 86 and 89 contigs to 74) in assembly contiguity for the assemblies produced at 145× coverage (Table S2). With increasing sequence depth, reads of micronuclear origin could conceivably start linking MAC chromosomes and extending their ends, even though we depleted MIC DNA by sucrose gradient centrifugation of nuclei.

We chose to conservatively trim back the ends of most of the contigs in the assembly, and break apart a few contigs at internal sites. This was done either where the coverage locally decreased or increased and where there were extensive differences between the contig sequence and mapped reads. Some of these sequences could represent an intermediate state between fully retained and fully eliminated DNA in MACs. 1.3 Mb of such uncertain sequences (termed "cruft" in genome assembly terminology) were removed from the main assembly. No tRNA genes were predicted in the cruft sequences, nor did their removal reduce the BUSCO score for completeness of the MAC genome, which is comparable to or better than that of other ciliates (Fig. S2A). BUSCO analyses also showed that gene duplication in *Blepharisma*, though common, is lower than in *Paramecium tetraurelia* and *Stentor coeruleus* (Fig. S2A).

Telomeres in *Blepharisma*

The basic telomere unit of *Blepharisma* is a permutation of CCCTAACA, like its heterotrich relative *Stentor coeruleus* (66) (Fig. S4D). Since a compelling candidate for a telomerase ncRNA (TERC) could not be found in either *Blepharisma* or *Stentor* using Infernal (44) and RFAM models (RF00025 - ciliate TERC; RF00024 - vertebrate TERC), it was not possible to delimit the repeat ends. Heterotrichs may use a different or very divergent ncRNA. In contrast to the extremely short (20 bp) MAC telomeres of spirotrichs like *Oxytricha* with extreme MAC genome fragmentation (67), sequenced *Blepharisma* MAC telomeres are moderately long (Fig. S4B), with a mode of 209 bp (~26 repeats of the 8 bp motif), extending to a few kilobases.

Alternative telomere addition sites (ATASs)

Alternative telomere addition sites in the MAC genome tend to be intergenic in model ciliates like *Oxytricha trifallax* (67). In *Blepharisma*, we found more intergenic ATASs (28,309) than intragenic ones (18,396). As intergenic regions only make up 10.1 Mb of the assembly, the intergenic frequency of ATASs is about five-fold higher (2.81 per 1 kb) than intragenic frequency (0.562 per 1 kb). The presence of intragenic ATASs raises the question how the cell tolerates or deals with mRNAs encoding partial proteins transcribed from 3' truncated genes. Since the sequence data was from a clonal population, it is not possible to tell how much ATAS variability there is within individual cells. However, it is conceivable that their positional variation in single cells reflects that of the population. In this case, together with redundancy from massive DNA amplification there would likely be sufficient intact copies of every gene.

Beyond the first 2-5 bp corresponding to the junction sequences, the average base composition on the chromosome flanking ATAS junctions shows an asymmetrical bias (Fig. S4G). From position +6 onwards there is an enrichment of T to about 40% and A to 35-39%, compared to the genome-wide frequencies of 33% each. At position +19 to +23, there is a slight decrease in T to 37-39%. AT values gradually decline back to about 35% each by position +150. Correspondingly, G and C are depleted downstream of ATAS junctions, dropping to a minimum of 8.6% and 11% respectively around position +37, compared to the genome-wide average of 17% each. AT enrichment and GC depletion upstream of ATAS junctions are less pronounced.

If breakage and chromosome healing were random, we would not expect such an asymmetry. This suggests that there is a nucleotide bias, whether in the initiation of breaks, telomere addition, or in the processing of breaks before telomere addition. However, we have not yet identified any conserved motif like the 15 bp chromosome breakage site (CBS) in *Tetrahymena* (68) nor a short

10-bp sequence periodicity in base composition like in *Oxytricha trifallax* (69). Therefore, telomere addition in *B. stoltei* appears to involve base-pairing of short segments of about 2 bp between the telomere and chromosome, with a bias centered on the "CT" in the telomere unit, and an asymmetrical preference for AT-rich sequences on the chromosomal side of the junction.

The position of an ATAS junction is potentially ambiguous because the last adjoining telomere repeat can potentially be extended into the chromosomal sequence, if the chromosomal sequence at the junction contains a partial match to the telomere (Fig. S4E). The junction position that maximizes the length of the telomere sequence on a read has been termed as the "first identifiable breakpoint", and that which maximizes the chromosomal sequence as the "last identifiable breakpoint" (70). The overlapping sequence, which could either be telomeric or chromosomal, is termed the "junction sequence".

Most ATAS junctions in *B. stoltei* have an overlapping junction sequence, on average 2-3 bp long (Fig. S4I). This can also be observed when separate sequence logos are drawn for each of the possible telomere repeat permutations observed at the ATAS junction (Fig. S4F). Such a short overlap of a few base pairs between the telomere repeat and chromosome sequence is similar to what has been observed in other organisms, such as 3-5 bp in yeast (70) and 2-4 bp in humans (71). This is in contrast to *Tetrahymena* where telomeres are often added to sites that have no homology to the telomere sequence (72).

We hypothesized that the location of ATAS junctions in the genome might be randomly distributed and simply reflect the baseline sequence composition of the genome and/or the telomeres. To test this, we counted the frequency of 2-mers in the MAC genome (excluding telomeric regions) and in the telomere repeats, and compared them to the 2-mer frequencies observed at ATAS junctions (2 bp on chromosomal side of last identifiable breakpoints, Fig. S4H). Sequence composition of the telomeres does have a strong influence, as 2-mers that are not represented in the telomeres (AT, GC, CG, GA) are poorly represented at ATAS junctions even though they may be frequent in the genome, e.g., GA, 12.0% in genome vs. 0.36% at ATAS; AT, 10.4% vs. 1.7%. However, 2-mer frequencies at ATAS junctions do not match frequencies in the telomeres closely either. For example, the 2-mer AG is about twice as frequent at ATAS junctions as compared to telomeres, and as compared to the genome generally. Instead, the telomere permutations at ATAS junctions are not uniformly distributed; the permutation CTAACACC is the most common, followed by its adjacent permutations TAACACCC and AACACCCT (using last identifiable breakpoints, Fig. S4D). These would account for the three most common 2-mers at ATAS junctions: AG (canonical form of CT), AA, and TA.

Telomere-binding protein paralogs

Despite the abundance of *Blepharisma* MAC genome telomeres, we did not detect a typical ncRNA gene corresponding to the telomerase RNA component (TERC) of the ribozyme responsible for telomere synthesis in the MAC genome. We suspect this is due to ncRNAs presenting a far greater challenge to detect than protein-coding genes and the presence of highly divergent ncRNA with insufficient similarity to the handful of taxonomically-restricted TERCs identified in oligohymenophorean and spirotrich ciliates and other eukaryotes so far.

Other than the components of telomerase, ciliates were among the first organisms where telomere-binding proteins were characterized (73). Telomere-binding protein paralogs with distinctive patterns of gene expression during development are present in some ciliate species (67, 74). *Tetrahymena thermophila* has two telomere-binding protein paralogs POT1 and POT2 (74). POT2 is upregulated during conjugation, accumulates in developing new macronuclei, and binds to chromosome breakage sites rather than telomeres (74). *Blepharisma stoltei* has five POT1 paralogs POT1.1-POT1.5 (Fig. S5C). One *B. stoltei* POT1 paralog expressed at low levels in starved (0 h) cells, POT1.4, is sharply upregulated during development, peaking when new macronuclei are forming (22 h) (Fig. S5C).

Since we were unable to identify a specific chromosome breakage signal like that of *Tetrahymena*, a future avenue to search for such a signal would be to assess the DNA-binding preferences of POT1.4 and the other *Blepharisma* POT1 paralogs. In any event, since this is one of the most highly upregulated genes in the 22-26 h time range compared to vegetative (0 h, and gamone treated cells; see “Results”, “Features of gene expression during new MAC development”), future investigation of its developmental role is warranted.

Tiny spliceosomal introns

Like *Stentor* (66), most (82%) *Blepharisma* genes have no introns. In line with genome compactness, during our inspections we also observed numerous overlapping poly(A)-tailed RNA-seq reads on opposite strands derived from convergently transcribed gene pairs. The correlation of the lengths of different noncoding region classes (intergenic regions, introns and UTRs) can be explained by them being subject to common, neutral evolutionary processes (75).

Blepharisma introns are mostly (97%) 15 or 16 nucleotides (nt) long, like those of *Stentor* (Fig. S3D). Though intron reduction (7,389 introns predicted in the reference *B. stoltei* MAC genome, i.e., 0.29 introns per gene) is not as extreme as some other microbial eukaryotes, like *Giardia lamblia* (76), where almost all have been lost, both *Blepharisma* and *Stentor* have much fewer introns relative to other ciliates (e.g., intron densities of 1.6, 2.3 and 4.8 introns per gene in *Paramecium*, *Oxytricha* and *Tetrahymena*, respectively (77)) and to the putative, relatively intron-rich eukaryotic common ancestor (78), along with their extreme length reduction.

Blepharisma 15 nt introns possess a characteristic branch-point “A”, as would be expected in classical models of lariat formation during mRNA splicing (Fig. S3C). 16 nt introns almost invariably have an “A” at either 10 or 11 nt downstream of the donor site (i.e., only one of 499 does not, but has “A” at 9 nt), although this is not obvious in the consensus sequence logo because the position is variable (Fig. S3D). Similarly, 17 nt introns all possess “A” at 10-12 nt downstream of the donor site. Only a few intron bases, 5-8 and 12, of *Blepharisma*’s 15 nt introns are relatively unconstrained (Fig. S3C). This leaves little room for the presence of any additional regulatory elements in the mRNA or underlying DNA.

In the final gene predictions, just over 1% of predicted *Blepharisma* introns lack canonical GT-AG boundaries (62 out of 4,670 introns). Just under half of these (30) are 15 or 16 bp long and predominantly appear to represent true spliceosomal introns. The boundaries of two predicted introns with CT-AC boundaries (14 and 15 nt in length) resulted from misalignment of nucleotides in the mapped spliced reads at conventional GT-AG junctions. We found no evidence of minor spliceosomal RNAs (U11, U12, U4atac, and U6atac) using Infernal searches (E-value < 10). Thus, *Blepharisma* appears to lack a minor spliceosome and minor spliceosomal introns. As far as we are aware no minor spliceosomal introns have been reported in any ciliates. Loss of minor spliceosomal machinery and introns, relative to the eukaryotic common ancestor, may be relatively common in alveolates including ciliates (79).

The most common 5’ boundaries for *Blepharisma* introns that possess a 3’-AG but lack 5’-GT are 5’-GC or 5’-GG (the latter are most often 5’-GGT; Table S9). Introns that possess a 5’-GT but lack 3’-AG typically have 3’-GG boundaries (most often 3’-AGG; Table S9). Visual inspection of the mapped RNA-seq data to the non-canonical *Blepharisma* introns and predicted coding sequences suggests that the GC-AG, GT-GG and GG-AG introns are correct, i.e., lead to prediction of complete coding sequences downstream of their locations. Lower frequency alternative splicing may occur in some cases (e.g., Fig. S3G), but these generate prematurely terminated coding sequences.

GC-AG introns are the most common alternative major spliceosomal introns in multicellular organisms (80). In *Blepharisma* such introns are most frequently 15 bp long. In contrast to GC-AG introns and conventional *Blepharisma* GT-AG introns, GG-AG and GT-GG (or GGT-AG and GT-AGG) introns are 16 bp or longer (Table S9). This suggests most of these introns evolved from conventional 15 bp GT-AG introns. It is possible that splicing of the shorter internal GT-AG

introns, instead of their longer non-canonical forms that give rise to full-length coding sequences, leads to NMD of some mRNAs, since these invariably have a premature in-frame stop codon downstream of the intron. Thus RNA-seq may underestimate the amount of splicing of the shorter forms.

Overcoming challenges in gene prediction due to tiny introns

As reported in *Stentor*, splicing frequency decreases as intron length increases in *Blepharisma* (Fig. S3B). This trend is also evident in antisense introns, though weaker and more noisy due to their lower abundance (Fig. S3B). Since antisense intron splicing would be free from selective constraints imposed by protein-coding sequence translation, we suggest that the intron length distribution primarily reflects the splicing length preferences of the spliceosome. The decreased efficiency of splicing of introns longer than 15 nt, and evident inability to splice introns shorter than this, means that most intron indels may be deleterious. We therefore suggest that, like its IESs which are skewed towards shorter lengths (Seah et al. 2022), *Blepharisma*'s introns can largely be thought of as parasitic elements which bear significant potential costs. This would also be consistent with the absence of introns in most heterotrich genes, and a pronounced decrease in intron density relative to model ciliates such as *Paramecium*, *Tetrahymena* and *Oxytricha* (66).

The tiny introns of *Stentor coeruleus* previously created significant challenges for gene prediction (66) using AUGUSTUS (81). In the *Stentor* study, some predicted genes were observed to be incorrectly joined, and so were split with a custom script. Furthermore, introns of lengths other than 15 or 16 bp were attributed to genome mis-assembly (66). In our study, after adjusting AUGUSTUS parameters for tiny introns as for *Stentor* and training AUGUSTUS for gene prediction in *Blepharisma*, from visual inspection of mapped RNA-seq reads we saw that most predicted introns longer than 16 bp are incorrect. With the benefit of major technological advances in long read sequencing and considerably increased sequencing depth over the last years, the *Blepharisma* MAC genome assembly is not as prone to misassembly, and contiguity substantially improved compared to that of the draft *Stentor coeruleus* assembly. Consequently, most of the incorrect introns predicted with AUGUSTUS in *Blepharisma* were errors in gene prediction rather than mis-assembly. Additionally, numerous introns, including some of length 15 or 16 nt, were predicted in regions deeply covered by RNA-seq with no mapped reads evidencing splicing. No matter what changes we attempted to the AUGUSTUS source code in attempts to more accurately predict introns, more were incorrectly predicted than not (e.g., Table S8).

Since we obtained extensive RNA-seq data across a developmental time course which appeared to cover most genes (Fig. 3A), we chose to eliminate incorrect intron predictions, the major source of inaccuracy in *Blepharisma* gene predictions, by directly predicting introns using mapped reads. This approach, Intronarrator, runs AUGUSTUS in "intronless" prediction mode on a version of the genome with introns removed, before replacing the introns in the genes. Visual inspection of the predicted introns on Contig_1, showed there was a marked improvement in intron prediction sensitivity from 0.75 with AUGUSTUS to 0.97 with Intronarrator, while precision improved from 0.42 to 1.00 (Table S8). In general, there is consistency between the locations of the predicted genes and RNA-seq coverage, notably including genes with introns (Fig. 3).

Extensive duplications of transmembrane protein genes

A notable extended ~220 kb region encoding 53 genes belonging to a single orthologous group (orthogroup), OG0000085 is present on Contig_1 (Fig. 3A). Four additional OG0000085 genes are present at the opposite end of Contig_1, and 24 copies are found on other contigs, often clustered together (Fig. S1B). The DNA coverage across this region is lower (74×) than the rest of Contig_1 (185×). Though there is uncertainty in the exact extent, given the sheer volume of reads involved, the assembled sequences certainly correspond to highly repetitive regions of the MAC genome. At the junction between the lower and higher coverage regions more than 30 HiFi reads link the two regions of coverage, and a similar number of telomere-bearing reads are in close proximity. At the junction we also observe at least two potential locations of IESs, corresponding to regions that may be partially IES/partially MDS.

Large clusters of genes from particular orthogroups can be found on additional contigs (Fig. S1B). In total 551 (2%) of predicted *B. stoltei* genes belong to the orthogroups with the largest clusters per contig. Some of the largest contiguous clusters of genes from these orthogroups are situated at the ends of contigs, suggesting they may have caused assembly breaks beyond them. One contig, split off from other connected components in the assembly graph, predominantly encodes genes from a single orthogroup (contig_64, 43× coverage; Fig. S1B). Further increases in read length and accuracy may allow assemblers to fully resolve these in future. Curiously, all the orthogroups corresponding to the largest contiguous clusters of genes appear to be transmembrane proteins, or decayed remnants thereof.

Full-length proteins from OG0000085 (81 proteins in total) and OG0000014 (143 proteins in total) both contain a central PFAM “ANF_receptor” domain (PF01094), annotated in the PFAM database with the description “This family includes extracellular ligand binding domains of a wide range of receptors”. Though apparently distantly related (32% amino acid identity of the consensus sequences; produced by the majority rule for each orthogroup), the full-length proteins are of similar length and align well, and thus are likely homologs. A clear C-terminal transmembrane domain region comprising seven to nine alpha helices is predicted for presumed full-length versions of proteins from both ortholog groups using TMHMM2 (82). Queries of UniProt revealed that, though widely distributed among eukaryotes, among ciliates only *Stentor coeruleus* also possesses proteins with this domain classified (29 in total). BLAST searches versus the GenBank NR database detect a similar number of matches to *Stentor coeruleus* homologs (E-value < 1e-30) but none in any other ciliates. Ortholog groups OG0000018 and OG0000052 also appear to be homologous to one another (31% amino acid identity of the consensus sequences produced by the majority rule for each orthogroup). Full-length proteins from these ortholog groups possess a clear N-terminal transmembrane domain predicted by TMHMM2, composed of seven or more transmembrane helices. We also detected a central Pas domain (PF00989) in a couple of these proteins in InterProScan searches of PFAM. Ortholog group OG0000019 has a seven transmembrane C-terminal domain predicted by TMHMM2, a series of centrally located “Laminin_G_3” (PF13385) domains, and an N-terminal “Malectin” (PF11721) domain in some proteins.

Ciliates encode a moderately large number of protein-coding genes compared to other eukaryotes, often exceeding 25,000. Species like *Paramecium tetraurelia* which have undergone multiple whole genome duplications, may have more than 40,000 genes (83). In ciliate species like *Tetrahymena thermophila* (26,258 genes (32)), with no evidence of whole genome duplications, it has been a question as to why these species are so gene rich (84).

Segmental duplications identified in the human genome are defined as duplications > 1 kb and > 90% sequence identity (85). Little evidence for such duplications was found in the *Tetrahymena* MAC genome (84). Since the divergences of the proteins within the large clustered *Blepharisma* orthogroups are typically moderately high (e.g., < 40% amino acid identity), the duplications that led to them represent older events. Nonetheless, given their extent, it is likely that many of the duplicated genes originated from segmental duplications. Recombination of clusters of some of these genes into other genomic regions may subsequently have spread them elsewhere, and led to gradual erosion of the original locus. The OrthoFinder algorithm is specifically designed to eliminate scoring biases against shorter sequences, a significant advance over older algorithms like OrthoMCL (86). In our inspections of multiple sequence alignments of the largest orthogroups we also detected numerous genes that are clearly related to, but significantly shorter than the typical gene length of each orthogroup, thus likely representing eroding pseudogenes. Though we focused on the largest and most notable clusters of genes from the orthogroups, numerous other genes may also have arisen out of such clustered duplications.

Development-specific upregulation of proteins associated with DNA repair and chromatin

A variety of different DNA repair protein genes strongly upregulated at 26 hours (Table S4; Data S3) are: a 5’ Apollo exonuclease protein (BSTOLATCC_MAC16643), whose homologs are

involved in DNA repair and telomere protection (87) (a paralog of this gene is constitutively expressed at low levels: BSTOLATCC_MAC3725; 58.9% pairwise amino acid identity); STAG1/2 (BSTOLATCC_MAC22820) and Rad21 (BSTOLATCC_MAC1548) homologs, both cohesin complex components, and a Rad50 homolog (BSTOLATCC_MAC2159), all proteins involved in DNA double-strand break repair (88, 89); a homolog of MUS81 (BSTOLATCC_MAC21072) a protein involved in meiotic double-strand break repair in *Tetrahymena* (90); a homolog of PARP2 (Poly(ADP-ribose) polymerase-2) (BSTOLATCC_MAC1058), a protein involved in DNA single-strand nick repair (91); a homolog (BSTOLATCC_MAC1470) of the DNA clamp, PCNA, which is involved in DNA repair associated with DNA polymerases delta and epsilon (92, 93); two homologs (BSTOLATCC_MAC23155 and BSTOLATCC_MAC23646) of exodeoxyribonuclease III, a protein involved in abasic DNA base repair (94).

A dozen chromatin-related proteins are among the top 100 most strongly upregulated proteins at 26 hours (Table S4). These include a homolog (BSTOLATCC_MAC17684) of ISWI, a core ATPase remodeler present in a range of different chromatin remodelling protein complexes in eukaryotes (95). In *Paramecium tetraurelia* the strongest developmentally upregulated ISWI homolog plays a critical role in nucleosome positioning in new MACs during genome editing (96). A few histone/histone-related proteins and HMG boxes are also strongly upregulated. Two JmjC (Jumonji C) domain-containing proteins are also highly upregulated (BSTOLATCC_MAC23590 and BSTOLATCC_MAC5044). BSTOLATCC_MAC23590 is likely to be orthologous to JMJ1 of *Tetrahymena thermophila* (TTHERM_00185640): they are reciprocal best BLASTP hits (with next best hit e-values many orders of magnitude higher), the JmjC domain occurs in a similar relative location in the two proteins, and their lengths are similar (1082 aa and 1198 aa). JMJ1 is highly upregulated during *T. thermophila* conjugation, first localizing in old MACs and later in the new MACs (97). This protein is required for H3K27me3 demethylation later in conjugation, where it is proposed to influence gene expression, including those expressed later and involved in genome editing processes, rather than heterochromatin associated with *Tetrahymena* IES excision per se (97).

Development-specific upregulation of proteins associated with initiation of transcription and translation

While overarching coordination of gene regulation and protein translation are expected during ciliate development, it is not evident how this might be achieved. Among the most strongly upregulated genes at 26 hours are homologs of proteins involved in initiation of either transcription or translation, notably an eIF4E translation initiation factor homolog (BSTOLATCC_MAC5291) and a TATA-binding protein (SPT15; BSTOLATCC_MAC11469; Table S4). In other eukaryotes eIF4E proteins bind to m7G 5' mRNA caps permitting protein translation (98). *B. stoltei* has ten homologs of these proteins, nine of which are moderately stably expressed throughout the RNA-seq conditions examined (Data S3; workbook "eIF4e homologs"). eIF4E paralogs are also abundant in *S. coeruleus*, with thirteen homologs found by BLASTP. One of the *B. stoltei* eIF4E paralogs is more highly expressed than the rest (BSTOLATCC_MAC25346), however this is still roughly an order of magnitude less than the development-specific paralog in all times after 2 hours post cell mixing. The pronounced upregulation of a homolog of eIF4e would be consistent with the massive amount of protein translation necessary during development. We therefore propose that translation initiation plays a critical regulatory role in protein synthesis during *Blepharisma* development, all the way through genome editing.

Regarding transcription regulation, *B. stoltei* has a constitutively expressed TATA-binding protein (BSTOLATCC_MAC16553) which is 64.8% identical (at the amino acid level) to the developmentally upregulated paralog (BSTOLATCC_MAC11469). *Tetrahymena thermophila* also appears to have two TATA-binding protein homologs annotated (TBP1 and TBP2; 30.5% pairwise amino acid identity) both of which are modestly upregulated during development (http://tfgd.ihb.ac.cn/search/detail/gene/TTHERM_00575350 and http://tfgd.ihb.ac.cn/search/detail/gene/TTHERM_00082170). In *B. stoltei* we speculate that the two TATA-binding proteins may recognize distinct TATA box motifs, and thus transcription of a large, development-specific subset of proteins might be controlled by a master regulator. A

homolog of transcription initiation factor TFIID subunit 1 (TAF), the largest core component of the transcription initiation complex (99) that interacts with TATA-binding proteins (100) is encoded by the sixth most strongly upregulated gene (388×) at 26 hours (BSTOLATCC_MAC12987). The only other TAF homolog we detected (BSTOLATCC_MAC10371) is more weakly upregulated (12×) at 26 hours.

In *B. stoltei* an additional homolog of a protein involved in transcription elongation (SPT5; BSTOLATCC_MAC7803) is among the most highly upregulated genes at 26 hours, and also has a constitutively expressed paralog (BSTOLATCC_MAC18233 78.4% pairwise amino acid identity). *Paramecium tetraurelia* and *Oxytricha trifallax* both have SPT5 paralogs that appear to have been generated in separate duplication events (101), and our phylogenies suggest the *B. stoltei* paralogs duplicated independently of these two species. In *P. tetraurelia*, one of the two paralogs is specific to meiotic micronuclei, and has an expression profile that peaks earlier during development prior to meiosis and declines during new MAC formation (101). In *Oxytricha* one SPT5 paralog (SPT5a) is constitutively expressed, whereas the other (SPT5b) peaks during meiosis (102). In *Tetrahymena* the single SPT5 gene is strongly upregulated during development, peaking during meiosis (http://tfgd.ihb.ac.cn/search/detail/gene/TTHERM_00028580).

Cysteine-rich domain of the *Blepharisma* PiggyBac homologs

PiggyBac CRDs have been classified into three different groups and are essential for *Paramecium* IES excision (103). In *Blepharisma*, the CRD consists of five cysteine residues arranged as CxxC-CxxCxxxxH-Cxxx(Y)H (where C, H, Y and x respectively denote cysteine, histidine, tyrosine and any other residue). Two *Blepharisma* homologs possess this CRD without the penultimate tyrosine residue, while the third contains a tyrosine residue before the final histidine. This -YH feature towards the end of the CxxC-CxxCxxxxH-Cxxx(Y)H CRD is shared by all the PiggyBac homologs we found in *Condylostoma*, the bat PiggyBac-like element (PBLE) and human PiggyBac element-derived (PGBD) proteins PGBD2 and PGBD3. In contrast, PiggyBac homologs from *Paramecium* and *Tetrahymena* have a CRD with six cysteine residues arranged in the variants of the motif CxxC-CxxC-Cx{2-7}Cx{3,4}H, and group together with human PGDB4 and *Spodoptera frugiperda* PBLE (Fig. S8).

***Blepharisma*'s MAC genome encodes additional domesticated transposases**

Three *Blepharisma* MAC genome-encoded proteins possess PFAM domain DDE_1 (PF03184; Fig. S9). The most common domain combinations for this domain, aside from proteins with it alone (5898 sequences; PFAM version 35), are with an N-terminal PFAM domain HTH_Tnp_Tc5 (PF03221) alone (2240 sequences), and both an N-terminal CENP-B_N domain (PF04218) and central HTH_Tnp_Tc5 domain (1255 sequences). The CENP-B_N domain is characteristic of numerous transposases, notably the Tigger and PogoR families (104). Though pairwise sequence identity is low amongst the *Blepharisma* DDE_1-proteins (avg. 28.3%) in their multiple sequence alignment, the CENP-B_N domain in one of them appears to align reasonably well to corresponding regions in the two proteins lacking this domain, suggesting it deteriorated beyond the recognition capabilities of HMMER3 and the given PFAM domain model. BLASTp matches for all three proteins in GenBank are annotated either as Jerky or Tigger homologs (Jerky transposases belong to the Tigger transposase family (104)). Given that none of the *Blepharisma* MAC DDE_1-domain proteins appear to have a complete catalytic triad, it is unlikely they are involved in transposition or IES excision. In *Blepharisma* and numerous other organisms, DDE_1 domains co-occur with CENPB domains. Two such proteins represent totally different proposed exaptations in mammals (centromere-binding protein) and fission yeast (regulatory protein) (105–107). Given the great evolutionary distances involved, there is no reason to expect that the *Blepharisma* homologs have either function.

Six MAC-encoded transposases containing the DDE_3 domain (PF13358) are present in *Blepharisma*, all of which are substantially upregulated in MAC development and five of which possess the complete DDE catalytic triad (Fig. S9B). The DDE_3 domain is characteristic of DDE transposases encoded by the Telomere-Bearing Element transposons (TBEs) of *Oxytricha*

trifallax (108, 109), which, despite being MIC genome-limited, are proposed to be involved in IES excision (110). DDE_3-containing transposons, called Tec elements, are found in another spirotrichous ciliate, *Euplotes crassus*, but no role in genome editing has been established for these (111). TBEs and Tec elements do not share obvious features, other than both possessing an encoded protein belonging to the IS630-Tc1 transposase (super)-family (112). All six *Blepharisma* DDE_3 genes have at least 150× HiFi read coverage, consistent with their presence in bona fide MAC DNA.

As judged by BLASTP searches in which most of the top hundred best matches are classified are “IS630 family” transposases, *Blepharisma* MAC-encoded DDE_3 domain transposases are more closely related to the IS630 transposase family than to *Oxytricha* TBE transposases and *Euplotes* Tec transposases. One of the BLAST top hits is a MIC genome-encoded protein in *Oxytricha trifallax* with a DDE_3 domain which is not a TBE transposase (GenBank accession: KEJ83017.1). IS630 transposases diverge considerably from Tc1-Mariner transposases, and hence are considered an outgroup to them (113). IS630-related transposases encoded by Anchois transposons have also been detected in the *Paramecium tetraurelia* MIC genome (114). Given that all but one of the *B. stoltei* paralogs appear to possess a complete catalytic triad, there is a possibility that they may be involved in some IES exicison.

Among other ciliates with draft MAC genomes we examined, the IS1595- and MULE transposase-like domains (PFAM PF12762 and PF10551) have so far only been observed in the spirotrichs *Oxytricha* and *Stylonychia* (67, 115). DDE_Tnp_IS1595 domains are characteristic of the Merlin transposon superfamily and MULE is part of the Mutator transposon superfamily (116). Currently no particular functions have been demonstrated for these proteins in these ciliates, but their genes were substantially upregulated during their development (67, 117). Both transposase-like domains are found in MAC-encoded proteins in *Blepharisma* and their underlying genes are upregulated during MAC development (Figs. S9C, S10). Consistent with the notion of transposase domestication, the genes encoding DDE_Tnp_IS1595 and MULE proteins appear to lack flanking transposon terminal inverted repeats. Members of both IS1595 and MULE transposases also appear to have complete catalytic triads.

Homologs of small RNA-related proteins involved in ciliate genome editing

Development-specific proteins responsible for small RNA (sRNA) generation and transport play an important role in ciliate genome editing (118). In ciliates such as *Paramecium* and *Tetrahymena* shorter Dicer-like proteins (Dcls) are distinguished from longer Dicer proteins (Dcrs) which possess additional N-terminal domains and produce small RNAs, notably siRNAs, involved in gene regulation (119). In the scanning model of MAC development in *Tetrahymena* and *Paramecium*, Dcls cooperate with Piwi proteins, converting long double-stranded RNA transcripts produced in the maternal MIC into “scan RNAs” (scnRNAs) (119–124). Piwi-bound scnRNAs are transported to the maternal MAC where a subtractive process takes place, leaving only scnRNAs complementary to the MIC-limited genome. The remaining scnRNAs are transported to the new, developing MAC, where they target MIC-limited regions for excision (119–124).

We found putative Dicer, Dicer-like and Piwi proteins encoded by the *B. stoltei* MAC genome (Fig. S7). The single *B. stoltei* Dicer (Dcr) protein has the characteristic N-terminal Dicer domains followed by a pair of RNase III domains (PFAM domain Ribonuclease_3; PF00636) whereas RNase III domains alone were detected in three Dicer-like proteins (Dcl1-3). Dcl1 expression is upregulated shortly after conjugation begins and before meiosis begins; Dcl2 and Dcl3 are upregulated from meiosis onwards, peaking during anlagen formation. In *Paramecium* two Dcl’s are coexpressed and cooperate to produce scnRNAs (119), and so we predict that, as for *Paramecium*, *Blepharisma* Dcl2 and Dcl3 may cooperate.

B. stoltei also appears to have an additional truncated Dcr homolog (881 aa), Dicer-derived protein (Dcrd), lacking the RNase III domain portion (BSTOLATCC_MAC8391) of the complete Dicer (Fig. S7A). A short protein (690 aa) with a similar domain structure is found in *Paramecium tetraurelia* (Genbank accession: XP_001459306.1), and, as judged from gene expression data in

ParameciumDB (60), is substantially upregulated during new MAC development. The observation of these proteins suggests that it might be possible for Dicer helicase and cleavage activities to be encoded on separate molecules. The splitting of the helicase and RNAse domains is the converse of the common eukaryotic origin of Dicer from separate archaeal (helicase) and bacterial (RNase) domains (125). It is also conceivable, once they have split, that alternative helicases may substitute the original ones of helicase-less Dicer-like proteins.

In ciliates some Piwi proteins play a role in gene regulation in vegetative cells (126) while others are involved in genome editing (120, 127, 128). In *Stylonychia lemnae*, the massive upregulation of a Piwi homolog involved in genome editing allowed it to be identified by subtractive hybridization of RNA (129). The ortholog of this gene in *Stylonychia lemnae*'s close relative, *Oxytricha trifallax*, is also one of the most highly transcribed and upregulated genes (128). We found nine proteins with Piwi and PAZ domains (five of which also have ArgoL domains) in the *B. stoltei* ATCC 30299 MAC genome. Two closely related *Blepharisma* Piwi paralogs are highly upregulated during meiosis and throughout subsequent development (Fig. S7B). These two genes are both among the most highly expressed genes at 26 h (12 and 154) while the new MAC is forming.

In *Tetrahymena* and *Paramecium*, massive production of scnRNAs, generated by the Dcls and highly upregulated Piwis, initiates from meiotic nuclei. We observe a similar pattern of massive production of development-specific sRNAs during development, whose detailed analysis will be reported in conjunction with the draft *B. stoltei* ATCC 30299 MIC genome (Seah, et. al, 2022). Since *Blepharisma* species are distantly related to other ciliates whose sRNAs have been characterized, this suggests that an ancient, development-specific sRNA gene expression program may have been established in the ciliate common ancestor.

Development-specific histone variant upregulation

Access to DNA in eukaryotes is mediated by nucleosomes and nucleosome regulation plays a central role in DNA replication, repair, transcription and recombination (130). The nucleosome is composed of four core histone proteins, H2A, H2B, H3 and H4, and is held together by electrostatic interactions between the negative charge of the phosphate backbone of DNA and the positively charged surface of the histones (131). The modification of core histones by acetylation and methylation is involved in allowing or repressing access to the DNA. Genome rearrangement in ciliates is influenced by processes that modify and regulate nucleosomes and consequently mediate the ability of IES-excision machinery to access the underlying DNA. In *Tetrahymena*, IESs, which frequently contain transposons or are derived from them, are targeted for removal by sRNA machinery that is involved in depositing methylation marks on Histone 3 Lysine 9 (H3K9) and Histone 3 Lysine 27 (H3K27), in a process akin to heterochromatin formation, except that the marked regions are excised entirely (132, 133). In *Paramecium*, a mechanism reflecting the ancient ancestral eukaryotic origins of transposon silencing by heterochromatin formation, involving H3K9- and H3K27-trimethylation (H3K27me3), represseses MIC genome-encoded transposable element gene expression, and experimental elimination of these marks leads to low efficiency of IES excision and lethal outcomes when new MAC genomes are produced (134). A particular histone variant (H3.4) present in polytene DNA, was proposed to be the target of trimethylation, facilitating heterochromatinization and excision of IESs not protected by 27 nt macRNAs in the ciliate *Stylonychia* (135).

We annotated the four core histones, H2A, H2B, H3 and H4, in *B. stoltei* using the domain models from Histone DB (v2.0) (Fig. S11). We found eleven putative H2A, five H2B, eleven H3 and five H4 histone proteins. Histone H2A forms dimers with histone H2B and histone H3 forms dimers with histone H4 (136). The H2B and H4 histones are known to be more conserved in comparison to H2A and H3 across several eukaryotic lineages (136). The trend of greater diversity in homologs of H2A and H3 in other eukaryotic lineages is also preserved in *Blepharisma*, where there are twice as many H2A homologs as those of H2B and almost twice as many H3 homologs as those of H4.

Unlike *Paramecium tetraurelia* and *Tetrahymena thermophila*, which have longer, divergent histone H3's proposed to be centromeric (137), i.e, CenH3, we did not observe such histones in *Blepharisma*. Both of the longer *Blepharisma* histone H3's have unusual N-terminal domains (VIT and VWA_3, PF08487 and PF13768, respectively). In the PFAM database (34.0) the pairing of these two domains exclusively without any other domains represents the most common domain architectures for both. Searches of UniProt reveal that this domain pair is common in eukaryotes and bacteria, but it is not known what role they play in combination (138). Furthermore, the pair of proteins with the VIT-VWA_3 domain pair represent the most weakly expressed histone H3 domain-containing proteins in *Blepharisma*.

Since substantial upregulation of certain histone variants occurs during development in both *Oxytricha* and *Stylonychia*, including during the period of genome editing (115, 135, 139), we examined the patterns of expression during *Blepharisma* development. Among the *Blepharisma* histones, certain candidates of three of the core histones H2A, H2B and H3 are constitutively expressed at similar levels throughout the cycle of sexual reproduction, while others are upregulated at timepoints corresponding to different stages of meiosis (6 h and 14 h timepoints) and also subsequently during new MAC development. The patterns of expression observed suggest that even the *Blepharisma* genome encodes variants that are likely to have a range of different functions, including in genome editing and likely also during DNA amplification in the developing new MAC. Histone H4, in contrast, appears to be expressed at relatively similar levels throughout conjugation. This constitutive expression of histone H4 is a characteristic shared among eukaryotes, which lack functional variants due their highly conserved constitution, a trait suggested to be favored by the greater necessity of this histone to maintain several protein-protein contacts with the other three histones (136).

PiggyBac homologs in other heterotrichs, but not the oligohymenophorean, *Ichthyophthirius multifiliis*

PiggyMac homologs are also present in other heterotrich ciliates but have not yet been described because of genome assembly or annotation challenges. Using BPgm as a query sequence, we found convincing homologs containing the conserved catalytic DDD-motif in a genome assembly of the heterotrichous ciliate *Condylostoma magnum* (TBLASTN e-value 2e-24 to 2e-37). All the *C. magnum* PiggyMac homologs have a complete DDD-catalytic triad. While we failed to detect the DDE_Tnp_1_7 domain in predicted genes of the heterotrich *Stentor coeruleus*, we detected relatively weak adjacent TBLASTN matches split across two frames in its draft MAC genome (e-value 7e-15; SteCoe_contig_741 positions 6558-5475). After joining ORFs corresponding to this region and translating them, we obtained a more convincing DDE_Tnp_1_7 match with HMMER3 (e-value 2e-24). This either corresponds to a pseudogene or a poorly assembled genomic region.

In addition, we searched for PiggyMac homologs in the MAC genome of the pathogenic oligohymenophorean ciliate *Ichthyophthirius multifiliis* (140). TBLASTN searches using the *T. thermophila* Tpb2 as a query returned no hits. A HMMER search using hmmscan with a six-frame translation of the *I. multifiliis* MAC genome against the PFAM-A database also did not return any matches with independent E-values (i-E-value) less than 1. We note that based on BUSCO analyses the *I. multifiliis* genome appears to be less complete than other ciliates we examined (Fig. S2A). So, a better genome assembly will be needed to investigate the possibility that PiggyBac homologs are encoded elsewhere in this MAC genome.

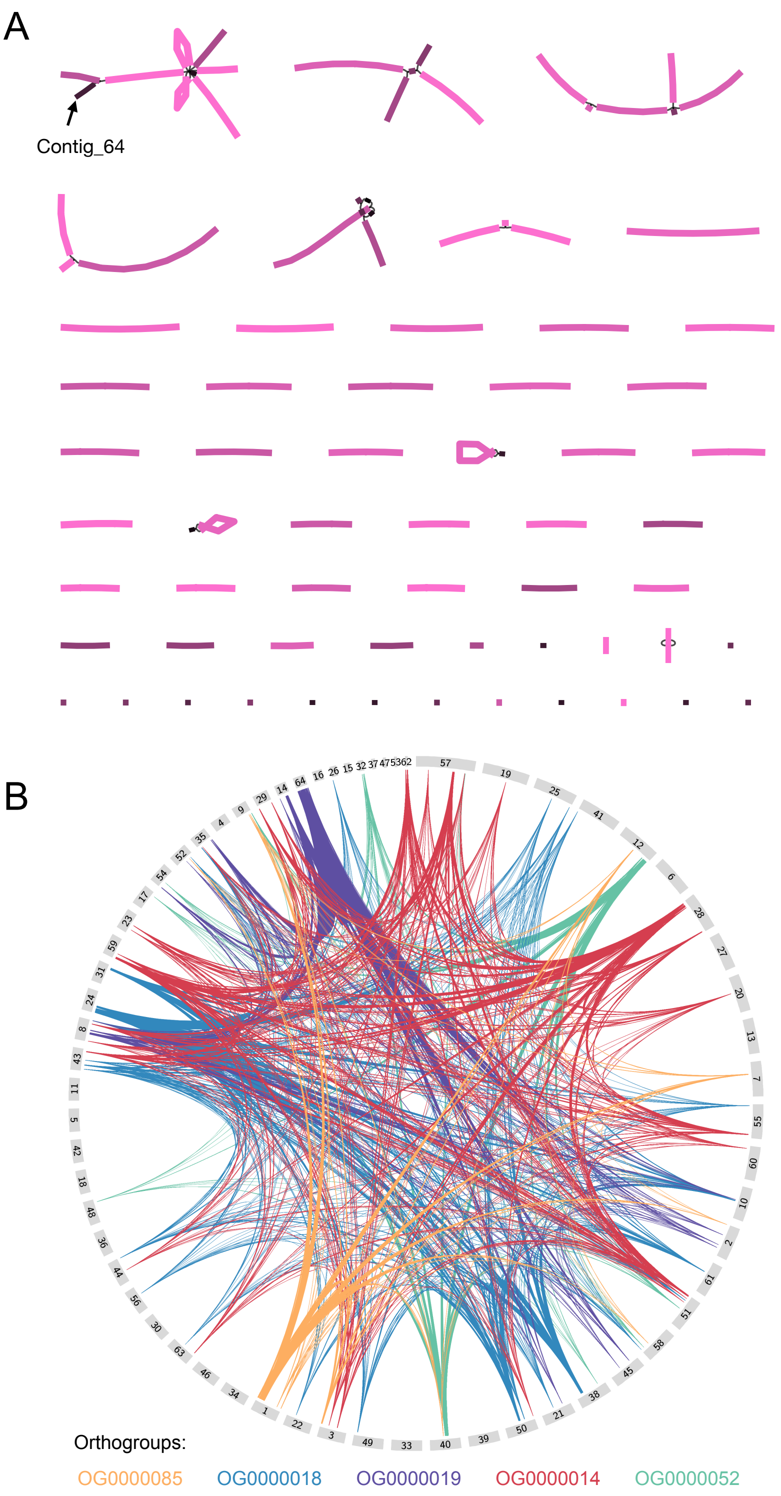

Fig. S1. *B. stoltei* ATCC 30299 MAC genome orthogroups and assembly graph. (*A*) Bandage (141) representation of Flye 2.8.1 assembly graph. Edges corresponding to contigs are colored by coverage (brightest pink = 160×, black=0×). (*B*) Clustered orthogroups (Data S2) in the *B. stoltei* MAC genome.

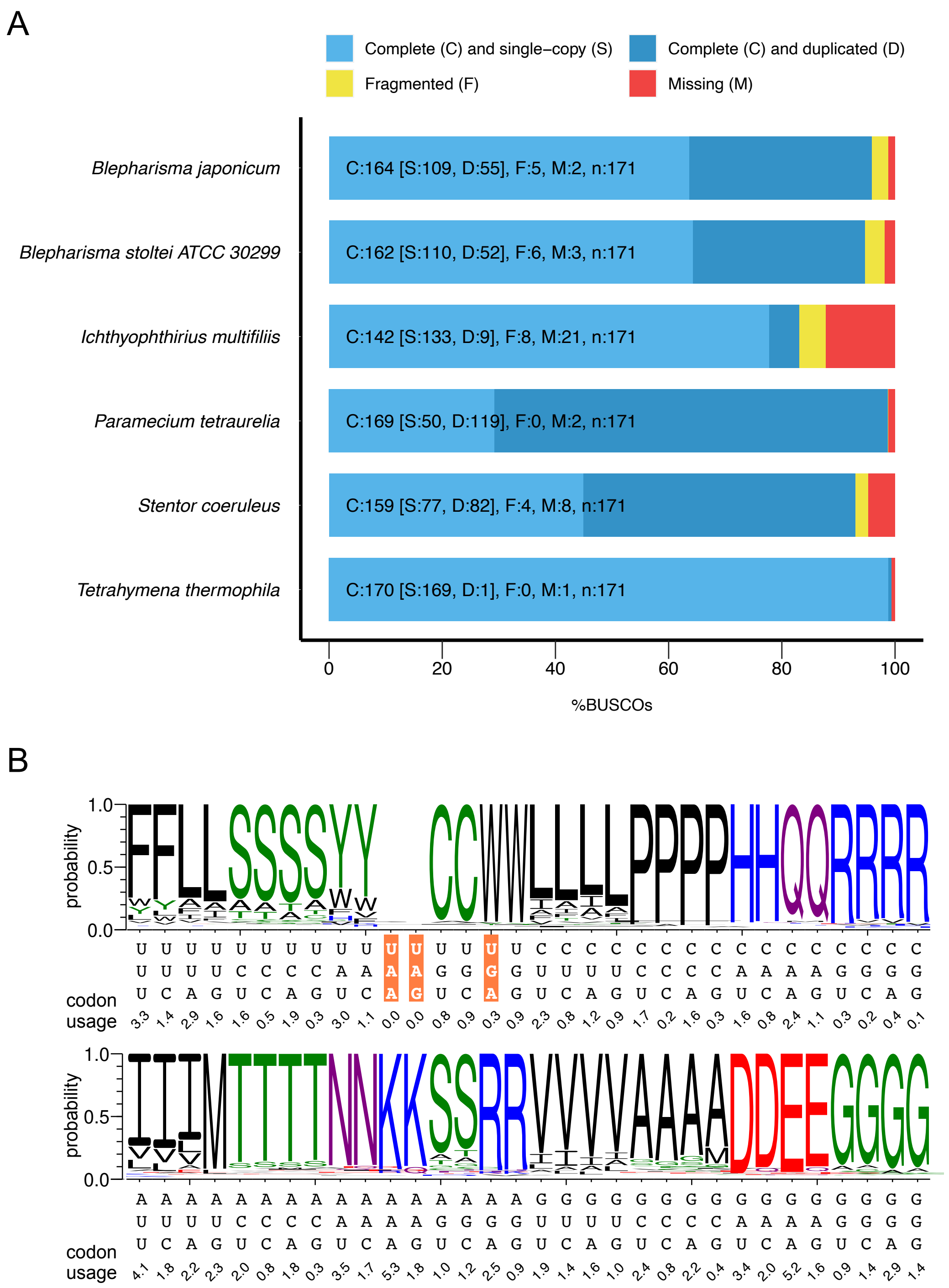

Fig. S2. Analysis of assembly completeness and genetic code. (*A*) Completeness of the *B. stoltei* ATCC 30299 MAC assembly was estimated by the percentage of BUSCOs found in the assembly with reference to the OrthoDB v10 alveolate database (142). The nature of the ortholog matches is indicated by characters followed by counts: C (complete orthologs) - light blue, D (duplicated orthologs) - dark blue, F (fragmented orthologs) - yellow and M (missing orthologs) - red. (*B*) PORC genetic code prediction for *B. stoltei* ATCC 30299 MAC genome; codons that are stops in the standard genetic code are highlighted in orange.

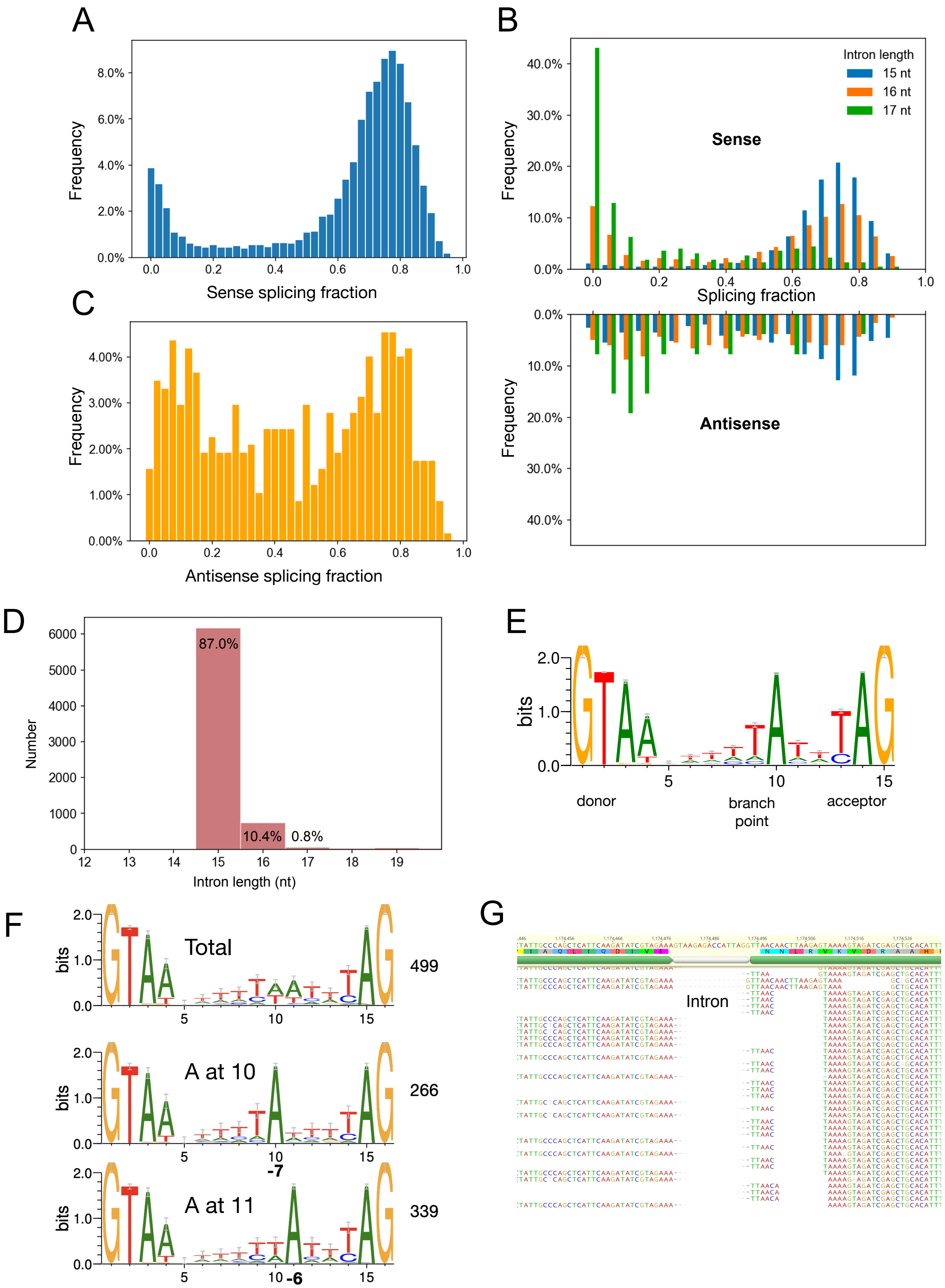

Fig. S3. Intron splicing. (*A*) Distribution of intron splicing fraction of candidate sense introns in the *B. stoltei* MAC genome. (B) Distribution of intron splicing fractions of introns according to intron lengths. (*C*) Distribution of intron splicing fraction of candidate antisense introns. (*D*) Distribution of intron lengths from predicted genes. (*E*) Sequence logos for 15 bp introns (splicing frequency > 0.5). (*F*) Sequence logos for all predicted 16 nt introns, and 16 nt introns with "A" at either position

-7 or -6 (counting from the 3'-most base which is -1). The number of introns underlying the logos are indicated to the right. (*G*) Distribution of intron splicing fractions of introns according to intron lengths. (*H*) Sample of RNA-seq reads mapped to a GT-GG intron from gene BSTOLATCC_MAC21551 (Contig_57.g761). Translation in alternative reading frames downstream of the predicted intron leads to premature stop codons soon after the intron.

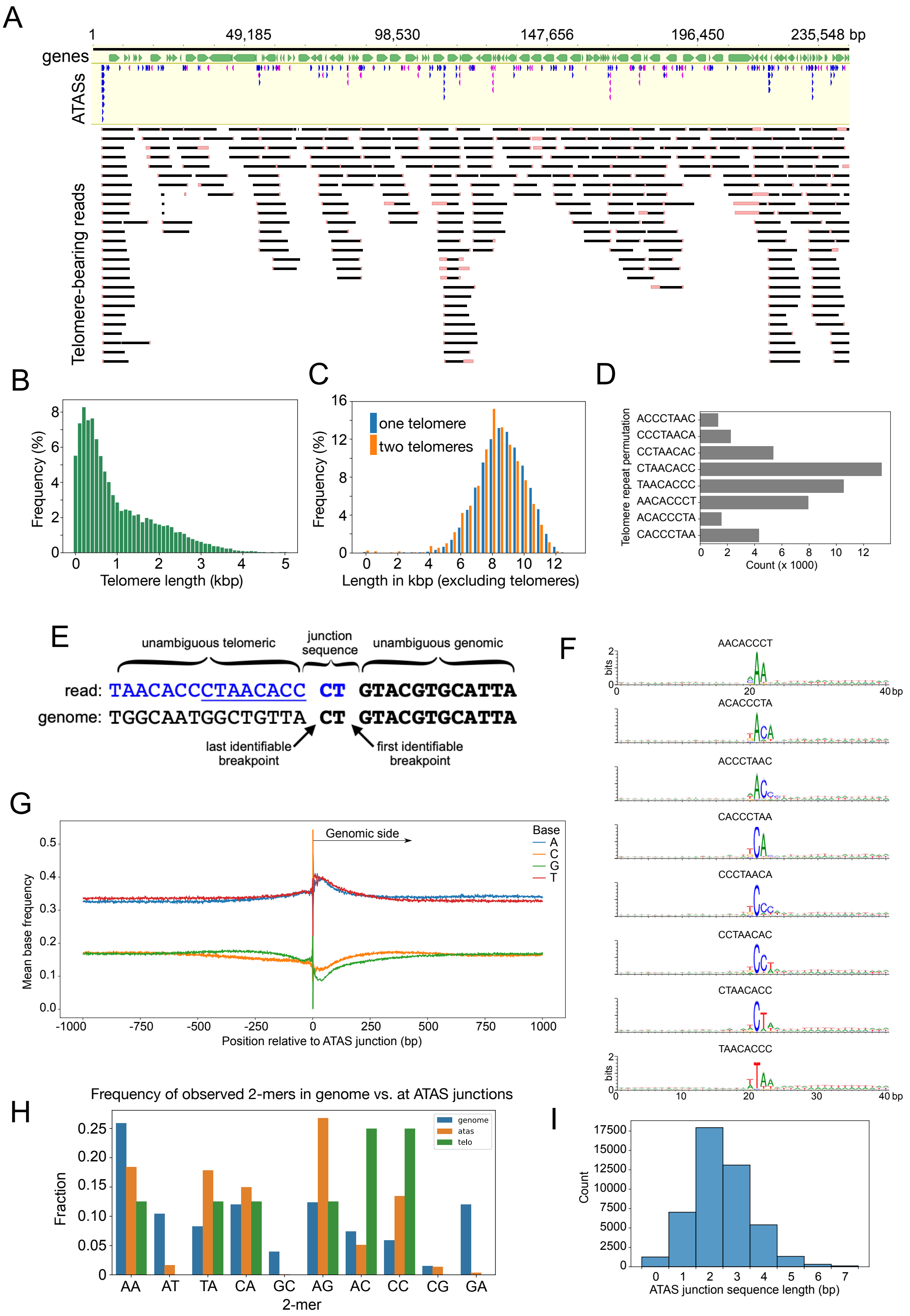

Fig. S4. Properties of minichromosomes, telomeres, and alternative telomere addition sites. (*A*) Mapping of a subset telomere-containing HiFi reads to a *B. stoltei* MAC genome contig region, with alternative telomere addition sites (ATASs) shown by blue (5') or mauve (3') arrows. Pink bars at read ends indicate soft-masking, typically of telomeric repeats. (*B*) Length distribution of

telomeres of telomere-bearing HiFi reads. (*C*) Length distribution of HiFi reads delimited by telomeres. (*D*) Counts of each telomere repeat permutation at ATAS junctions (last identifiable breakpoint). (*E*) Diagram of a telomere-bearing read mapped onto genome reference at an ATAS. Sequence which is ambiguously chromosomal or telomeric is “junction sequence”; junction coordinate which maximizes telomere repeat length on the read is the “first identifiable breakpoint”; the coordinate maximizing alignment length to reference is the “last identifiable breakpoint”. The last telomeric unit permutation at the last identifiable breakpoint is underlined (length 8 bp). (*F*) Sequence logos of chromosomal sequence at ATAS junctions, sorted by which permutation of the telomeric repeat is present (plot labels). Logos are aligned to the “last identifiable breakpoint” between positions 20 and 21; telomeric repeats on telomere-bearing reads begin to the left of the breakpoint. (*G*) Mean base frequencies in +/- 1 kbp flanking ATAS junctions. (*H*) Frequencies of 2-mers in whole genome (blue), in telomeres (green), and at ATAS junctions (chromosomal side after last identifiable breakpoint, orange). (*I*) Histogram of junction sequence lengths for ATASs in *B. stoltei*.

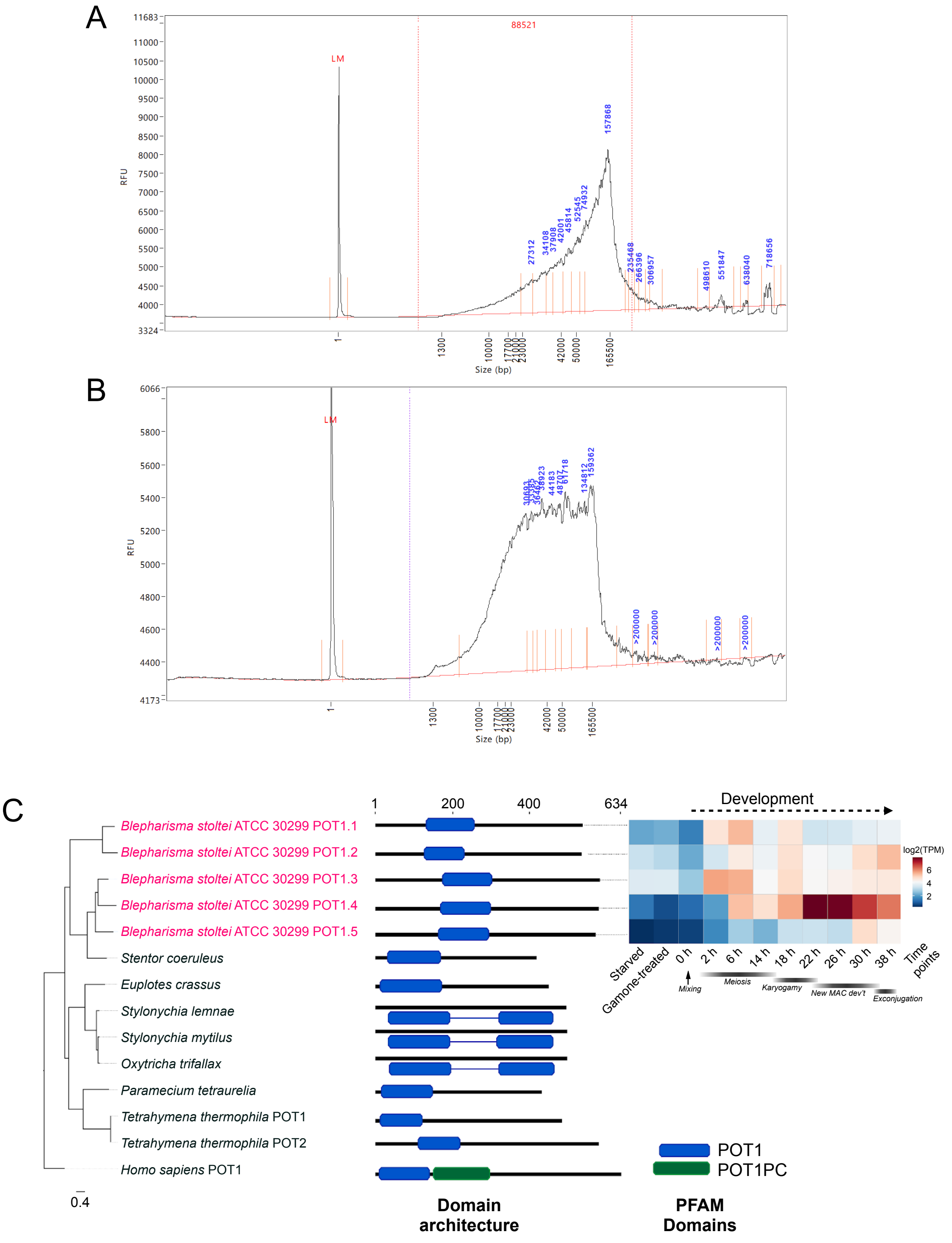

Fig. S5. Femto Pulse analyses of *B. stoltei* MAC DNA and POT1 phylogeny. (*A*) Length distribution of input MAC DNA sizes prior to fragmentation and library preparation (Femto Pulse; LM = lower maker) - replicate 1. RFU=relative fluorescent units. (*B*) Length distribution of input MAC DNA sizes prior to fragmentation and library preparation (Femto Pulse; LM = lower maker) - replicate 2. (*C*) POT1 paralog phylogeny, PFAM domain architecture, and gene expression in *Blepharisma.* Diagram elements as described in Fig. 5B.

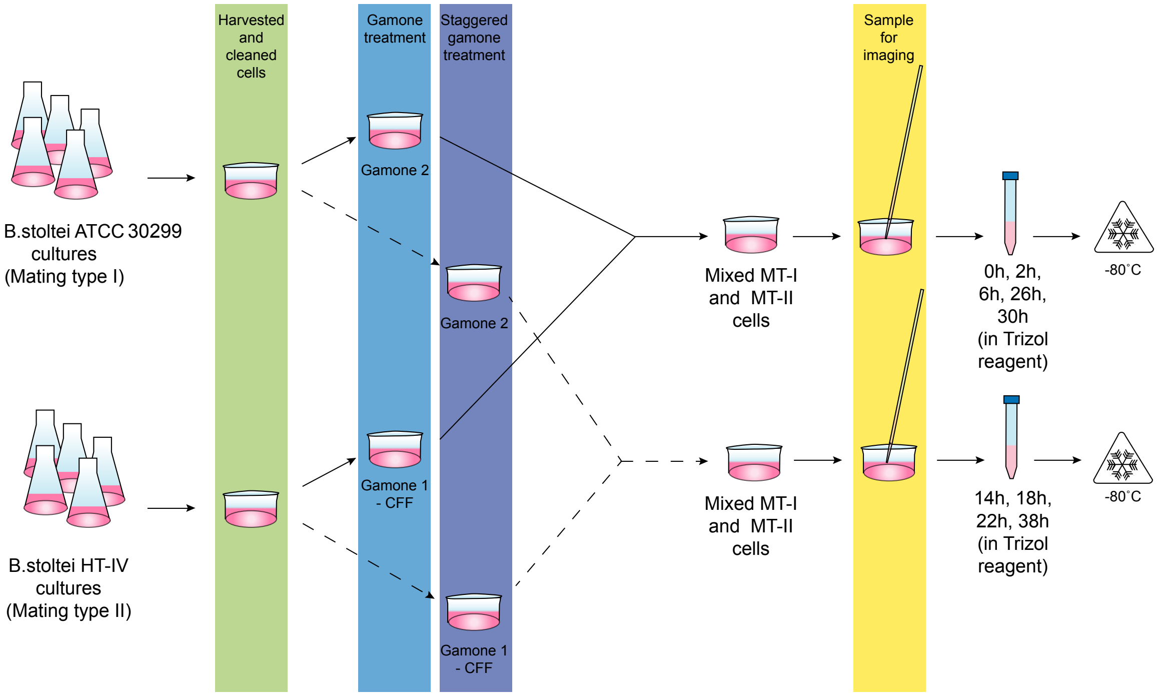

Fig. S6. Experimental approach for conjugation RNA-seq time series. Complementary mating type strains of *Blepharisma stoltei* were harvested and cleaned by starving overnight. The cleaned cultures were treated in a time-staggered format, with gamones of the complementary mating type, where gamone 2 was a solution of the synthetic gamone 2 calcium salt and gamone 1 was provided as the cell-free fluid (CFF) harvested from mating-type I cells. Two sets of time-staggered gamone-treated cultures were used for the time series. Set I, indicated by the solid line, was mixed and used to observe and collect samples at 0 hours, 2 hours, 6 hours, 26 hours and 30 hours after mixing. Set II, indicated by the dashed lines, was mixed and used to observe and collect samples at 14 hours, 18 hours, 22 hours and 38 hours after mixing. Test tubes indicate Trizol samples prepared for RNA-extraction which were stored at -80 ˚C before processing. Cells collected for imaging were obtained shortly before the remainder were transferred into Trizol.

A Dicers, Dicer-likes and Dicer-derivatives

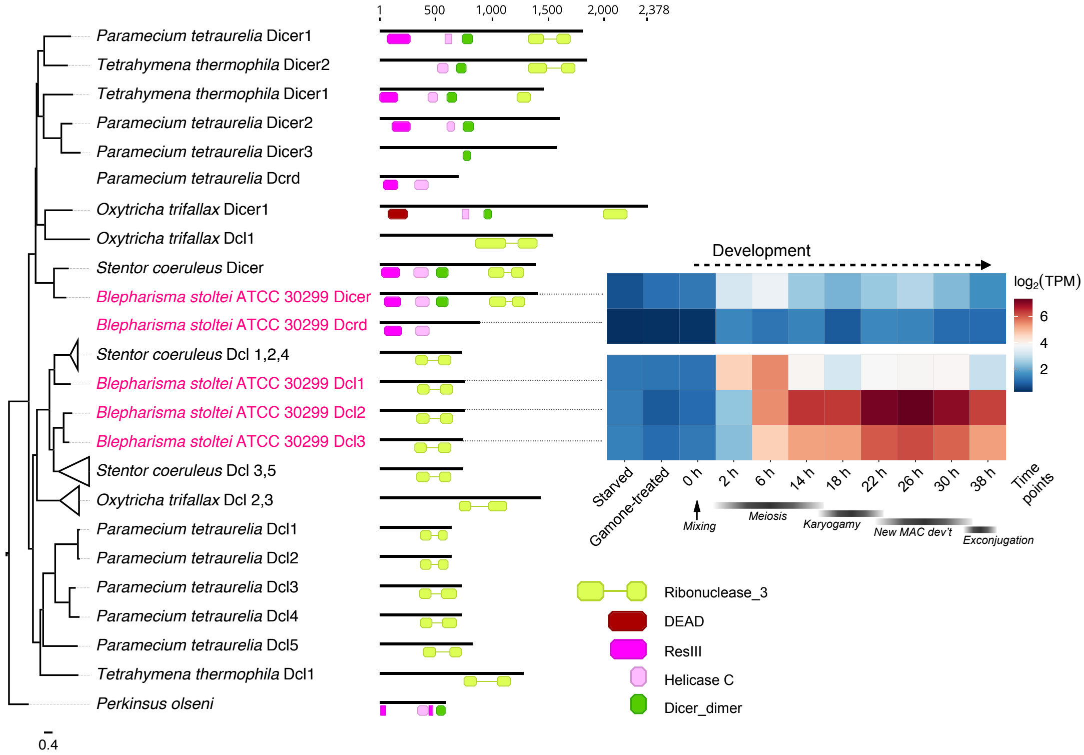

B Piwi proteins

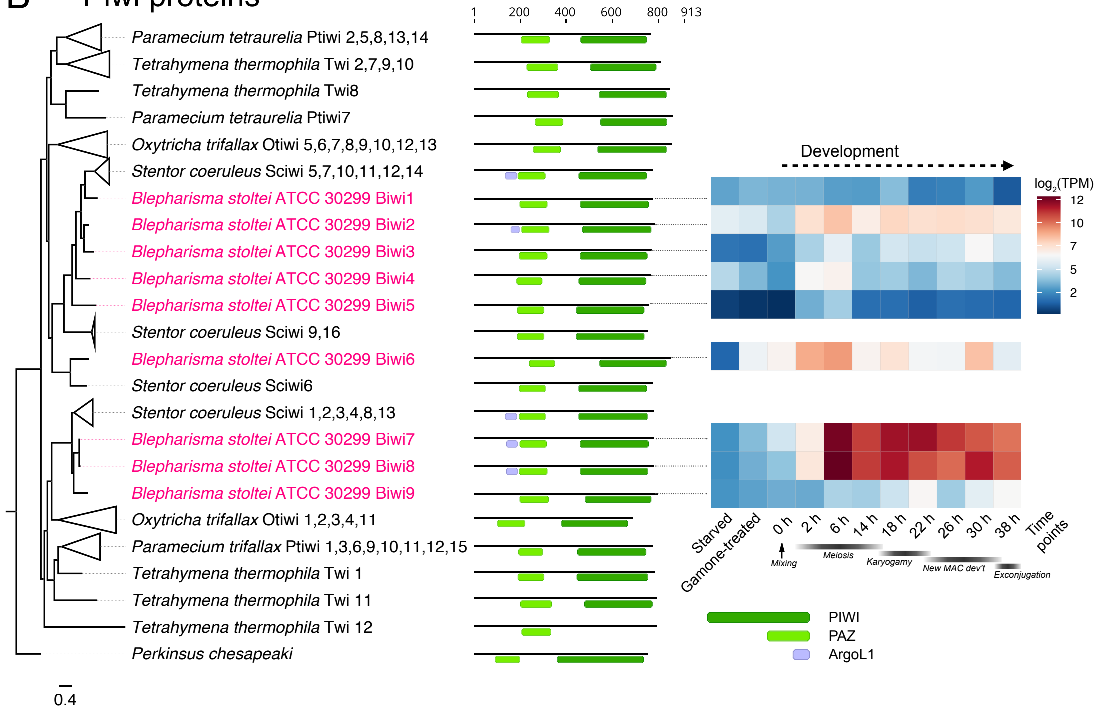

Fig. S7. Small RNA-related proteins in *Blepharisma stoltei* ATCC 30299. (*A*) ResIII, Helicase_c and Ribonuclease_3 domain phylogeny with PFAM domain architecture and gene expression heatmap for *Blepharisma*. (*B*) PIWI domain phylogeny with PFAM domain architecture and gene expression heatmap for *Blepharisma*

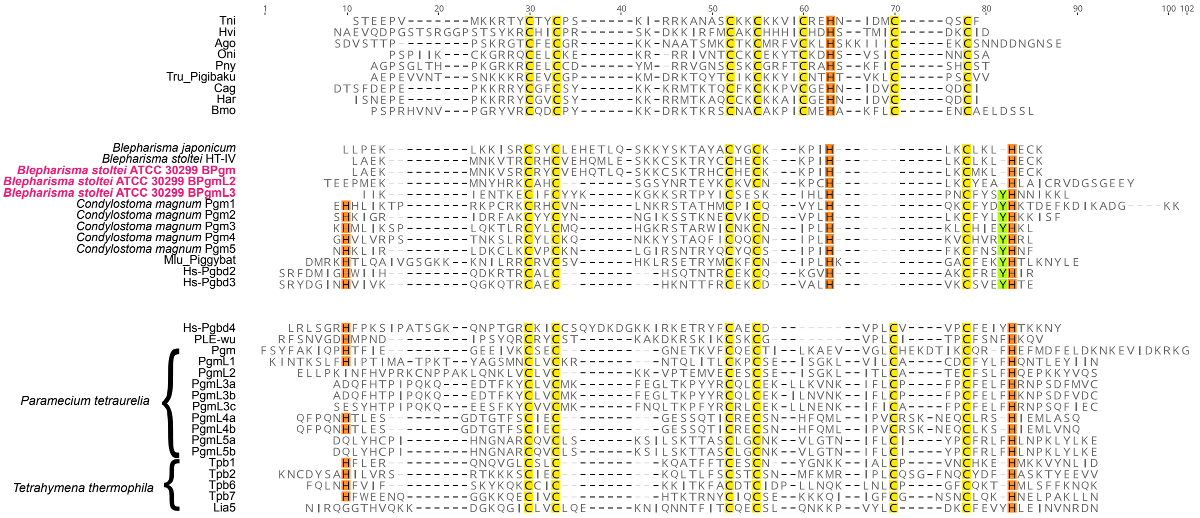

Fig. S8. Cysteine-rich domains of PiggyBac homologs. PBLE transposases: Ago (*Aphis gossypii*); Bmo (*Bombyx mori*); Cag (*Ctenoplusia agnata*); Har (*Helicoverpa armigera*); Hvi (*Heliothis virescens*); PB-Tni (*Trichoplusia ni*); Mlu (PiggyBat from *Myotis lucifugus*); PLE-wu (*Spodoptera frugiperda*). Domesticated PGBD transposases: Oni (*Oreochromis niloticus*); Pny (*Pundamilia nyererei*); Lia5, Tpb1, Tpb2, Tpb6 and Tpb7 (*Tetrahymena thermophila*); Pgm, PgmL1, PgmL2, PgmL3a/b/c, PgmL4a/b, PgmL5a/b (*Paramecium tetraurelia*); Tru (*Takifugu rubripes*); Pgbd2, Pgbd3 and Pgbd4 (*Homo sapiens*).

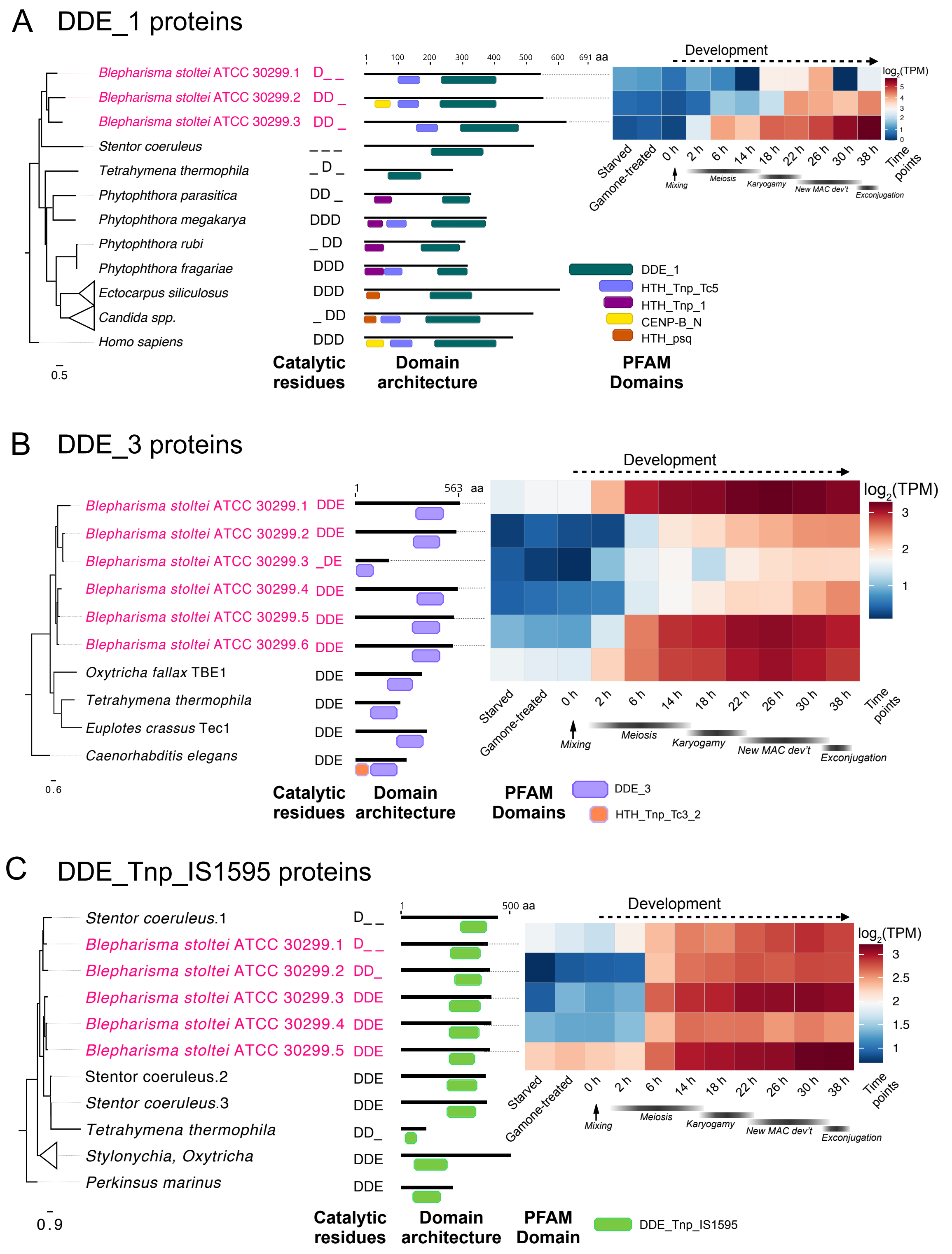

Fig. S9. DDE_1, DDE_3 and DDE_Tnp_IS1595 domain-containing proteins in *Blepharisma stoltei* ATCC 30299. (*A*) DDE_1 domain phylogeny with PFAM domain architecture and gene expression heatmap for *Blepharisma*. (*B*) DDE_3 domain phylogeny with PFAM domain architecture and gene expression heatmap for *Blepharisma*. (*C*) DDE_Tnp_IS1595 domain phylogeny with PFAM domain architecture and gene expression heatmap for *Blepharisma*.

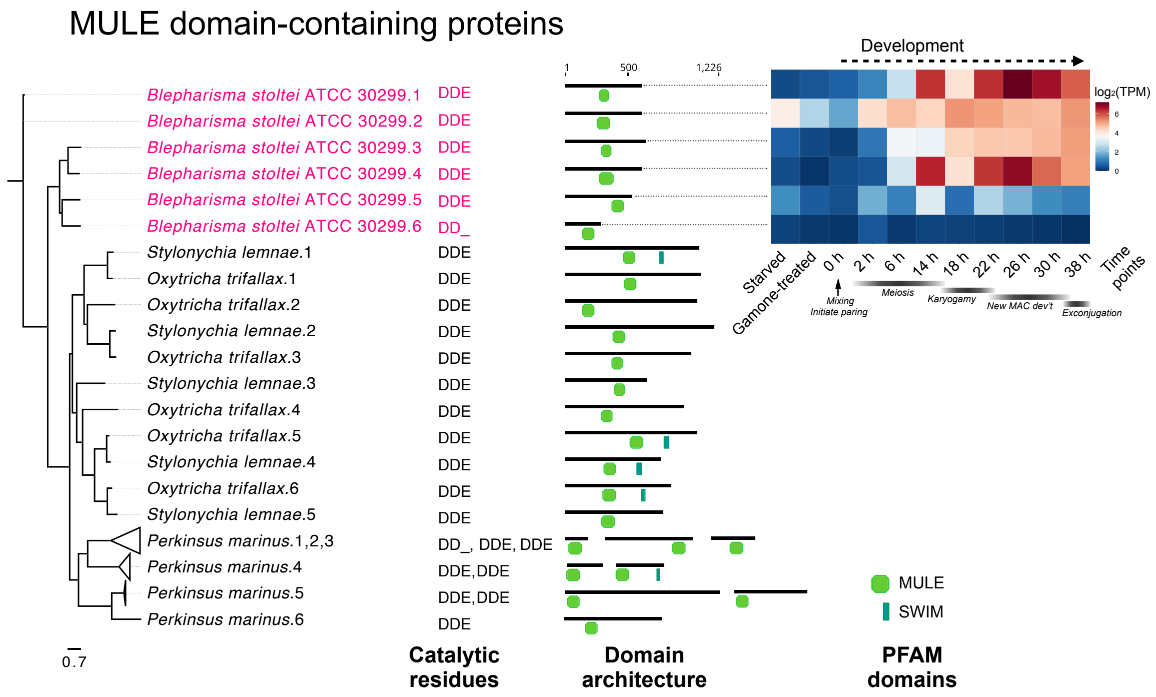

Fig. S10. MULE domain transposases in *Blepharisma stoltei* ATCC 30299. MULE domain phylogeny with PFAM domain architecture and gene expression heatmap.

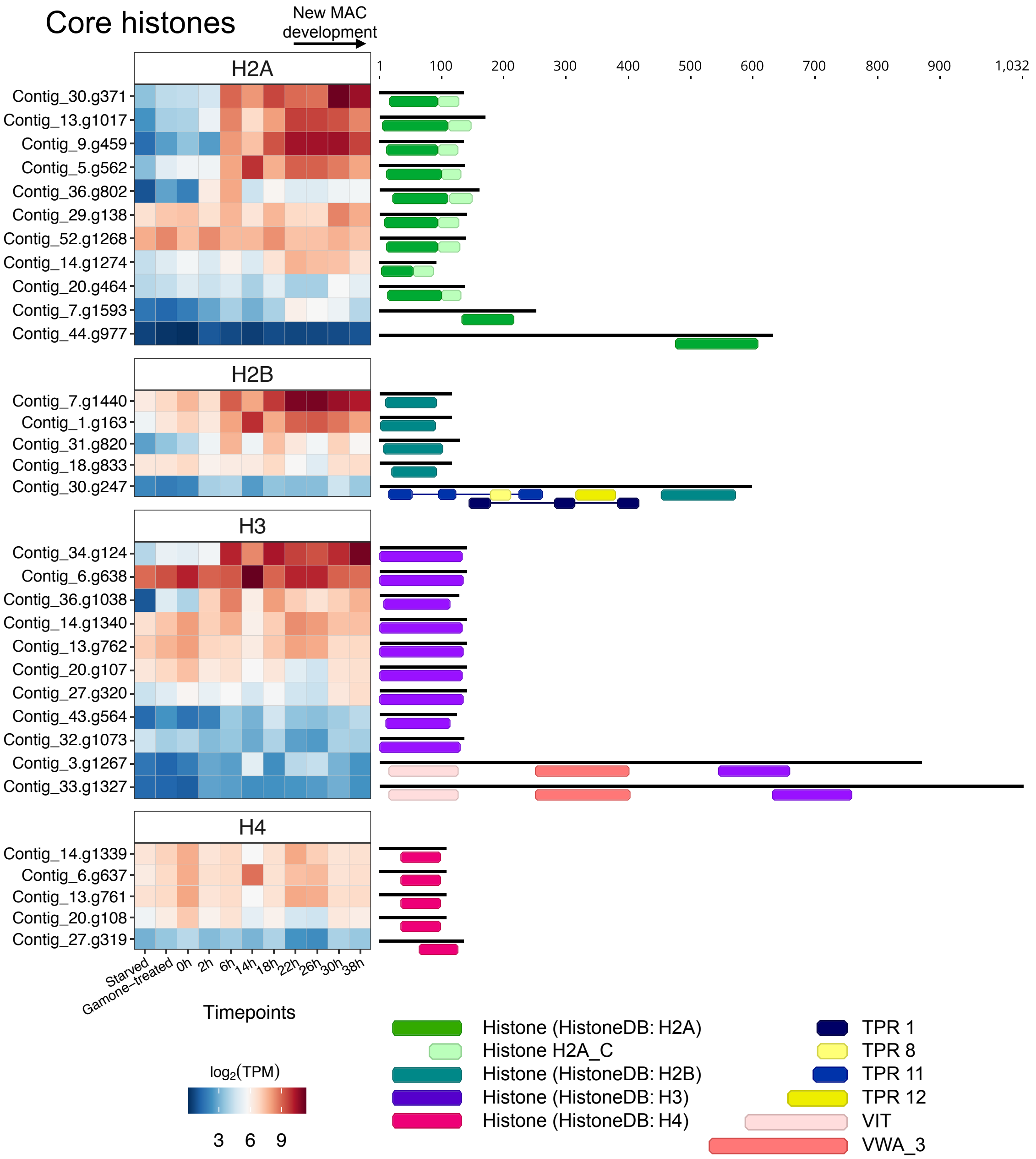

Fig. S11. Histones and histone-domain-containing proteins in *Blepharisma stoltei* ATCC 30299. Gene expression heatmaps are shown as in previous figures, are clustered according to major histone type as classified using HistoneDB domain models. Domains from PFAM and HistoneDB are shown to the right.

Table S1. Citations for genome properties from Fig. 2.

Species	Genome size (Mb)	Genome architecture	Genes (zygosity)	Codon reassignments
Blepharisma stoltei	41	Minichromosomes	25,726 (n)	UGA -> W
Stentor coeruleus	77[2]	?	31,426 (?)[2]	Standard genetic code[2]
Paramecium tetraurelia	72[3]	Chromosomes[3, 4]	39,642 (n)[3]	UAA, UAG -> Q[1]
Tetrahymena thermophila	103[5]	Chromosomes[6]	26,258 (n)[5]	UAA, UAG -> Q[1]
Euplotes octocarinatus	88[7]	Nanochromosomes[8]	29,076 (?)[7]	UGA -> C[9]
Stylonychia lemnae	52[10]	Nanochromosomes[10]	15,102 (2n)[10]	UAA, UAG -> Q[1]
Oxytricha trifallax	50[11]	Nanochromosomes[11]	18,400 (2n)[11]	UAA, UAG -> Q[1]
Perkinsus olseni	63[12]	Chromosomes[12]	17,342 (4n)[12]	Standard genetic code[12]

1. Swart, E. C., Serra, V., Petroni, G. & Nowacki, M. Genetic Codes with No Dedicated Stop Codon: Context-Dependent Translation Termination. *Cell* 166, 691–702 (2016).
2. Slabodnick, M. M. *et al.* The Macronuclear Genome of Stentor coeruleus Reveals Tiny Introns in a Giant Cell. *Curr. Biol.* 27, 569–575 (2017).
3. Aury, J.-M. *et al.* Global trends of whole-genome duplications revealed by the ciliate Paramecium tetraurelia. *Nature* 444, 171–178 (2006).
4. Duret, L. *et al.* Analysis of sequence variability in the macronuclear DNA of Paramecium tetraurelia: A somatic view of the germline. *Genome Res.* 18, 585–596 (2008).
5. Sheng, Y. *et al.* The completed macronuclear genome of a model ciliate Tetrahymena thermophila and its application in genome scrambling and copy number analyses. *Sci. China Life Sci.* 63, 1534–1542 (2020).
6. Eisen, J. A. *et al.* Macronuclear genome sequence of the ciliate Tetrahymena thermophila, a model eukaryote. *PLoS Biol.* 4, 1620–1642 (2006).
7. Wang, R. lin, Miao, W., Wang, W., Xiong, J. & Liang, A. hua. EOGD: The Euplotes octocarinatus genome database. *BMC Genomics* 19, 1–6 (2018).
8. Ghosh, S., Jaraczewski, J. W., Klobutcher, L. A. & Jahn, C. L. Characterization of transcription initiation, translation initiation, and poly(A) addition sites in the gene-sized macronuclear DNA molecules of Euplotes. *Nucleic Acids Res.* 22, 214–221 (1994).
9. Meyer, F. *et al.* UGA is translated as cysteine in pheromone 3 of Euplotes octocarinatus. *Proc. Natl. Acad. Sci. U. S. A.* 88, 3758–3761 (1991).
10. Aeschlimann, S. H. *et al.* The Draft Assembly of the Radically Organized Stylonychia lemnae Macronuclear Genome. *Genome Biol. Evol.* 6, 1707–1723 (2014).
11. Swart, E. C. *et al.* The Oxytricha trifallax Macronuclear Genome: A Complex Eukaryotic Genome with 16,000 Tiny Chromosomes. *PLoS Biol.* 11, e1001473 (2013).
12. Bogema, D. R. *et al.* Draft genomes of Perkinsus olseni and Perkinsus chesapeaki reveal polyploidy and regional differences in heterozygosity. *Genomics* 113, 677–688 (2021)

Table S2. Comparison of *Blepharisma stoltei* ATCC 30299 MAC genome assemblies.

**Assembly**	**Flye (v2.7) Replicate 1**	**Flye (v2.7) Replicate 2**	**Flye (v2.7) Combined**	**Flye (v2.8) Combined**	**Final assembly**
Contigs	89	86	74	72	64 (excluding mitogenome)
Mean coverage (from flye.log)	76	70	145	145	NA
%GC	33.3	33.3	33.4	32.9	33.6
Longest contig (bp)	2,036,921	1,188,116	1,541,963	1,608,201	1,514,878
Assembly size (bp)	42,701,284	43,066,385	43,062,848	42,982,242	41,464,486
N50	738,771	757,357	799,426	817,639	795,340
Two telomeres	38	37	36	16	64
One telomere	36	36	25	32	0
Zero telomeres	15	13	13	24	0

Table S3. Top 100 genes in *Blepharisma stoltei* ATCC 30299 ranked according to absolute expression at 26 hours.

***B. stoltei* ATCC 30299 MAC gene expression** (only genes with PFAM annotations; domain multiplicitly indicated by "x" and a number; blue = 0-1 RPKM; cyan = 1-10 RPKM; 10-100 RPKM = yellow; 100-1000 RPKM = orange; 1000-10000 RPKM = red)

	ENA accession	Gene ID	Length (CDS in bp)	Starved	Gamone-treated	0 h	2 h	6 h	14 h	18 h	22 h	26 h	30 h	38 h	26h/average(starved +gamone+0h)	Transposase	DNA repair	Chromatin	Transcription	Small RNAs	Translation	PFAM Domains	eggNOG gene name	eggNOG description	Pannzer gene name	Pannzer description	Pannzer PPV
1	BSTOLATCC_MAC13665	Contig_40.g581	1362	7712.4	10287.9	9655.0	9848.3	7732.0	9765.3	8089.2	8084.9	10403.2	8266.2	14239.1	1.1							GTP_EFTU x1 GTP_EFTU_D3 x1 (	TEF1	regulation of response to stimulus	NA	Elongation factor 1-alpha	0.46
2	BSTOLATCC_MAC22028	Contig_58.g1224	1167	3975.0	4211.8	4389.2	6931.6	5651.7	6554.9	3683.2	6009.0	6740.9	3880.1	5676.0	1.6							ubiquitin x5 Rad60-SLD x5		ubiquitin	NA	Polyubiquitin	0.62
3	BSTOLATCC_MAC7206	Contig_25.g669	2508	6164.1	6433.6	5759.2	5345.9	3753.0	4862.5	4374.3	3794.7	4592.3	3577.8	5261.9	0.8							GTP_EFTU x1 EFG_IV x1 EFG_C		Elongation factor G, domain IV fa	NA	Elongation factor 2	0.59
4	BSTOLATCC_MAC8818	Contig_28.g906	1335	7231.4	8506.6	10323.3	8424.5	5922.1	4983.5	3383.7	3704.3	4375.8	3077.5	3404.1	0.5							Tubulin x1 Tubulin_C x1 Misat_Tub		Tubulin is the major constituent of	NA	Tubulin beta chain	0.51
5	BSTOLATCC_MAC9322	Contig_29.g167	975	2630.7	2828.7	2256.3	2164.1	1992.5	2427.7	2399.3	2720.4	4361.1	2932.7	3084.9	1.7							Ldh_1_C x1 Ldh_1_N x1		Malate dehydrogenase	NA	Malate dehydrogenase	0.44
6	BSTOLATCC_MAC11989	Contig_36.g807	2115	215.8	242.1	751.0	2684.2	2380.5	1191.9	775.8	1694.3	3605.0	1583.4	901.0	8.9							HSP90 x1 HATPase_c x1 HATPase	HSP90	Heat shock protein	HSP90	Heat shock protein 90	0.56
7	BSTOLATCC_MAC235	Contig_1.g235	510	5822.9	4983.4	5291.7	4067.5	4404.2	3923.0	5056.5	4119.8	3537.3	4512.3	5802.0	0.7							zf-C2H2 x1 zf-H2C2_2 x1 zf-Di19 x		Zinc finger, C2H2 type	NA	NA	NA
8	BSTOLATCC_MAC19444	Contig_52.g1287	1131	3118.4	4433.0	4259.3	3163.3	2784.2	2687.8	2623.6	2450.0	3346.1	2845.8	3395.0	0.8							Actin x1Actin x1Actin x1Actin x1		Actin	NA	Actin	0.6
9	BSTOLATCC_MAC20266	Contig_55.g708	1059	5877.8	8250.6	4377.8	3918.4	2937.8	3491.6	3508.9	2749.1	3051.9	3228.4	3229.2	0.5							Peptidase_C1 x1 Inhibitor_I29 x1 F		cysteine-type endopeptidase activ	NA	Cysteine protease-1	0.67
10	BSTOLATCC_MAC7218	Contig_25.g681	2007	378.4	539.3	1538.5	2037.1	3363.6	1283.8	1388.8	1690.3	2970.0	2671.0	1983.5	3.6							HSP70 x1 MreB_Mbl x1	HSP70	heat shock protein 70	HSP70	Heat shock protein 70	0.55
11	BSTOLATCC_MAC20471	Contig_56.g905	1431	5214.7	5813.6	3727.6	3824.5	2628.6	3179.9	2384.1	2675.5	2917.4	2717.3	3373.1	0.6							AdoHcyase x1 AdoHcyase_NAD x1	AHCYL1	adenosylhomocysteinase activity	NA	Adenosylhomocysteinase	0.53
12	BSTOLATCC_MAC19601	Contig_54.g48	516	3534.6	4007.6	4767.6	2916.7	2869.5	2742.2	3109.5	2614.4	2814.3	3242.1	3465.8	0.7							TCTP x1TCTP x1TCTP x1TCTP x1	TPT1	Translationally controlled tumour p	NA	TCTP domain-containing protein (F	0.51
13	BSTOLATCC_MAC17155	Contig_48.g755	1896	7.1	12.3	9.7	60.2	3458.6	4149.6	2813.3	2326.9	2799.4	2446.1	1305.3	288.1							DEAD x1 zf-CCHC x4 Helicase_C x	DBP2	Belongs to the DEAD box helicase	DDX43	RNA helicase	0.41
14	BSTOLATCC_MAC25659	Contig_8.g281	1032	4697.5	4933.8	8139.4	4222.1	3275.1	4421.9	3634.2	2659.0	2744.1	2451.2	2259.2	0.5							Gp_dh_C x1 Gp_dh_N x1		Glyceraldehyde-3-phosphate deh	NA	Glyceraldehyde-3-phosphate dehy	0.45
15	BSTOLATCC_MAC5403	Contig_20.g450	513	3405.3	4181.7	3231.0	2230.0	3065.7	3322.0	3606.4	3107.9	2744.0	3524.0	4678.4	0.8							Pro_isomerase x1	-CYP 1.00	PPIases accelerate the folding of	NA	Peptidyl-prolyl cis-trans isomerase	0.43
16	BSTOLATCC_MAC4350	Contig_19.g1647	1056	4114.4	4806.8	3543.2	3053.1	2561.0	2509.8	2578.3	2527.8	2590.2	2066.0	2990.5	0.6							Peptidase_C1 x1 Inhibitor_I29 x1 F		cysteine-type endopeptidase activ	NA	Fruit bromelain	0.44
17	BSTOLATCC_MAC23581	Contig_60.g663	1536	3206.6	3985.2	2509.7	2399.0	2001.0	2388.3	2276.9	2296.2	2485.5	2239.1	3020.2	0.8							Aldedh x1Aldedh x1Aldedh x1	ALDH7A1	Belongs to the aldehyde dehydro	NA	Aldedh domain-containing protein	0.43
18	BSTOLATCC_MAC21846	Contig_58.g1042	357	5819.0	4540.0	5027.4	2697.9	2582.4	3060.3	3909.7	2801.6	2388.8	3025.0	5348.3	0.5							Bac_globin x1Bac_globin x1	glbN	Bacterial-like globin	NA	NA	NA
19	BSTOLATCC_MAC23653	Contig_60.g735	714	3374.9	4042.0	3121.1	2392.3	2075.6	2330.7	2216.5	2130.3	2384.1	2240.2	2798.2	0.7							EF1_GNE x1 GST_C_2 x1 GST_C	CARS	EF-1 guanine nucleotide exchang	NA	Elongation factor 1-beta	0.3
20	BSTOLATCC_MAC5406	Contig_20.g453	2340	4.5	9.5	10.1	150.6	6059.2	4134.1	3321.1	2959.9	2282.3	1941.8	1102.0	283.3					◉		Piwi x1 PAZ x1 ArgoL1 x1		Piwi	NA	NA	NA
21	BSTOLATCC_MAC1451	Contig_12.g148	549	2.6	144.1	108.6	1282.1	6916.5	2971.2	2324.0	2534.4	2277.1	3006.5	1805.7	26.8							HMG_box x1 HMG_box_2 x1	NA	NA	NA	NA	NA
22	BSTOLATCC_MAC8375	Contig_27.g470	780	2039.5	2458.6	2955.8	2100.5	1981.4	1761.8	1555.8	1534.1	2248.0	1885.6	1392.6	0.9							Ribosomal_S3Ae x1	RPS3A	Belongs to the eukaryotic ribosom	RPS1	40S ribosomal protein S1	0.5
23	BSTOLATCC_MAC5529	Contig_20.g576	1032	2287.3	2280.0	2299.5	1384.4	1432.1	2541.4	2859.1	2191.6	2222.4	2210.9	2857.6	1.0							Gp_dh_C x1 Gp_dh_N x1		Glyceraldehyde-3-phosphate deh	NA	Glyceraldehyde-3-phosphate dehy	0.46
24	BSTOLATCC_MAC15966	Contig_45.g1208	636	3350.5	3972.7	1980.9	1900.7	1449.2	1978.8	2620.6	2282.5	2210.5	1920.2	2184.0	0.7							GST_C_3 x1 GST_C x1 GST_N x1		Glutathione S-transferase, N-termi	NA	Glutathione S-transferase	0.34
25	BSTOLATCC_MAC6598	Contig_23.g65	603	1847.7	1840.8	1671.4	2258.1	1863.9	1849.0	1386.2	1801.7	2179.3	1605.9	1528.1	1.2							Ribosomal_S8e x1		Ribosomal protein S8e	NSA2	Ribosome biogenesis protein NSA	0.73
26	BSTOLATCC_MAC13450	Contig_4.g162	636	1683.7	2134.8	1907.3	1483.4	1631.6	1818.6	2362.4	1813.8	2127.3	2423.0	2620.5	1.1							AhpC-TSA x1 Redoxin x1 1-cysPrx_		Tsa family	NA	peroxiredoxin-2	0.41
27	BSTOLATCC_MAC13519	Contig_4.g231	519	2440.5	2027.7	2274.5	1940.0	2106.9	2047.4	2374.6	2204.6	2058.3	2441.9	3145.9	0.9							zf-C2H2 x1 zf-Di19 x1		nucleic acid binding	NA	NA	NA
28	BSTOLATCC_MAC23948	Contig_61.g1025	906	5517.7	6461.1	3489.9	3076.5	2617.4	3574.9	4928.6	3425.4	2050.1	2395.9	5042.1	0.4							Peptidase_C1 x1Peptidase_C1 x1		Belongs to the peptidase C1 famil	NA	Cathepsin B	0.5
29	BSTOLATCC_MAC13751	Contig_40.g667	648	2414.2	2557.0	1914.2	1585.5	1315.9	1266.1	2249.7	1899.4	2046.8	2184.6	4272.1	0.9							Sod_Fe_C x1 Sod_Fe_N x1		oxidoreductase activity, acting on	NA	Superoxide dismutase	0.44
30	BSTOLATCC_MAC23158	Contig_6.g1156	1191	1923.5	2440.4	2780.1	2108.5	1717.2	1700.1	1194.0	1270.5	1982.4	1626.2	1135.3	0.8							Ribosomal_L3 x1Ribosomal_L3 x1	RPL3	60S ribosomal protein L3. Source	NA	60S ribosomal protein L3	0.56
31	BSTOLATCC_MAC7493	Contig_25.g956	1362	4720.6	5233.4	3802.4	3394.9	2205.8	2827.2	2306.9	2192.2	1798.9	1545.4	2192.1	0.4							Tubulin x1 Tubulin_C x1		Tubulin is the major constituent of	NA	Tubulin alpha chain	0.54
32	BSTOLATCC_MAC7844	Contig_25.g1307	1137	883.7	867.1	1279.8	1696.4	1262.5	1326.6	759.9	1171.8	1783.6	998.4	675.1	1.8							Alba x2Alba x2Alba x2Alba x2		Alba	NA	Alba domain-containing protein	0.37
33	BSTOLATCC_MAC6369	Contig_22.g1410	1047	1680.0	2138.4	1923.7	1804.7	1383.5	1303.4	966.8	1170.8	1756.3	1291.7	1170.4	0.9							Ribosomal_L4 x1 Ribos_L4_asso_		Ribosomal protein L4	NA	60S ribosomal protein L4	0.7
34	BSTOLATCC_MAC22535	Contig_59.g254	276	1560.1	1455.2	3302.0	1940.2	2831.7	1813.0	1763.9	1590.8	1752.8	2169.4	1685.2	0.8							EF-hand_1 x2 EF-hand_5 x2 EF-ha	NA	NA	NA	Calmodulin	0.5
35	BSTOLATCC_MAC642	Contig_10.g814	1080	2315.2	2854.8	2198.4	1762.5	1691.9	2057.9	2051.2	1826.7	1729.8	1733.5	1759.9	0.7							Glycolytic x1Glycolytic x1		fructose-bisphosphate aldolase	NA	Fructose-bisphosphate aldolase	0.5
36	BSTOLATCC_MAC21506	Contig_57.g716	672	3025.4	3615.0	2467.2	1640.4	1818.7	2011.7	2485.1	1920.8	1724.9	2053.6	3295.7	0.6							Ribosomal_S3_C x1 KH_2 x1	RpS3	positive regulation of DNA N-glyco	NA	KH type-2 domain-containing prote	0.53
37	BSTOLATCC_MAC21995	Contig_58.g1191	414	9.2	38.2	33.0	61.5	906.8	918.9	1414.2	1735.4	1722.8	2325.4	2117.3	64.3							Histone x1Histone x1Histone x1		protein heterodimerization activity	NA	Histone domain-containing protein	0.51
38	BSTOLATCC_MAC250	Contig_1.g250	798	3077.2	3710.4	2591.3	1810.0	1723.2	1976.1	1944.8	1793.8	1695.0	1746.4	2403.0	0.5							Ribosomal_S5 x1 Ribosomal_S5_C	RPS2	translation	NA	Small subunit ribosomal protein S2	0.56
39	BSTOLATCC_MAC8624	Contig_28.g712	1407	2824.7	3304.2	2192.3	2128.8	1631.8	1887.4	2089.1	1575.1	1657.4	1609.9	1823.8	0.6							EF1G x1 GST_C x1 GST_C_2 x1 (	EEF1G	translation elongation factor activi	NA	NA	NA
40	BSTOLATCC_MAC7002	Contig_24.g465	357	2129.9	2285.3	3106.6	1620.7	1509.6	1530.1	1803.2	1319.1	1580.3	1737.1	1821.8	0.6							SCP2 x1 Alkyl_sulf_C x1	SCP2D1	SCP-2 sterol transfer family	NA	SCP2 domain-containing protein	0.49
41	BSTOLATCC_MAC13544	Contig_4.g256	510	3181.1	2961.3	1910.5	1409.6	1399.5	2159.9	2191.9	2262.9	1547.7	1816.7	4250.7	0.6							zf-C2H2 x2 zf-Di19 x1	NA	NA	NA	NA	NA
42	BSTOLATCC_MAC3386	Contig_18.g685	747	2018.5	2245.7	1701.1	1532.1	1145.4	1435.8	1217.2	1278.2	1525.8	1168.3	1243.6	0.8							Ribosomal_L7Ae x1		Ribosomal protein L7Ae/L30e/S12	NA	60S ribosomal protein L8	0.39
43	BSTOLATCC_MAC14030	Contig_40.g946	546	0.4	33.4	17.0	721.8	4701.6	2241.2	3048.8	3099.9	1508.7	1231.4	861.7	89.1			◉				HMG_box x1 HMG_box_2 x1	NA	NA	NA	NA	NA
44	BSTOLATCC_MAC23079	Contig_6.g1077	792	2398.2	3165.3	1882.2	1579.9	1418.9	1584.3	1508.9	1358.0	1504.5	1421.4	1653.2	0.6							Ribosomal_S4e x1 40S_S4_C x1 F	RPS4	Belongs to the eukaryotic ribosom	NA	Ribosomal protein S4, putative	0.81
45	BSTOLATCC_MAC9923	Contig_30.g352	1101	3564.2	3111.2	1592.2	1555.3	654.7	2955.4	1652.6	2141.4	1502.8	1145.0	2306.9	0.5							Fungal_lectin x2Fungal_lectin x2	NA	NA	NA	NA	NA
46	BSTOLATCC_MAC7895	Contig_25.g1358	948	1844.3	2096.4	1300.4	1761.4	1069.4	1445.3	969.1	1202.4	1486.7	924.8	817.0	0.9							Ribosomal_L5e x1 Ribosomal_L18	RPL5	ribosomal protein	NA	60S ribosomal protein L5	0.53
47	BSTOLATCC_MAC23089	Contig_6.g1087	1002	1565.5	1864.4	1670.3	1286.9	968.8	1118.8	1086.9	1002.8	1483.3	1096.9	1158.8	0.9							RL10P_insert x1 Ribosomal_L10 x1	RPP0	Component of the ribosome, a lar	NA	60S acidic ribosomal protein P0	0.7
48	BSTOLATCC_MAC5313	Contig_20.g360	1104	2156.7	2673.6	2735.9	1671.6	1442.9	1401.4	1535.3	941.5	1431.6	1633.4	1382.2	0.6							Asp x1 TAXi_N x1		Belongs to the peptidase A1 famil	NA	Cathepsin D	0.8
49	BSTOLATCC_MAC3156	Contig_17.g456	1062	2342.1	2915.2	2999.7	2129.8	1494.8	2019.0	1901.5	1543.0	1419.3	1218.7	1719.6	0.5							Peptidase_C1 x1 Inhibitor_I29 x1		Belongs to the peptidase C1 famil	NA	Senescence-specific cysteine prot	0.69
50	BSTOLATCC_MAC3991	Contig_19.g1288	2433	285.7	337.2	384.1	1419.2	825.1	921.9	446.4	931.2	1398.0	549.7	417.5	4.2							Iso_dh x1Iso_dh x1Iso_dh x1	IDH1	Belongs to the isocitrate and isopr	NA	NA	NA
51	BSTOLATCC_MAC4112	Contig_19.g1409	1050	3000.8	3630.0	2142.1	1687.7	1443.7	1812.2	2101.1	1644.2	1389.6	1558.3	2231.4	0.5							Peptidase_C1 x1 Inhibitor_I29 x1		cysteine-type endopeptidase activ	NA	Cathepsin L	0.44
52	BSTOLATCC_MAC1449	Contig_12.g146	631	961.1	1107.5	1458.1	1298.5	1391.3	1074.9	1015.1	1091.6	1386.6	1257.9	1058.8	1.2							Ras x1 Roc x1 Arf x1 MMR_HSR1 x		Rab subfamily of small GTPases	NA	Rab protein 7	0.88
53	BSTOLATCC_MAC11656	Contig_35.g476	1362	3507.0	3286.3	4024.4	3353.9	2466.6	2646.2	1939.1	1490.4	1386.0	1488.1	1261.2	0.4							Tubulin x1 Tubulin_C x1		Tubulin is the major constituent of	NA	Tubulin alpha chain	0.54
54	BSTOLATCC_MAC11524	Contig_34.g345	777	1844.2	2422.6	1886.1	1488.6	1476.7	1255.0	1273.3	1168.4	1382.3	1419.4	1484.9	0.7							Ribosomal_L2_C x1 Ribosomal_L2	RPL8	structural constituent of ribosome	NA	60S ribosomal protein L8	0.54
55	BSTOLATCC_MAC4721	Contig_2.g717	777	1794.4	2257.6	1936.5	1248.0	1249.2	1228.7	1412.6	1143.3	1380.8	1501.9	1774.6	0.7							Ribosomal_L2_C x1 Ribosomal_L2	RPL8	structural constituent of ribosome	NA	60S ribosomal protein L8	0.54
56	BSTOLATCC_MAC23186	Contig_6.g1184	780	1097.4	1118.3	1361.4	1077.0	953.5	1019.5	766.3	930.5	1376.6	990.8	729.5	1.2							Ribosomal_S3Ae x1	RPS3A	Belongs to the eukaryotic ribosom	RPS1	40S ribosomal protein S1	0.49
57	BSTOLATCC_MAC15760	Contig_44.g1003	576	7.8	29.4	16.7	102.1	822.0	697.4	840.6	1266.0	1370.2	1903.7	1632.8	76.3							SAM_1 x1 SAM_2 x1	NA	NA	NA	NA	NA
58	BSTOLATCC_MAC18158	Contig_50.g10	906	2616.2	3282.9	2264.8	1798.8	1648.1	1530.6	2119.5	1524.9	1335.9	1538.9	2767.7	0.5							Peptidase_C1 x1Peptidase_C1 x1		Belongs to the peptidase C1 famil	NA	Cathepsin B	0.59
59	BSTOLATCC_MAC15584	Contig_44.g827	990	2439.4	2946.9	2070.6	1392.1	1137.2	1258.7	1472.9	1188.8	1262.4	1376.8	1628.3	0.5							WD40 x5WD40 x5WD40 x5	RACK1A	component of 40S small ribosoma	NA	Guanine nucleotide-binding protei	0.58
60	BSTOLATCC_MAC5726	Contig_21.g770	939	3.7	6.5	3365.7	4971.6	3160.3	230.6	453.9	498.2	1260.5	3683.6	240.5	1.1							Pkinase x1 PK_Tyr_Ser-Thr x1	CDC28	cyclin-dependent protein serine/th	NA	Cell division control protein 2	0.62
61	BSTOLATCC_MAC8364	Contig_27.g459	402	1604.4	1635.8	1722.8	1189.5	1176.4	1195.1	1252.2	1062.9	1238.6	1224.6	1189.2	0.7							Ribosomal_L32e x1		60S ribosomal protein L32-1-like	RPL32	60S ribosomal protein L32	0.72
62	BSTOLATCC_MAC19969	Contig_55.g411	747	1195.7	1078.4	1612.8	1057.7	1097.7	1075.9	1071.1	1062.7	1236.9	1240.9	1290.5	1.0							14-3-3 x114-3-3 x114-3-3 x1		Belongs to the 14-3-3 family	NA	NA	NA
63	BSTOLATCC_MAC11303	Contig_34.g124	414	14.7	38.3	16.2	45.6	888.7	732.1	1263.0	1417.3	1216.9	1734.7	1857.8	52.8							Histone x1Histone x1Histone x1		protein heterodimerization activity	NA	Histone domain-containing protein	0.5
64	BSTOLATCC_MAC4978	Contig_20.g25	510	1164.2	1736.1	1016.9	741.3	986.2	1378.1	1588.1	1396.5	1207.1	1275.1	1750.5	0.9							zf-C2H2 x1 zf-H2C2_2 x1 zf-Di19 x		Zinc-finger of C2H2 type	NA	NA	NA
65	BSTOLATCC_MAC21189	Contig_57.g399	351	6.0	92.0	46.6	260.6	1419.6	786.0	1028.8	1533.8	1189.8	1752.0	1529.1	24.7							Histone x1Histone x1Histone x1		Core component of nucleosome. I	NA	Histone domain-containing protein	0.1
66	BSTOLATCC_MAC23535	Contig_60.g617	426	1951.9	2072.1	1989.8	1278.6	1307.4	1198.7	1424.3	1064.3	1184.8	1381.2	1544.7	0.6							Ribosomal_L27A x1		Belongs to the universal ribosoma	NA	RL17A, ribosomal protein 27A 60S	0.58
67	BSTOLATCC_MAC10015	Contig_30.g444	1335	2522.3	2394.9	2575.5	2051.9	1457.5	1712.8	1396.8	1166.4	1183.3	991.8	1034.9	0.5							Tubulin x1 Tubulin_C x1 Misat_Tub		Tubulin is the major constituent of	NA	Tubulin beta chain	0.51
68	BSTOLATCC_MAC3117	Contig_17.g417	462	2418.9	2259.4	2668.4	1571.9	1684.5	1703.9	1962.8	1612.6	1168.6	1373.3	1986.8	0.5							NDK x1NDK x1NDK x1NDK x1	awd	nucleoside diphosphate kinase ac	NDK	Nucleoside diphosphate kinase	0.45
69	BSTOLATCC_MAC5291	Contig_20.g338	543	1.7	5.4	3.1	102.8	2131.6	1550.9	2462.4	1970.2	1161.6	1535.0	1168.4	339.5						◉	IF4E x1IF4E x1IF4E x1IF4E x1		translation initiation factor activity	NA	Eukaryotic translation initiation fac	0.22
70	BSTOLATCC_MAC11000	Contig_33.g1425	621	1997.7	2645.5	1598.4	1364.8	1148.5	1193.7	1304.5	1285.3	1156.0	1221.4	1526.2	0.6							Ribosomal_S8e x1	RPS8	Belongs to the eukaryotic ribosom	NA	40S ribosomal protein S8	0.67
71	BSTOLATCC_MAC22403	Contig_59.g122	615	1526.4	1602.6	1772.2	1039.2	959.5	1080.1	885.5	937.0	1142.5	1068.6	998.2	0.7							Ribosomal_L15e x1		Belongs to the eukaryotic ribosom	RPL15	Ribosomal protein L15	0.67
72	BSTOLATCC_MAC15091	Contig_42.g339	276	2724.6	2192.3	1422.6	1265.5	1448.1	1946.0	2100.0	1627.9	1134.6	1499.9	2463.1	0.5							EF-hand_1 x2 EF-hand_5 x2 EF-ha	NA	NA	NA	Calmodulin (Fragment)	0.4
73	BSTOLATCC_MAC12326	Contig_38.g1138	396	1058.5	1184.9	1492.7	1083.0	1179.6	968.5	1037.7	998.0	1122.5	1214.4	1317.0	0.9							DUF2423 x1DUF2423 x1	NA	NA	NA	NA	NA
74	BSTOLATCC_MAC24658	Contig_63.g328	366	1594.3	1614.5	2024.6	1060.8	1040.7	993.6	1058.6	719.6	1103.0	1121.5	1116.7	0.6							Ribosomal_S25 x1	RPS25B	40S ribosomal protein S25	NA	40S ribosomal protein S25	0.6
75	BSTOLATCC_MAC12160	Contig_36.g978	1527	1277.2	1446.8	1101.9	1336.4	756.7	1065.1	693.0	982.8	1092.5	710.8	1020.7	0.9							Aldedh x1Aldedh x1Aldedh x1		Aldehyde dehydrogenase family	MMSA	Methylmalonate-semialdehyde deh	0.49
76	BSTOLATCC_MAC19478	Contig_52.g1321	792	1211.9	1404.5	1089.6	954.8	775.5	970.4	858.7	904.3	1087.0	885.2	1073.8	0.9							Ribosomal_S4e x1 40S_S4_C x1 F	RPS4	Belongs to the eukaryotic ribosom	NA	Ribosomal protein S4, putative	0.8
77	BSTOLATCC_MAC4515	Contig_2.g510	606	1624.3	1956.7	1671.7	1201.9	1101.8	1059.1	1169.7	970.9	1080.6	1159.7	1193.4	0.6							Ribosomal_L13 x1	RPL13A	Belongs to the universal ribosoma	NA	Large subunit ribosomal protein L1	0.72
78	BSTOLATCC_MAC3660	Contig_19.g957	504	1428.6	1486.1	1293.0	899.3	1007.0	1029.3	1226.0	984.1	1080.6	1295.7	1451.0	0.8							Profilin x1Profilin x1Profilin x1		Profilin	NA	Profilin	0.56
79	BSTOLATCC_MAC13936	Contig_40.g852	1467	963.8	953.4	1158.0	971.2	585.4	717.1	559.4	704.9	1078.0	713.0	863.6	1.1							ELFV_dehydrog x1 ELFV_dehydro	gdhA	Belongs to the Glu Leu Phe Val d	GDHA	Glutamate dehydrogenase	0.45
80	BSTOLATCC_MAC12384	Contig_38.g1196	1617	1153.0	1085.3	803.8	1091.8	808.6	1031.9	760.0	932.5	1061.7	780.6	885.4	1.0							DEAD x1 Helicase_C x1 ResIII x1 E	DBP2	Belongs to the DEAD box helicase	NA	Similar to Saccharomyces cerevisia	0.42
81	BSTOLATCC_MAC18506	Contig_50.g358	390	1652.8	1546.8	1276.8	990.1	876.0	1007.2	1112.3	1004.6	1060.2	1029.5	1357.6	0.7							ubiquitin x1 Ribosomal_L40e x1 Ra		Ribosomal L40e family	NA	Ubiquitin	0.59
82	BSTOLATCC_MAC9163	Contig_29.g8	1146	322.1	764.9	981.8	1642.7	1150.3	782.0	837.5	854.3	1051.2	930.4	537.8	1.5							ubiquitin x5 Rad60-SLD x5		ubiquitin	NA	Polyubiquitin	0.64

83	BSTOLATCC_MAC15740	Contig_44.g983	531	2170.2	2017.5	1904.8	1409.7	1295.7	1480.2	1567.8	1326.1	1044.9	1023.8	1088.3	0.5							EF-hand_7 x2 EF-hand_6 x1 EF-ha	CEN2	EF-hand domain	NA	Centrin-2	0.69
84	BSTOLATCC_MAC23416	Contig_60.g498	1221	986.0	1020.5	1400.6	1276.0	1013.4	1143.0	690.6	858.4	1042.8	789.1	697.3	0.9							2-Hacid_dh_C x1 2-Hacid_dh x1 N/		D-isomer specific 2-hydroxyacid de	NA	2-oxoglutarate reductase	0.5
85	BSTOLATCC_MAC11902	Contig_36.g720	639	1054.1	1503.7	1370.5	1010.2	1100.5	849.7	864.3	868.5	1039.6	1045.7	1039.4	0.8							Ribosomal_L16 x1		Ribosomal protein L16p/L10e	RL10	60S ribosomal protein L10	0.58
86	BSTOLATCC_MAC22770	Contig_6.g768	1812	172.7	257.9	299.9	500.3	1129.4	554.5	785.2	740.6	1037.4	1180.0	773.3	4.3							RRM_1 x4 PABP x1		Binds the poly(A) tail of mRNA	NA	NA	NA
87	BSTOLATCC_MAC23362	Contig_60.g444	462	833.8	1204.5	931.9	637.9	843.8	1215.5	1237.9	1213.2	1030.3	991.6	2040.4	1.0							zf-CCCH x1 zf_CCCH_4 x1 zf-CCCI		3'-UTR-mediated mRNA destabiliz	NA	NA	NA
88	BSTOLATCC_MAC3493	Contig_18.g792	1422	507.9	748.5	581.7	616.4	666.4	642.3	726.2	680.8	1011.5	890.7	877.7	1.7							Peptidase_M20 x1 M20_dimer x1	dapE1	peptidase M20	NA	Acetylornithine deacetylase/Succir	0.39
89	BSTOLATCC_MAC9037	Contig_28.g1125	456	2296.6	2347.8	1657.8	1186.2	1135.5	1352.8	1538.3	1347.4	1011.3	1230.3	1995.7	0.5							Ribosomal_S13_N x1 Ribosomal_S	RPS13	Belongs to the universal ribosoma	NA	40S ribosomal protein S13	0.58
90	BSTOLATCC_MAC1654	Contig_12.g351	444	2219.0	2381.2	1516.5	1045.8	967.4	1218.8	1727.8	1256.8	1010.2	1014.9	1699.8	0.5							UQ_con x1UQ_con x1UQ_con x1	ubcB	Ubiquitin-conjugating enzyme E2,	NA	Ubiquitin conjugating enzyme, put	0.49
91	BSTOLATCC_MAC12990	Contig_39.g185	462	483.2	603.4	1159.0	1623.8	1354.6	648.2	793.6	672.7	997.6	1151.5	736.1	1.3							ubiquitin x2 Rad60-SLD x2 Ubiquiti	UBI4	ubiquitin	NA	Polyubiquitin	0.62
92	BSTOLATCC_MAC1604	Contig_12.g301	1467	1818.6	2022.6	1007.9	960.0	762.9	1194.4	970.5	1230.5	995.6	940.3	1481.2	0.6							PALP x1 CBS x1PALP x1 CBS x1	CYS4	Belongs to the cysteine synthase	NA	Cystathionine beta-synthase	0.59
93	BSTOLATCC_MAC21712	Contig_57.g922	453	1816.4	1609.7	1416.0	1063.6	881.1	1190.5	1002.8	1092.3	994.9	1049.0	1240.8	0.6							Ribosomal_L24e x1	RPL24	60S ribosomal protein L24. Sourc	NA	60S ribosomal protein L24	0.45
94	BSTOLATCC_MAC25165	Contig_7.g1579	930	1404.3	1352.5	1995.0	1104.5	1041.2	1080.8	1375.4	862.6	986.0	1148.2	1049.7	0.6							Mito_carr x3Mito_carr x3	SLC25A4	Belongs to the mitochondrial carri	NA	ADP/ATP translocase 1	0.56
95	BSTOLATCC_MAC22656	Contig_6.g654	672	2862.0	3056.3	1808.2	1534.0	950.2	2245.8	1981.5	1819.9	981.6	902.6	1872.9	0.4							START x1START x1START x1	NA	NA	NA	NA	NA
96	BSTOLATCC_MAC4293	Contig_19.g1590	422	1357.8	1061.5	1511.0	1205.4	980.8	1080.7	976.6	841.7	973.5	987.7	870.8	0.7							Ribosomal_L14e x1	RPL14	ribosomal protein	RPL14	60S ribosomal protein L14	0.85
97	BSTOLATCC_MAC9061	Contig_28.g1149	798	1905.3	2230.7	1628.3	1114.6	1099.9	1207.2	1409.5	998.4	968.7	1074.9	1726.6	0.5							C2 x1C2 x1C2 x1C2 x1C2 x1C2 x1		Protein kinase C conserved regior	NA	C2 domain-containing protein	0.46
98	BSTOLATCC_MAC11139	Contig_33.g1564	639	2449.1	3034.7	1652.6	1289.7	1166.2	1313.1	1439.4	1227.6	958.0	1050.9	1427.8	0.4							Ribosomal_L16 x1		Ribosomal protein L16p/L10e	RL10	60S ribosomal protein L10	0.58
99	BSTOLATCC_MAC16146	Contig_45.g1388	432	1454.3	1422.0	1644.9	963.9	964.2	935.9	1173.3	912.6	936.7	1049.9	1090.1	0.6							Ribosom_S12_S23 x1		40s ribosomal protein	NA	40S ribosomal protein S23	0.61
##	BSTOLATCC_MAC23848	Contig_61.g925	876	1170.4	1162.8	1047.6	864.8	711.1	1028.9	898.6	854.4	936.4	739.8	749.5	0.8							Porin_3 x1Porin_3 x1Porin_3 x1	NA	NA	NA	NA	NA

Table S4. Top 100 genes in *Blepharisma stoltei* ATCC 30299 most upregulated at 26 hours vs. the average of starved, gamone-treated and 0-hour gene expression.

***B. stoltei* ATCC 30299 MAC gene expression** (only genes with PFAM annotations; domain multiplicity indicated by "x" and a number; blue = 0-1 RPKM; cyan = 1-10 RPKM; 10-100 RPKM = yellow; 100-1000 RPKM = orange; 1000-10000 RPKM = red; gene expression divisions with zero denominator are at the bottom of the data set, with the genes involved showing little, if any, expression throughout)

	ENA accession	Gene ID	Length (CDS in bp)	Starved	Gamone-treated	0 h	2 h	6 h	14 h	18 h	22 h	26 h	30 h	38 h	26h/average(starved +gamone+0h)	Transposase	DNA repair	Chromatin	Transcription	Small RNAs	Translation	PFAM Domains	eggNOG gene name	eggNOG description	Pannzer gene name	Pannzer description	Pannzer PPV
1	BSTOLATCC_MAC22820	Contig_6.g818	3780	0.3	0.5	0.2	1.4	51.7	101.1	105.4	209.8	278.1	118.7	65.5	824.9		◉					STAG x1	SA	STAG domain	NA	SCD domain-containing protein	1
2	BSTOLATCC_MAC12076	Contig_36.g894	927	0.1	0.2	0.1	0.2	1.7	6.4	5.7	17.2	50.9	89.7	64.2	514.0							CENP-B_N x1 DUF2969 x1	NA	NA	NA	NA	NA
3	BSTOLATCC_MAC15299	Contig_43.g546	1335	0.0	0.1	0.1	0.5	22.9	25.7	24.7	53.7	48.5	35.7	23.4	507.9		◉					BRCT_2 x1	NA	NA	NA	NA	NA
4	BSTOLATCC_MAC7513	Contig_25.g976	348	0.0	0.0	0.4	37.4	77.2	34.8	29.4	59.7	68.3	40.6	26.3	506.7						◉	HSP90 x1	HSP90	Heat shock protein	NA	Heat shock protein 90	0
5	BSTOLATCC_MAC7514	Contig_25.g977	1638	0.0	0.0	0.5	48.0	101.3	44.7	36.4	71.8	84.9	54.3	32.2	478.6						◉	HSP90 x1 HATPase_c x1 HATPa	NA	heat shock protein	NA	Heat shock protein 90	1
6	BSTOLATCC_MAC12987	Contig_39.g182	3876	0.1	0.3	0.2	5.9	23.3	32.3	23.6	68.7	74.7	24.4	18.0	387.5				◉			DUF3591 x1 Bromodomain x1	Taf1	binding. It is involved in the biol	NA	NA	NA
7	BSTOLATCC_MAC5291	Contig_20.g338	543	1.7	5.4	3.1	102.8	2131.6	1550.9	2462.4	1970.2	1161.6	1535.0	1168.4	339.5						◉	IF4E x1	NA	translation initiation factor activit	NA	Eukaryotic translation initiation f	0
8	BSTOLATCC_MAC10792	Contig_33.g1217	669	0.1	0.4	0.1	2.3	188.0	152.0	220.0	156.4	69.5	79.9	70.7	338.0							14-3-3 x1	NA	14-3-3 homologues	NA	NA	NA
9	BSTOLATCC_MAC24980	Contig_7.g1394	396	0.1	0.5	0.1	1.2	72.4	57.5	99.4	107.9	70.8	97.4	79.5	297.5							zf-C2H2 x2 zf-C2H2_4 x2 zf-H2C	NA	NA	NA	NA	NA
10	BSTOLATCC_MAC2188	Contig_13.g876	1590	0.1	0.1	0.1	0.3	3.7	7.9	10.7	19.1	34.3	47.2	37.2	290.3	◉						DDE_Tnp_1_7 x1	NA	Transposase IS4	NA	NA	NA
11	BSTOLATCC_MAC17155	Contig_48.g755	1896	7.1	12.3	9.7	60.2	3458.6	4149.6	2813.3	2326.9	2799.4	2446.1	1305.3	288.1							DEAD x1 zf-CCHC x4 Helicase_C	DBP2	Belongs to the DEAD box helica	DDX43	RNA helicase	0
12	BSTOLATCC_MAC5406	Contig_20.g453	2340	4.5	9.5	10.1	150.6	6059.2	4134.1	3321.1	2959.9	2282.3	1941.8	1102.0	283.3					◉		Piwi x1 PAZ x1 ArgoL1 x1	NA	Piwi	NA	NA	NA
13	BSTOLATCC_MAC711	Contig_10.g883	717	0.6	1.4	0.5	2.1	51.5	90.8	97.2	190.8	235.5	342.6	203.8	280.7				◉			CID x1	NA	NA	NA	CID domain-containing protein	1
14	BSTOLATCC_MAC4276	Contig_19.g1573	879	0.5	0.6	1.0	13.6	198.9	163.8	191.7	266.7	200.5	255.2	175.1	279.5							zf-B_box x1	NA	NA	NA	NA	NA
15	BSTOLATCC_MAC23590	Contig_60.g672	3246	0.5	0.7	0.3	9.4	45.7	59.4	40.9	128.3	146.3	47.3	35.1	269.7			◉				JmjC x1	NA	A domain family that is part of th	NA	JmjC domain-containing protein	0
16	BSTOLATCC_MAC6526	Contig_22.g1567	1923	0.0	0.0	0.0	0.2	0.0	0.6	1.8	0.1	1.8	0.1	0.1	258.8							UCH x1	NA	ubiquitin carboxyl-terminal hydro	NA	NA	NA
17	BSTOLATCC_MAC6719	Contig_23.g186	633	0.6	0.5	0.4	1.9	51.8	80.2	56.0	165.0	125.6	183.5	93.9	254.7							CENP-B_N x1 ubiquitin x1 HTH_	NA	NA	NA	NA	NA
18	BSTOLATCC_MAC11436	Contig_34.g257	525	1.0	1.9	0.7	1.5	67.0	116.3	139.2	262.7	279.8	374.8	321.0	235.3			◉				Chromo x1	NA	Chromo (CHRromatin Organisati	NA	NA	NA
19	BSTOLATCC_MAC22509	Contig_59.g228	1614	0.0	0.0	0.0	0.1	1.0	2.7	2.2	5.3	6.5	8.5	5.9	230.3							Kelch_6 x1	NA	NA	NA	NA	NA
20	BSTOLATCC_MAC12942	Contig_39.g137	393	0.3	0.1	0.0	0.2	35.0	27.6	40.6	37.8	33.3	50.0	41.4	230.1							zf-C2H2_4 x2 zf-C2H2 x2 zf-H2C	NA	NA	NA	NA	NA
21	BSTOLATCC_MAC12676	Contig_38.g1488	258	0.5	1.8	0.9	6.6	317.8	232.6	546.2	439.4	241.7	273.4	232.3	223.0				◉		◉	LSM14 x1 SM-ATX x1	NA	NA	NA	NA	NA
22	BSTOLATCC_MAC5920	Contig_21.g964	540	0.0	0.0	0.2	0.8	10.3	6.2	5.1	9.6	12.6	8.2	3.7	217.7						◉	HSP70 x1	HSP70	heat shock protein 70	NA	Heat shock 70 kDa protein (Frag	1
23	BSTOLATCC_MAC5919	Contig_21.g963	765	0.0	0.0	0.1	0.7	8.0	5.4	4.0	7.3	10.6	5.5	3.1	216.5						◉	HSP70 x1 MreB_Mbl x1	NA	Belongs to the heat shock prote	HSP70	Heat shock protein 70 (Fragmen	0
24	BSTOLATCC_MAC14490	Contig_41.g1404	1590	1.8	1.4	1.8	4.6	40.7	117.4	139.0	257.0	342.5	267.0	177.2	205.5	◉						DDE_3 x1	NA	DDE superfamily endonuclease	NA	NA	NA
25	BSTOLATCC_MAC23800	Contig_60.g882	2058	0.6	0.5	0.6	1.7	39.8	49.7	54.1	107.0	111.4	51.2	30.2	201.5							DEAD x1 Helicase_C x1 ResIII x1	DDX25	RNA helicase activity	NA	NA	NA
26	BSTOLATCC_MAC18054	Contig_5.g529	1812	0.3	0.1	0.3	0.4	5.3	15.3	16.6	30.4	40.5	33.5	25.6	195.6	◉						MULE x1 Transposase_mut x1	NA	Protein FAR1-RELATED SEQUE	NA	NA	NA
27	BSTOLATCC_MAC1548	Contig_12.g245	1554	0.4	0.9	2.4	1.6	33.3	93.7	112.5	153.6	232.4	254.3	168.5	191.1		◉					Rad21_Rec8_N x1	RAD21	positive regulation of sister chro	NA	NA	NA
28	BSTOLATCC_MAC13455	Contig_4.g167	1164	0.1	0.2	0.0	0.1	8.0	14.1	18.9	22.6	19.3	21.7	12.7	190.2							NTP_transf_2 x1 PAP_assoc x1	NA	RNA uridylyltransferase activity	NA	Non-canonical poly(A) RNA poly	0
29	BSTOLATCC_MAC2214	Contig_13.g902	891	0.2	0.1	0.4	0.3	13.1	19.2	23.2	35.5	40.2	49.6	24.1	187.0							zf-RING_UBOX x1 zf-RING_5 x1	NA	regulation of erythrocyte enucle	NA	NA	NA
30	BSTOLATCC_MAC5044	Contig_20.g91	2136	2.2	2.3	2.9	4.8	80.8	193.3	212.6	370.6	431.5	397.0	250.7	174.1			◉				JmjC x1	NA	A domain family that is part of th	NA	NA	NA
31	BSTOLATCC_MAC6514	Contig_22.g1555	1497	0.0	0.0	0.0	0.1	0.3	0.2	1.1	0.4	0.7	0.0	0.4	167.7							UCH x1	NA	ubiquitin carboxyl-terminal hydro	NA	NA	NA
32	BSTOLATCC_MAC17684	Contig_49.g1283	2916	0.3	0.7	0.2	17.5	48.4	47.8	31.6	69.7	68.5	28.6	19.9	167.5			◉				SNF2_N x1 SLIDE x1 Helicase_C	NA	helicase superfamily c-terminal d	NA	Transcription activator snf2l1	1
33	BSTOLATCC_MAC16927	Contig_48.g527	960	1.0	2.9	1.2	11.7	86.5	151.9	155.9	297.4	282.5	227.8	153.1	166.9			◉				Chromo x1 RNA_pol_Rpb2_6 x1	NA	Chromatin organization modifier	NA	NA	NA
34	BSTOLATCC_MAC16237	Contig_45.g1479	561	1.8	1.2	1.3	2.3	176.8	204.1	283.6	321.3	234.9	287.4	180.3	164.4							zf-RanBP x2	NA	Zinc finger domain	NA	NA	NA
35	BSTOLATCC_MAC16168	Contig_45.g1410	1944	0.3	0.2	1.2	2.6	52.0	39.3	36.6	62.0	88.5	62.7	31.9	157.8						◉	HSP70 x1 MreB_Mbl x1	NA	heat shock protein 70	NA	Cytosol-type hsp70	1
36	BSTOLATCC_MAC15955	Contig_45.g1197	456	1.3	0.8	1.2	18.5	184.8	129.7	182.8	216.3	163.8	187.7	97.6	147.6							TCR x2	NA	Tesmin/TSO1-like CXC domain	NA	NA	NA
37	BSTOLATCC_MAC18055	Contig_5.g530	1812	0.3	0.4	0.4	1.3	4.7	13.3	16.3	30.1	54.6	64.9	43.0	145.2	◉						MULE x1	NA	FAR1 DNA-binding domain	NA	NA	NA
38	BSTOLATCC_MAC15249	Contig_43.g496	1902	0.5	0.3	0.2	1.0	8.6	18.8	20.4	44.5	46.0	36.7	26.3	142.2	◉						MULE x1	NA	MULE transposase domain	NA	NA	NA
39	BSTOLATCC_MAC17007	Contig_48.g607	1218	0.1	0.1	0.4	1.4	3.2	2.9	3.2	7.3	28.9	23.3	12.0	141.3							Bromodomain x1 BET x1	BRDT	bromo domain	NA	NA	NA
40	BSTOLATCC_MAC12545	Contig_38.g1357	1542	0.3	0.2	0.1	2.6	11.3	12.3	12.3	25.5	26.8	14.6	9.2	140.3		◉					BRCT_2 x1	NA	NA	NA	NA	NA
41	BSTOLATCC_MAC1138	Contig_11.g1307	2256	1.6	0.8	1.1	4.3	43.2	83.3	80.7	142.1	158.8	123.8	75.5	136.5					◉		Ribonuclease_3 x2 Ribonucleas_	dcl1	Mortierella verticillata NRRL 633	NA	NA	NA
42	BSTOLATCC_MAC10037	Contig_30.g466	2151	1.2	1.2	1.0	7.3	66.6	81.7	65.9	152.1	149.7	94.6	66.4	133.3				◉			NIF x1	fcp1	catalytic domain of ctd-like phos	NA	NA	NA
43	BSTOLATCC_MAC9080	Contig_28.g1168	1581	0.6	0.5	0.9	2.4	16.1	33.9	31.2	65.7	86.8	91.4	60.5	130.0							PB1 x1	NA	NA	NA	NA	NA
44	BSTOLATCC_MAC1071	Contig_11.g1240	1992	2.0	1.7	1.3	8.2	91.8	113.6	108.7	219.2	209.8	146.6	111.5	128.8							Dynamin_M x1 Dynamin_N x1 GE	NA	Belongs to the TRAFAC class d	NA	Dynamin-related protein 3A	1
45	BSTOLATCC_MAC25739	Contig_9.g358	1014	1.2	1.0	0.7	2.6	44.6	71.5	76.7	150.0	120.8	130.3	80.3	126.5							zf-B_box x1 zf-RING_5 x1 zf-RIN	NA	zinc ion binding	NA	NA	NA
46	BSTOLATCC_MAC7356	Contig_25.g819	2061	0.4	0.3	0.6	6.7	56.7	49.9	36.9	56.0	51.7	36.6	24.6	124.4							FH2 x1	GRID2IP	Glutamate receptor, ionotropic,	NA	NA	NA
47	BSTOLATCC_MAC1470	Contig_12.g167	783	1.4	1.3	1.0	1.2	29.0	65.8	97.1	126.6	142.6	218.1	186.1	116.0		◉					PCNA_N x1 PCNA_C x1 Rad1 x1	NA	This protein is an auxiliary protei	NA	Proliferating cell nuclear antigen	0
48	BSTOLATCC_MAC11469	Contig_34.g290	651	0.8	1.4	2.5	3.9	73.4	103.7	138.6	173.1	175.8	278.8	208.9	114.4				◉			TBP x2	SPT15	Transcription factor TFIID (or TA	NA	Tata-binding general transcriptic	1
49	BSTOLATCC_MAC20942	Contig_57.g152	435	2.7	10.7	3.1	9.0	579.9	497.9	943.6	834.7	622.9	982.3	1137.5	113.2							zf-H2C2_2 x1	NA	NA	NA	NA	NA
50	BSTOLATCC_MAC13720	Contig_40.g636	4278	1.2	2.6	1.1	37.1	75.0	83.3	67.8	144.7	182.9	83.7	59.0	111.7							Nipped-B_C x1 Cnd1 x1 PHD x1	NIPBL	Sister chromatid cohesion C-terr	NA	NA	NA
51	BSTOLATCC_MAC23155	Contig_6.g1153	1380	0.2	0.7	0.6	0.3	6.1	14.0	20.5	27.1	59.9	115.1	79.7	110.3		◉					zf-GRF x2 Exo_endo_phos x1	APEX2	double-stranded DNA 3'-5' exod	NA	DNA-(apurinic or apyrimidinic site	0
52	BSTOLATCC_MAC1521	Contig_12.g218	567	1.3	6.5	2.8	5.7	175.3	170.8	196.2	315.0	387.9	516.2	444.0	108.8							SAM_1 x1 DUF1805 x1	NA	NA	NA	NA	NA
53	BSTOLATCC_MAC25840	Contig_9.g459	402	1.6	5.1	7.6	4.9	233.0	328.6	626.3	576.4	517.1	851.2	682.6	108.8			◉				Histone x1 Histone_H2A_C x1 CE	NA	Histone H2AX	NA	NA	NA
54	BSTOLATCC_MAC16265	Contig_45.g1507	2055	0.0	0.0	0.0	1.0	2.7	4.1	1.7	2.5	1.7	1.3	0.9	104.5							AAA-ATPase_like x1	NA	Psort location Cytoplasmic, scor	NA	NA	NA
55	BSTOLATCC_MAC23523	Contig_60.g605	1536	1.7	1.3	1.6	26.6	335.8	220.4	195.8	240.1	162.0	99.3	60.6	104.2				◉			RRM_1 x4 PABP x1	NA	Binds the poly(A) tail of mRNA	NA	NA	NA
56	BSTOLATCC_MAC21072	Contig_57.g282	1215	0.3	0.2	0.2	0.9	3.9	6.6	7.3	14.4	23.5	25.5	16.7	104.0							ERCC4 x1	mus81	It is involved in the biological prc	NA	NA	NA
57	BSTOLATCC_MAC20619	Contig_56.g1053	3813	0.6	1.8	0.6	41.0	56.3	42.8	35.6	81.9	104.8	40.3	25.3	103.3							DNA_topoisoIV x1 TOPRIM_C x1	NA	Control of topological states of D	NA	DNA topoisomerase 2	1
58	BSTOLATCC_MAC20673	Contig_56.g1107	1245	0.5	0.7	0.3	23.1	66.7	23.3	23.2	60.4	51.5	31.0	19.3	103.2							TolA_bind_tri x1	NA	NA	NA	NA	NA
59	BSTOLATCC_MAC4026	Contig_19.g1323	828	0.0	0.0	0.1	0.2	1.0	3.4	3.7	5.0	3.6	4.2	4.3	101.9							START x1	NA	NA	NA	NA	NA
60	BSTOLATCC_MAC18052	Contig_5.g527	1221	1.5	3.8	3.5	5.1	75.0	141.0	160.6	252.5	291.8	348.1	289.2	100.1	◉						DDE_Tnp_IS1595 x1	NA	ISXO2-like transposase domain	NA	NA	NA
61	BSTOLATCC_MAC1058	Contig_11.g1227	2148	0.6	1.0	1.5	8.2	42.7	55.3	50.1	93.6	101.8	62.4	41.2	99.7		◉					PARP x1 PARP_reg x1 WGR x1	PARP2	WGR domain containing protein	NA	Poly [ADP-ribose] polymerase	0
62	BSTOLATCC_MAC6181	Contig_22.g1222	471	0.2	0.4	0.2	1.0	11.0	24.0	17.0	36.2	28.4	49.2	48.8	98.7							RNA_GG_bind x1	NA	NA	NA	NA	NA
63	BSTOLATCC_MAC13381	Contig_4.g93	1680	0.5	0.3	0.9	15.3	38.1	32.0	28.7	64.7	58.1	32.7	21.2	98.5							Kinesin x1 Microtub_bd x1	NA	Belongs to the TRAFAC class m	KIFC1	kinesin-like protein KIFC1 isofor	1
64	BSTOLATCC_MAC13030	Contig_39.g225	1101	1.2	2.3	0.9	6.8	95.0	110.0	114.5	174.5	141.0	138.2	59.3	98.2							RRM_2 x1	AML1	RNA binding	NA	NA	NA
65	BSTOLATCC_MAC17965	Contig_5.g440	1692	5.2	6.5	5.5	15.8	131.3	231.9	256.1	415.8	559.4	476.3	329.9	97.5	◉						DDE_3 x1	NA	Transposase	NA	NA	NA
66	BSTOLATCC_MAC14246	Contig_41.g1160	1854	0.3	0.4	1.3	4.8	13.0	23.9	23.8	47.3	61.1	57.8	43.6	94.2	◉						DDE_1 x1 HTH_Tnp_Tc5 x1 CEN	JRKL	Jerky protein homolog-like	NA	NA	NA

67	BSTOLATCC_MAC5266	Contig_20.g313	420	0.5	0.3	0.8	2.9	50.6	51.5	78.8	81.6	49.6	74.8	44.3	92.9			◉				CBFD_NFYB_HMF x1 Histone x1	NA	reference M ller, K., Lindauer, A	NA	NA	NA
68	BSTOLATCC_MAC3243	Contig_18.g542	816	3.1	3.9	1.8	3.6	80.5	86.6	109.9	192.0	270.8	441.2	325.1	92.4							Chromo x1	NA	NA	NA	NA	NA
69	BSTOLATCC_MAC21908	Contig_58.g1104	1626	0.2	0.5	0.3	0.4	4.1	9.9	12.1	20.5	30.0	44.0	42.2	91.6	◉						DDE_3 x1	NA	DDE superfamily endonuclease	NA	NA	NA
70	BSTOLATCC_MAC15011	Contig_42.g259	576	2.7	2.1	2.2	3.9	42.3	80.1	107.9	159.7	210.8	358.4	206.8	90.4							BAH x1 PHD x1 PHD_2 x1	NA	BAH domain	NA	NA	NA
71	BSTOLATCC_MAC14030	Contig_40.g946	546	0.4	33.4	17.0	721.8	4701.6	2241.2	3048.8	3099.9	1508.7	1231.4	861.7	89.1			◉				HMG_box x1 HMG_box_2 x1	NA	NA	NA	NA	NA
72	BSTOLATCC_MAC7803	Contig_25.g1266	2151	7.1	8.5	8.2	77.0	402.3	335.0	316.1	601.5	699.6	448.7	254.6	88.3				◉			Spt5-NGN x1 KOW x2 Spt5_N x1	NA	Transcription elongation factor S	NA	NA	NA
73	BSTOLATCC_MAC16643	Contig_46.g251	1158	0.5	0.8	1.5	1.5	31.2	73.2	100.9	116.3	82.7	109.6	79.3	87.5		◉					DRMBL x1 Lactamase_B_2 x1	DCLRE1	DNA repair metallo-beta-lactama	NA	DRMBL domain-containing prote	0
74	BSTOLATCC_MAC3873	Contig_19.g1170	2829	0.9	0.6	0.6	15.9	90.2	46.9	33.8	59.0	61.1	56.7	35.2	87.5							Xpo1 x1	NA	Exportin 1-like protein	NA	NA	NA
75	BSTOLATCC_MAC20542	Contig_56.g976	342	4.1	12.5	12.5	34.7	602.2	487.7	782.3	865.4	846.2	1271.3	1381.0	87.3			◉				CENP-T_C x1 CENP-S x1 TAF x	NA	protein heterodimerization activi	NA	NA	NA
76	BSTOLATCC_MAC1496	Contig_12.g193	1728	1.4	0.7	1.0	3.1	30.9	53.0	48.3	101.6	86.7	76.4	57.9	85.2							POT1 x1	NA	NA	NA	NA	NA
77	BSTOLATCC_MAC2159	Contig_13.g847	3663	0.2	0.6	0.2	12.5	16.6	15.9	11.1	30.2	28.3	9.5	6.9	82.7		◉					AAA_23 x1 SMC_N x1 AAA_29 x	RAD50	AAA domain	NA	NA	NA
78	BSTOLATCC_MAC2096	Contig_13.g784	3102	1.3	1.3	2.5	15.9	83.5	74.9	60.6	136.6	139.5	74.9	54.3	82.4							AAA_12 x1 AAA_11 x1 AAA_30	NA	AAA domain	NA	NA	NA
79	BSTOLATCC_MAC18270	Contig_50.g122	432	0.3	1.1	0.5	0.3	4.8	10.5	13.9	21.2	52.8	127.1	102.8	81.5			◉				HMG_box_2 x1 HMG_box x1	NA	NA	NA	NA	NA
80	BSTOLATCC_MAC23585	Contig_60.g667	1311	1.5	1.1	1.2	1.6	30.1	52.1	54.3	84.3	102.1	102.5	55.9	81.0							PRiA4_ORF3 x1	NA	NA	NA	NA	NA
81	BSTOLATCC_MAC2210	Contig_13.g898	1035	0.3	0.4	0.0	0.4	5.2	11.3	14.2	23.6	19.8	20.2	11.7	80.7							zf-RING_5 x1 zf-RING_2 x1 zf-RI	NA	NA	NA	NA	NA
82	BSTOLATCC_MAC6689	Contig_23.g156	1770	7.6	13.2	12.4	55.7	1993.4	2038.7	1002.1	604.3	881.5	941.1	499.5	79.8							DEAD x1 Helicase_C x1 zf-CCHC	DBP2	Belongs to the DEAD box helica	NA	DEAD box RNA helicase-like prc	1
83	BSTOLATCC_MAC8561	Contig_27.g656	3987	0.1	0.9	0.2	18.0	22.6	18.7	8.7	26.6	32.1	10.7	6.2	78.7							Kinesin x1 Microtub_bd x1	NA	Belongs to the TRAFAC class m	NA	NA	NA
84	BSTOLATCC_MAC13434	Contig_4.g146	612	2.3	3.5	2.1	2.9	135.5	177.0	291.4	238.2	208.0	291.4	322.1	78.7							zf-RING_2 x1 zf-C3HC4_2 x1 zf-F	NA	E3 ubiquitin-protein ligase RING	NA	NA	NA
85	BSTOLATCC_MAC17791	Contig_49.g1390	1164	1.8	3.3	1.5	2.0	35.5	76.5	98.4	134.1	169.7	227.1	167.7	77.9							Pre-SET x1	NA	NA	NA	NA	NA
86	BSTOLATCC_MAC6422	Contig_22.g1463	342	2.1	7.7	4.4	3.8	166.5	209.8	242.3	357.4	366.4	504.3	527.9	77.6			◉				CENP-T_C x1 CENP-S x1 TAF x	NA	protein heterodimerization activi	NA	NA	NA
87	BSTOLATCC_MAC25631	Contig_8.g253	1584	2.4	1.6	3.3	27.8	142.7	91.8	91.4	158.7	182.8	156.2	89.5	76.5							ThiF x1 UAE_UbL x1 UBA_e1_th	SAE2	Ubiquitin/SUMO-activating enzy	NA	SUMO-activating enzyme subun	0
88	BSTOLATCC_MAC15760	Contig_44.g1003	576	7.8	29.4	16.7	102.1	822.0	697.4	840.6	1266.0	1370.2	1903.7	1632.8	76.3							SAM_1 x1 SAM_2 x1	NA	NA	NA	NA	NA
89	BSTOLATCC_MAC9851	Contig_30.g279	570	1.9	4.0	5.1	9.4	195.4	227.4	379.4	360.8	280.1	512.2	409.2	76.1							zf-C3HC4 x1 zf-RING_2 x1	NA	zinc finger CCCH domain-contai	NA	NA	NA
90	BSTOLATCC_MAC22628	Contig_6.g626	1203	0.8	0.4	0.3	1.2	4.5	8.4	8.3	17.2	39.4	64.1	56.9	76.0							HUN x1	NA	NA	NA	NA	NA
91	BSTOLATCC_MAC16275	Contig_45.g1517	3216	0.4	0.5	0.1	14.4	23.1	15.6	12.3	28.0	25.2	15.5	9.9	75.9							AAA-ATPase_like x1	NA	An automated process has iden	NA	NA	NA
92	BSTOLATCC_MAC10232	Contig_30.g661	939	5.1	5.0	4.2	5.7	56.9	109.8	123.3	242.5	362.2	415.0	273.5	75.8				◉			Med26 x1	NA	Transcription elongation factor (	NA	TFIIS N-terminal domain-contain	1
93	BSTOLATCC_MAC5461	Contig_20.g508	372	0.1	0.1	0.3	0.8	10.9	8.3	16.5	13.2	11.4	17.9	16.7	74.6							EF-hand_8 x1 EF-hand_6 x1 EF	NA	NA	NA	NA	NA
94	BSTOLATCC_MAC6118	Contig_22.g1159	888	2.2	1.4	2.5	3.1	55.6	95.9	91.8	160.0	148.2	158.3	93.7	73.6		◉					BRCT_2 x1	NA	NA	NA	NA	NA
95	BSTOLATCC_MAC8474	Contig_27.g569	912	0.9	2.9	1.4	40.0	223.2	127.6	163.6	169.1	126.4	179.2	117.1	73.5							Pkinase x1 PK_Tyr_Ser-Thr x1	CDC28	cyclin-dependent protein serine/	CDK1	Cell division control protein 2	0
96	BSTOLATCC_MAC20251	Contig_55.g693	390	1.9	7.6	15.3	13.9	395.0	404.3	541.4	792.2	604.5	592.2	398.0	73.1			◉				Histone x1 CBFD_NFYB_HMF x1	H2AFY	Histone H2A	NA	NA	NA
97	BSTOLATCC_MAC9213	Contig_29.g58	3291	0.9	1.9	1.0	19.6	51.3	58.5	50.7	100.8	89.6	55.9	46.0	71.2							AAA-ATPase_like x2	NA	An automated process has iden	NA	NA	NA
98	BSTOLATCC_MAC10766	Contig_33.g1191	408	0.0	1.1	0.6	0.3	8.7	18.4	36.6	34.9	39.8	85.9	53.1	71.1							zf-C2H2_jaz x1 zf-Di19 x1	NA	NA	NA	NA	NA
99	BSTOLATCC_MAC5921	Contig_21.g965	387	0.0	0.1	0.4	0.8	7.3	4.9	2.8	8.2	10.7	6.7	2.6	69.4						◉	HSP70 x1	NA	ATP binding	HSP70	Heat shock protein 70 (Fragmer	0
##	BSTOLATCC_MAC23646	Contig_60.g728	945	0.6	0.7	0.6	0.4	14.0	32.8	40.8	47.3	45.5	102.2	100.5	69.1		◉					Exo_endo_phos x1	APEX1	double-stranded DNA 3'-5' exod	NA	DNA-(apurinic or apyrimidinic site	0

Table S5. Substitution rates between *Paramecium tetraurelia* PiggyMac-like genes and PiggyMac (Reference gene: PGM - PTET.51.1.G0490162).

Gene abbreviation	*P. tetraurelia* gene ID	d_N/d_S	d_N	d_S
PGML2	PTET.51.1.G0380073	0.0773	1.1082	14.3409
PGML3a	PTET.51.1.G0010374	0.1245	1.0335	8.3021
PGML3b	PTET.51.1.G0080308	0.0404	1.1559	28.6183
PGML3c	PTET.51.1.G0020217	0.1508	1.0885	7.216
PGML4a	PTET.51.1.G0340197	0.1161	0.9593	8.2612
PGML4b	PTET.51.1.G0480099	0.2535	1.1062	4.3641
PGML5a	PTET.51.1.G0570051	0.0141	1.1514	81.7442
PGML5b	PTET.51.1.G0510172	0.0138	1.1642	84.3893

Table S6. Substitution rates between *Paramecium tetraurelia* and *Paramecium octaurelia* PiggyMac and PiggyMac-likes.

**Gene abbreviation**	***P. tetraurelia* gene ID**	***P. octaurelia* gene ID**	**d_N/d_S**	**d_N**	**d_S**
PGM	PTET.51.1.G0490162	POCT.K8.1.G718000027 70580243	0.0234	0.0073	0.3106
PGML2	PTET.51.1.G0380073	POCT.K8.1.G718000027 70130227	0.0180	0.0045	0.2507
PGML3a	PTET.51.1.G0010374	POCT.K8.1.G718000027 70510320	0.0229	0.0082	0.3600
PGML3b	PTET.51.1.G0080308	POCT.K8.1.G718000027 70810134	0.0818	0.0245	0.2993
PGML3c	PTET.51.1.G0020217	POCT.K8.1.G718000027 70610330	0.1052	0.0365	0.3469
PGML4a	PTET.51.1.G0340197	POCT.K8.1.G718000027 70180100	0.0425	0.0139	0.3262
PGML4b	PTET.51.1.G0480099	POCT.K8.1.G718000027 70140101	0.0627	0.0153	0.2445
PGML5a	PTET.51.1.G0570051	POCT.K8.1.G718000027 70010048	0.0393	0.0110	0.2800
PGML5b	PTET.51.1.G0510172	POCT.K8.1.G718000027 69800173	0.0596	0.0123	0.2071

Observed d_N/d_S values for orthologous pairs of PiggyMac and PiggyMac-like proteins from *P. tetraurelia* and *P. octaurelia*.

Table S7. *Blepharisma* PiggyMac-like substitution rates (Reference gene: Contig_49.g1063).

Gene ID	ENA accession	d_N/d_S	d_N	d_S
Contig_3.g998	BSTOLATCC_MAC9455	0.0093	0.7106	76.4367
Contig_13.g879	BSTOLATCC_MAC2191	0.0551	0.8871	16.0867
Contig_13.g927	BSTOLATCC_MAC2239	0.0261	0.5547	21.2267
Contig_17.g391	BSTOLATCC_MAC3091	0.0087	0.8394	96.9223
Contig_17.g392	BSTOLATCC_MAC3092	0.0076	0.8195	107.6866
Contig_60.g827	BSTOLATCC_MAC23745	0.1351	0.8401	6.2209
Contig_61.g932	BSTOLATCC_MAC23855	0.0836	0.7727	9.2391
cORF_Contig_17. g3	BSTOLATCC_MIC7875	0.0765	0.653	8.5395
cORF_Contig_17. g4/5	BSTOLATCC_MIC7876 / BSTOLATCC_MIC7877	0.0068	0.5852	85.9998
cORF_Contig_21. g21	BSTOLATCC_MIC14766	0.0697	0.4729	6.7874
cORF_Contig_39. g3	BSTOLATCC_MIC33289	0.2763	1.2445	4.5036
cORF_Contig_39. g3/4	BSTOLATCC_MIC33289 / BSTOLATCC_MIC33290	0.007	0.6817	97.5885

Table S8. Comparison of *Blepharisma stoltei* ATCC 30299 intron prediction performance.

	**AUGUSTUS***	**Intronarrator****
**True positives (TP) (real introns)**	45	61
**False positives (FP) (fake introns)**	62	0
**False negatives (FN) (missed introns)**	15	2
**Sensitivity: TP/(TP+FN)**	0.75	0.97
**Precision: TP/(TP+FP)**	0.42	1.00

* Parameters/source code adjusted as for *Stentor* (66).
** AUGUSTUS changes/parameters as in Ref. 66.

Table S9. Noncanonical introns (15 or 16 bp) in *Blepharisma stoltei* ATCC 30299.

Intron with 3 bp exon flanks	Length (bp)	Intronarrator/ AUGUSTUS gene ID	ENA gene accession	Spliced fraction
AAGgcaaattttatttagATT	15	Contig_10.g615	BSTOLATCC_MAC443	0.827
AAGgcaactataatttagAGC	15	Contig_11.g1292	BSTOLATCC_MAC1123	0.73
CGAtatgagtttacaaatTTA	15	Contig_36.g880	BSTOLATCC_MAC12062	0.623
AAGgcaaaatttaaatagAGC	15	Contig_43.g513	BSTOLATCC_MAC15266	0.205
CAGgcaatttttatttagAAG	15	Contig_46.g452	BSTOLATCC_MAC16844	0.335
ATGgcaagctctatatagAAT	15	Contig_49.g1050	BSTOLATCC_MAC17453	0.797
TTActtctataaatacacCAA	15	Contig_54.g273	BSTOLATCC_MAC19826	0.537
AAGgcaaaaaatatatagGTT	15	Contig_58.g1437	BSTOLATCC_MAC22241	0.843
GAGgcaatttttacgtagATT	15	Contig_59.g298	BSTOLATCC_MAC22578	0.691
TGAggtaaattataactagGGT	16	Contig_2.g441	BSTOLATCC_MAC4446	0.54
AAGgtaatttcccagcaggAAT	16	Contig_3.g1280	BSTOLATCC_MAC9737	0.441
CCCttgctcccctcagtagTTA	16	Contig_6.g757	BSTOLATCC_MAC22759	0.477
ATGgtaactcacaattaggCTT	16	Contig_7.g1329	BSTOLATCC_MAC24915	0.283
CACgtaaaatacaattaggAGT	16	Contig_12.g347	BSTOLATCC_MAC1650	0.419
TATggtaatttgttatcagGGA	16	Contig_13.g1129	BSTOLATCC_MAC2441	0.326
ATGtaatttaccaatagggCTA	16	Contig_19.g1089	BSTOLATCC_MAC3792	0.867
ACAgtaagatttaattaggCCT	16	Contig_19.g1253	BSTOLATCC_MAC3956	0.525
TGAgtaagatacaagtaggAGG	16	Contig_21.g736	BSTOLATCC_MAC5692	0.644
AGGtaattggcaaatagggATA	16	Contig_24.g464	BSTOLATCC_MAC7001	0.415
AAGgtaaattacaagcaggAAA	16	Contig_25.g797	BSTOLATCC_MAC7334	0.764
CAAgtaattttcgaataggAAC	16	Contig_38.g1424	BSTOLATCC_MAC12612	0.78
AAGgtaatctctattaaggACA	16	Contig_42.g2	BSTOLATCC_MAC14754	0.602
AAGgcaattctctaggtagGAG	16	Contig_46.g332	BSTOLATCC_MAC16724	0.286
AGAggtaatgcataactagGGT	16	Contig_47.g486	BSTOLATCC_MAC16883	0.648
CCAgtaagtttctatttatGTC	16	Contig_55.g452	BSTOLATCC_MAC20010	0.715
AACgtaatttgtaactaggGGT	16	Contig_57.g559	BSTOLATCC_MAC21349	0.7
AAAgtaagagaccattaggTTA	16	Contig_57.g761	BSTOLATCC_MAC21551	0.726
ATTggtataggataattagGAA	16	Contig_60.g487	BSTOLATCC_MAC23405	0.376
TATacatgttttaaataatTGC	16	Contig_61.g1057	BSTOLATCC_MAC23980	0.278
AGAgtattttacaaataggCTA	16	Contig_63.g352	BSTOLATCC_MAC24681	0.585

Predicted introns are in lower case; flanking exons are in upper case. Different possible donor and acceptor site pairs of bases are colored. “Spliced fraction” indicates the efficiency of splicing calculated from Intronarrator.

Table S10. Tree topology tests with ciliate PiggyBac homologs.

Tree	logL	deltaL	bp-RELL	p-KH	p-SH	c-ELW	p-AU
**Unconstrained**	-97,183.2	0	0.536 +	0.691 +	1 +	0.535 +	0.688 +
**Monophyly of ALL ciliate PiggyBacs**	-97,536.0	352.77	0	0	0	-2.98E-82	6.38E-59 -
**Monophyly of ALL ciliate PiggyBacs + 4 non-ciliate interlopers**	-97,543.7	360.49	0	0	0	-3.73E-101	7.36E-59 -
**Monophyly of ciliate PiggyBacs - Tbp7 + 4 non-ciliate interlopers**	-97,199.2	15.98	0.211 +	0.309 +	0.68 +	0.212 +	0.347 +
**Monophyly of ciliate PiggyBacs - Tbp7 - 4 non-ciliate interlopers**	-97,200.0	16.79	0.253 +	0.324 +	0.68 +	0.253 +	0.363 +

"Non-ciliate interlopers": PBLEs PiggyBac-2 and PiggyBac-5 from *Chondrus crispus,* PiggyBac-1 from *Paracoccidoides brasiliensis* and PiggyBac-1 from *Mucor circinelloides*

deltaL : logL difference from the maximal log likelihood in the set.
bp-RELL : bootstrap proportion using RELL method (Kishino et al. 1990).
p-KH : p-value of one-sided Kishino-Hasegawa test (1989).
p-SH : p-value of Shimodaira-Hasegawa test (2000).
c-ELW : Expected Likelihood Weight (Strimmer & Rambaut 2002).
p-AU : p-value of approximately unbiased (AU) test (Shimodaira, 2002).

Plus signs following numbers denote the 95% confidence sets.
Minus signs following numbers denote significant exclusion.
All tests performed 10,000 resamplings using the RELL method.

SI References

1. A. J. Repak, Encystment and excystment of the heterotrichous ciliate *Blepharisma stoltei* Isquith. *Journal of Protozoology* **5**, 407–412 (1968).
2. T. Harumoto, *et al.*, Chemical defense by means of pigmented extrusomes in the ciliate *Blepharisma japonicum*. *Eur. J. Protistol.* **34**, 458–470 (1998).
3. A. Miyake, J. Beyer, Cell interaction by means of soluble factors (gamones) in conjugation of *Blepharisma intermedium*. *Exp. Cell Res.* **76**, 15–24 (1973).
4. R. A. Andersen, *Algal Culturing Techniques*, 1st Edition (2004).
5. A. Miyake, T. Harumoto, B. Salvi, V. Rivola, Defensive function of pigment granules in *Blepharisma japonicum*. *Eur. J. Protistol.* **25**, 310–315 (1990).
6. M. R. Lauth, B. B. Spear, J. Heumann, D. M. Prescott, DNA of ciliated protozoa: DNA sequence diminution during macronuclear development of *Oxytricha*. *Cell* **7**, 67–74 (1976).
7. R. F. Kimball, The nature and inheritance of mating types in *Euplotes patella*. *Genetics* **27**, 269–285 (1942).
8. R. Y. O. Katashima, Mating Types in *Euplotes eurystomus*. *J. Protozool.* **6**, 75–83 (1959).
9. P. Luporini, C. Miceli, C. Ortenzi, Evidence that the ciliate *Euplotes raikovi* releases mating-inducing factors (gamones). *J. Exp. Zool.* **226**, 1–9 (1983).
10. A. Vallesi, G. Giuli, R. A. Bradshaw, P. Luporini, Autocrine mitogenic activity of pheromones produced by the protozoan ciliate *Euplotes raikovi*. *Nature* **376**, 522–524 (1995).
11. A. Miyake, J. Beyer, Blepharmone: a conjugation-inducing glycoprotein in the ciliate *Blepharisma*. *Science* **185**, 621–623 (1974).
12. M. Sugiura, T. Harumoto, Identification, characterization, and complete amino acid sequence of the conjugation-inducing glycoprotein (blepharmone) in the ciliate *Blepharisma japonicum*. *Proc Natl Acad Sci USA* **98**, 14446–14451 (2001).
13. T. Kubota, T. Tokoroyama, Y. Tsukuda, H. Koyama, A. Miyake, Isolation and structure determination of blepharismin, a conjugation initiating gamone in the ciliate *Blepharisma*. *Science* **179**, 400–402 (1973).
14. A. Miyake, "Cell interaction by gamones in *Blepharisma*" in *Sexual Interactions in Eukaryotic Microbes*, (Elsevier, 1981), pp. 95–129.
15. A. Miyake, V. Rivola, T. Harumoto, Double paths of macronucleus differentiation at conjugation in *Blepharisma japonicum*. *Eur. J. Protistol.* **27**, 178–200 (1991).
16. M. Sugiura, Y. Tanaka, T. Suzaki, T. Harumoto, Alternative gene expression in type **I** and type **II** cells may enable further nuclear changes during conjugation of *Blepharisma japonicum*. *Protist* **163**, 204–216 (2012).
17. B. K. B. Seah, C. Emmerich, A. Singh, E. C. Swart, Improved methods for bulk cultivation and fixation of *Loxodes* ciliates for fluorescence microscopy. *Protist* **173**, 125905 (2022).
18. J. Schindelin, *et al.*, Fiji: an open-source platform for biological-image analysis. *Nat. Methods* **9**, 676–682 (2012).
19. M. Kolmogorov, J. Yuan, Y. Lin, P. A. Pevzner, Assembly of long, error-prone reads using repeat graphs. *Nat. Biotechnol.* **37**, 540–546 (2019).
20. H. Li, Minimap2: pairwise alignment for nucleotide sequences. *Bioinformatics* **34**, 3094–3100 (2018).
21. M. Kearse, *et al.*, Geneious Basic: an integrated and extendable desktop software platform for the organization and analysis of sequence data. *Bioinformatics* **28**, 1647–1649 (2012).
22. A. Prjibelski, D. Antipov, D. Meleshko, A. Lapidus, A. Korobeynikov, Using SPAdes de novo assembler. *Curr. Protoc. Bioinformatics* **70**, e102 (2020).
23. R. Vaser, M. Sikic, Yet another de novo genome assembler in *2019 11th International Symposium on Image and Signal Processing and Analysis (ISPA)*, (IEEE, 2019), pp. 147–151.
24. E. C. Swart, V. Serra, G. Petroni, M. Nowacki, Genetic Codes with No Dedicated Stop Codon: Context-Dependent Translation Termination. *Cell* **166**, 691–702 (2016).
25. H. R. Gruber-Vodicka, B. K. B. Seah, E. Pruesse, phyloFlash: Rapid Small-Subunit rRNA Profiling and Targeted Assembly from Metagenomes. *mSystems* **5** (2020).

26. C. Quast, *et al.*, The SILVA ribosomal RNA gene database project: improved data processing and web-based tools. *Nucleic Acids Res.* **41**, D590-6 (2013).
27. D. Hyatt, *et al.*, Prodigal: prokaryotic gene recognition and translation initiation site identification. *BMC Bioinformatics* **11**, 119 (2010).
28. A. Gurevich, V. Saveliev, N. Vyahhi, G. Tesler, QUAST: quality assessment tool for genome assemblies. *Bioinformatics* **29**, 1072–1075 (2013).
29. B. Langmead, S. L. Salzberg, Fast gapped-read alignment with Bowtie 2. *Nat. Methods* **9**, 357–359 (2012).
30. P. Danecek, *et al.*, Twelve years of SAMtools and BCFtools. *Gigascience* **10** (2021).
31. E. Garrison, G. Marth, “Haplotype-based variant detection from short-read sequencing,” v2 Ed. (arXiv, 2012).
32. Y. Sheng, *et al.*, The completed macronuclear genome of a model ciliate *Tetrahymena thermophila* and its application in genome scrambling and copy number analyses. *Sci. China Life Sci.* **63**, 1534–1542 (2020).
33. R. S. Harris, M. Cechova, K. D. Makova, Noise-cancelling repeat finder: uncovering tandem repeats in error-prone long-read sequencing data. *Bioinformatics* **35**, 4809–4811 (2019).
34. B. K. B. Seah, E. C. Swart, BleTIES: Annotation of natural genome editing in ciliates using long read sequencing. *Bioinformatics* **37**, 3929–3931 (2021).
35. P. J. A. Cock, *et al.*, Biopython: freely available Python tools for computational molecular biology and bioinformatics. *Bioinformatics* **25**, 1422–1423 (2009).
36. R. K. Dale, B. S. Pedersen, A. R. Quinlan, Pybedtools: a flexible Python library for manipulating genomic datasets and annotations. *Bioinformatics* **27**, 3423–3424 (2011).
37. A. R. Quinlan, I. M. Hall, BEDTools: a flexible suite of utilities for comparing genomic features. *Bioinformatics* **26**, 841–842 (2010).
38. J. D. Hunter, Matplotlib: A 2D Graphics Environment. *Comput. Sci. Eng.* **9**, 90–95 (2007).
39. G. E. Crooks, G. Hon, J. M. Chandonia, S. E. Brenner, WebLogo: a sequence logo generator. *Genome Res.* **14**, 1188–1190 (2004).
40. D. Kim, J. M. Paggi, C. Park, C. Bennett, S. L. Salzberg, Graph-based genome alignment and genotyping with HISAT2 and HISAT-genotype. *Nat. Biotechnol.* **37**, 907–915 (2019).
41. B. E. Dutilh, *et al.*, FACIL: Fast and Accurate Genetic Code Inference and Logo. *Bioinformatics* **27**, 1929–1933 (2011).
42. S. R. Eddy, Accelerated profile HMM searches. *PLoS Comput. Biol.* **7**, e1002195 (2011).
43. B. Saudemont, *et al.*, The fitness cost of mis-splicing is the main determinant of alternative splicing patterns. *Genome Biol.* **18**, 208 (2017).
44. E. P. Nawrocki, D. L. Kolbe, S. R. Eddy, Infernal 1.0: inference of RNA alignments. *Bioinformatics* **25**, 1335–1337 (2009).
45. I. Kalvari, *et al.*, Rfam 13.0: shifting to a genome-centric resource for non-coding RNA families. *Nucleic Acids Res.* **46**, D335–D342 (2018).
46. P. P. Chan, B. Y. Lin, A. J. Mak, T. M. Lowe, tRNAscan-SE 2.0: Improved Detection and Functional Classification of Transfer RNA Genes. *BioRxiv* (2019) https:/doi.org/10.1101/614032.
47. L. Lopez-Delisle, *et al.*, pyGenomeTracks: reproducible plots for multivariate genomic datasets. *Bioinformatics* **37**, 422–423 (2021).
48. R. M. Waterhouse, *et al.*, BUSCO applications from quality assessments to gene prediction and phylogenomics. *Mol. Biol. Evol.* **35**, 543–548 (2018).
49. P. Törönen, A. Medlar, L. Holm, PANNZER2: a rapid functional annotation web server. *Nucleic Acids Res.* **46**, W84–W88 (2018).
50. J. Huerta-Cepas, *et al.*, eggNOG 5.0: a hierarchical, functionally and phylogenetically annotated orthology resource based on 5090 organisms and 2502 viruses. *Nucleic Acids Res.* **47**, D309–D314 (2019).
51. Y. Liao, G. K. Smyth, W. Shi, featureCounts: an efficient general purpose program for assigning sequence reads to genomic features. *Bioinformatics* **30**, 923–930 (2014).
52. M. D. Robinson, D. J. McCarthy, G. K. Smyth, edgeR: a Bioconductor package for differential expression analysis of digital gene expression data. *Bioinformatics* **26**, 139–140

(2010).
53. B. Li, V. Ruotti, R. M. Stewart, J. A. Thomson, C. N. Dewey, RNA-Seq gene expression estimation with read mapping uncertainty. *Bioinformatics* **26**, 493–500 (2010).
54. G. P. Wagner, K. Kin, V. J. Lynch, Measurement of mRNA abundance using RNA-seq data: RPKM measure is inconsistent among samples. *Theory Biosci.* **131**, 281–285 (2012).
55. K. Katoh, K. Misawa, K. Kuma, T. Miyata, MAFFT: a novel method for rapid multiple sequence alignment based on fast Fourier transform. *Nucleic Acids Res.* **30**, 3059–3066 (2002).
56. K. Katoh, D. M. Standley, MAFFT multiple sequence alignment software version 7: improvements in performance and usability. *Mol. Biol. Evol.* **30**, 772–780 (2013).
57. S. Guindon, *et al.*, New algorithms and methods to estimate maximum-likelihood phylogenies: assessing the performance of PhyML 3.0. *Syst. Biol.* **59**, 307–321 (2010).
58. N. A. Stover, R. S. Punia, M. S. Bowen, S. B. Dolins, T. G. Clark, *Tetrahymena* Genome Database Wiki: a community-maintained model organism database. *Database (Oxford)* **2012**, bas007 (2012).
59. R.-L. Wang, W. Miao, W. Wang, J. Xiong, A.-H. Liang, EOGD: the *Euplotes octocarinatus* genome database. *BMC Genomics* **19**, 63 (2018).
60. O. Arnaiz, E. Meyer, L. Sperling, ParameciumDB 2019: integrating genomic data across the genus for functional and evolutionary biology. *Nucleic Acids Res.* **48**, D599–D605 (2020).
61. J. Mistry, *et al.*, Pfam: The protein families database in 2021. *Nucleic Acids Res.* **49**, D412–D419 (2021).
62. P. Jones, *et al.*, InterProScan 5: genome-scale protein function classification. *Bioinformatics* **30**, 1236–1240 (2014).
63. E. Quevillon, *et al.*, InterProScan: protein domains identifier. *Nucleic Acids Res.* **33**, W116-20 (2005).
64. Z. Yang, PAML 4: phylogenetic analysis by maximum likelihood. *Mol. Biol. Evol.* **24**, 1586–1591 (2007).
65. M. Bouallègue, J.-D. Rouault, A. Hua-Van, M. Makni, P. Capy, Molecular evolution of piggyBac superfamily: From selfishness to domestication. *Genome Biol. Evol.* **9**, 323–339 (2017).
66. M. M. Slabodnick, *et al.*, The macronuclear genome of *Stentor coeruleus* reveals tiny introns in a giant cell. *Curr. Biol.* **27**, 569–575 (2017).
67. E. C. Swart, *et al.*, The *Oxytricha trifallax* macronuclear genome: a complex eukaryotic genome with 16,000 tiny chromosomes. *PLoS Biol.* **11**, e1001473 (2013).
68. M. C. Yao, C. H. Yao, B. Monks, The controlling sequence for site-specific chromosome breakage in *Tetrahymena*. *Cell* **63**, 763–772 (1990).
69. A. R. O. Cavalcanti, *et al.*, Sequence features of *Oxytricha trifallax* (class Spirotrichea) macronuclear telomeric and subtelomeric sequences. *Protist* **155**, 311–322 (2004).
70. C. D. Putnam, V. Pennaneach, R. D. Kolodner, Chromosome healing through terminal deletions generated by de novo telomere additions in *Saccharomyces cerevisiae*. *Proc Natl Acad Sci USA* **101**, 13262–13267 (2004).
71. G. B. Morin, Recognition of a chromosome truncation site associated with alpha-thalassaemia by human telomerase. *Nature* **353**, 454–456 (1991).
72. H. Wang, E. H. Blackburn, De novo telomere addition by *Tetrahymena* telomerase in vitro. *EMBO J.* **16**, 866–879 (1997).
73. J. T. Gray, D. W. Celander, C. M. Price, T. R. Cech, Cloning and expression of genes for the *Oxytricha* telomere-binding protein: specific subunit interactions in the telomeric complex. *Cell* **67**, 807–814 (1991).
74. S. Cranert, S. Heyse, B. R. Linger, R. Lescasse, C. Price, *Tetrahymena* Pot2 is a developmentally regulated paralog of Pot1 that localizes to chromosome breakage sites but not to telomeres. *Eukaryotic Cell* **13**, 1519–1529 (2014).
75. M. Lynch, The origins of eukaryotic gene structure. *Mol. Biol. Evol.* **23**, 450–468 (2006).
76. S. W. Roy, A. J. Hudson, J. Joseph, J. Yee, A. G. Russell, Numerous fragmented spliceosomal introns, AT-AC splicing, and an unusual dynein gene expression pathway in

Giardia lamblia. *Mol. Biol. Evol.* **29**, 43–49 (2012).
77. V. S. Bondarenko, M. S. Gelfand, Evolution of the Exon-Intron Structure in Ciliate Genomes. *PLoS ONE* **11**, e0161476 (2016).
78. M. Csuros, I. B. Rogozin, E. V. Koonin, A detailed history of intron-rich eukaryotic ancestors inferred from a global survey of 100 complete genomes. *PLoS Comput. Biol.* **7**, e1002150 (2011).
79. A. G. Russell, J. M. Charette, D. F. Spencer, M. W. Gray, An early evolutionary origin for the minor spliceosome. *Nature* **443**, 863–866 (2006).
80. N. Sheth, *et al.*, Comprehensive splice-site analysis using comparative genomics. *Nucleic Acids Res.* **34**, 3955–3967 (2006).
81. M. Stanke, S. Waack, Gene prediction with a hidden Markov model and a new intron submodel. *Bioinformatics* **19 Suppl 2**, ii215-25 (2003).
82. A. Krogh, B. Larsson, G. von Heijne, E. L. L. Sonnhammer, Predicting transmembrane protein topology with a hidden Markov model: application to complete genomes. *J. Mol. Biol.* **305**, 567–580 (2001).
83. J.-M. Aury, *et al.*, Global trends of whole-genome duplications revealed by the ciliate *Paramecium tetraurelia*. *Nature* **444**, 171–178 (2006).
84. J. A. Eisen, *et al.*, Macronuclear genome sequence of the ciliate *Tetrahymena thermophila*, a model eukaryote. *PLoS Biol.* **4**, e286 (2006).
85. J. A. Bailey, A. M. Yavor, H. F. Massa, B. J. Trask, E. E. Eichler, Segmental duplications: organization and impact within the current human genome project assembly. *Genome Res.* **11**, 1005–1017 (2001).
86. D. M. Emms, S. Kelly, OrthoFinder: solving fundamental biases in whole genome comparisons dramatically improves orthogroup inference accuracy. *Genome Biol.* **16**, 157 (2015).
87. C. Lenain, *et al.*, The Apollo 5’ exonuclease functions together with TRF2 to protect telomeres from DNA repair. *Curr. Biol.* **16**, 1303–1310 (2006).
88. G. Mondal, M. Stevers, B. Goode, A. Ashworth, D. A. Solomon, A requirement for STAG2 in replication fork progression creates a targetable synthetic lethality in cohesin-mutant cancers. *Nat. Commun.* **10**, 1686 (2019).
89. A. Rojowska, *et al.*, Structure of the Rad50 DNA double-strand break repair protein in complex with DNA. *EMBO J.* **33**, 2847–2859 (2014).
90. A. Lukaszewicz, R. A. Howard-Till, J. Loidl, Mus81 nuclease and Sgs1 helicase are essential for meiotic recombination in a protist lacking a synaptonemal complex. *Nucleic Acids Res.* **41**, 9296–9309 (2013).
91. A. A. Riccio, G. Cingolani, J. M. Pascal, PARP-2 domain requirements for DNA damage-dependent activation and localization to sites of DNA damage. *Nucleic Acids Res.* **44**, 1691–1702 (2016).
92. S. Travali, *et al.*, Structure of the human gene for the proliferating cell nuclear antigen. *J. Biol. Chem.* **264**, 7466–7472 (1989).
93. K. K. Shivji, M. K. Kenny, R. D. Wood, Proliferating cell nuclear antigen is required for DNA excision repair. *Cell* **69**, 367–374 (1992).
94. C. D. Mol, C. F. Kuo, M. M. Thayer, R. P. Cunningham, J. A. Tainer, Structure and function of the multifunctional DNA-repair enzyme exonuclease III. *Nature* **374**, 381–386 (1995).
95. D. F. Corona, *et al.*, ISWI is an ATP-dependent nucleosome remodeling factor. *Mol. Cell* **3**, 239–245 (1999).
96. A. Singh, *et al.*, Chromatin remodeling is required for sRNA-guided DNA elimination in *Paramecium*. *EMBO J.*, in press.
97. P.-H. Chung, M.-C. Yao, *Tetrahymena thermophila* JMJD3 homolog regulates H3K27 methylation and nuclear differentiation. *Eukaryotic Cell* **11**, 601–614 (2012).
98. N. Sonenberg, A. G. Hinnebusch, Regulation of translation initiation in eukaryotes: mechanisms and biological targets. *Cell* **136**, 731–745 (2009).
99. H. Wang, E. C. Curran, T. R. Hinds, E. H. Wang, N. Zheng, Crystal structure of a TAF1-TAF7 complex in human transcription factor IID reveals a promoter binding module. *Cell Res.* **24**, 1433–1444 (2014).

100. B. D. Dynlacht, T. Hoey, R. Tjian, Isolation of coactivators associated with the TATA-binding protein that mediate transcriptional activation. *Cell* **66**, 563–576 (1991).
101. J. Gruchota, C. Denby Wilkes, O. Arnaiz, L. Sperling, J. K. Nowak, A meiosis-specific Spt5 homolog involved in non-coding transcription. *Nucleic Acids Res.* **45**, 4722–4732 (2017).
102. Z. T. Neeb, D. J. Hogan, S. Katzman, A. M. Zahler, Preferential expression of scores of functionally and evolutionarily diverse DNA and RNA-binding proteins during *Oxytricha trifallax* macronuclear development. *PLoS ONE* **12**, e0170870 (2017).
103. M. Guérineau, *et al.*, The unusual structure of the PiggyMac cysteine-rich domain reveals zinc finger diversity in PiggyBac-related transposases. *Mob. DNA* **12**, 12 (2021).
104. B. Gao, *et al.*, Evolution of pogo, a separate superfamily of IS630-Tc1-mariner transposons, revealing recurrent domestication events in vertebrates. *Mob. DNA* **11**, 25 (2020).
105. D. Mojzita, S. Hohmann, Pdc2 coordinates expression of the THI regulon in the yeast Saccharomyces cerevisiae. *Mol. Genet. Genomics* **276**, 147–161 (2006).
106. S. Hohmann, Characterisation of PDC2, a gene necessary for high level expression of pyruvate decarboxylase structural genes in *Saccharomyces cerevisiae*. *Mol. Gen. Genet.* **241**, 657–666 (1993).
107. C. Casola, D. Hucks, C. Feschotte, Convergent domestication of pogo-like transposases into centromere-binding proteins in fission yeast and mammals. *Mol. Biol. Evol.* **25**, 29–41 (2008).
108. D. J. Witherspoon, *et al.*, Selection on the protein-coding genes of the TBE1 family of transposable elements in the ciliates *Oxytricha fallax* and *O. trifallax*. *Mol. Biol. Evol.* **14**, 696–706 (1997).
109. K. Williams, T. G. Doak, G. Herrick, Developmental precise excision of *Oxytricha trifallax* telomere-bearing elements and formation of circles closed by a copy of the flanking target duplication. *EMBO J.* **12**, 4593–4601 (1993).
110. M. Nowacki, *et al.*, A functional role for transposases in a large eukaryotic genome. *Science* **324**, 935–938 (2009).
111. C. L. Jahn, S. Z. Doktor, J. S. Frels, J. W. Jaraczewski, M. F. Krikau, Structures of the *Euplotes crassus* Tec1 and Tec2 elements: identification of putative transposase coding regions. *Gene* **133**, 71–78 (1993).
112. T. G. Doak, F. P. Doerder, C. L. Jahn, G. Herrick, A proposed superfamily of transposase genes: transposon-like elements in ciliated protozoa and a common "D35E" motif. *Proc Natl Acad Sci USA* **91**, 942–946 (1994).
113. M. Dupeyron, T. Baril, C. Bass, A. Hayward, Phylogenetic analysis of the Tc1/mariner superfamily reveals the unexplored diversity of pogo-like elements. *Mob. DNA* **11**, 21 (2020).
114. O. Arnaiz, *et al.*, The *Paramecium* germline genome provides a niche for intragenic parasitic DNA: evolutionary dynamics of internal eliminated sequences. *PLoS Genet.* **8**, e1002984 (2012).
115. S. H. Aeschlimann, *et al.*, The draft assembly of the radically organized *Stylonychia lemnae* macronuclear genome. *Genome Biol. Evol.* **6**, 1707–1723 (2014).
116. Y.-W. Yuan, S. R. Wessler, The catalytic domain of all eukaryotic cut-and-paste transposase superfamilies. *Proc Natl Acad Sci USA* **108**, 7884–7889 (2011).
117. X. Chen, *et al.*, The architecture of a scrambled genome reveals massive levels of genomic rearrangement during development. *Cell* **158**, 1187–1198 (2014).
118. D. L. Chalker, E. Meyer, K. Mochizuki, Epigenetics of ciliates. *Cold Spring Harb. Perspect. Biol.* **5**, a017764 (2013).
119. P. Y. Sandoval, E. C. Swart, M. Arambasic, M. Nowacki, Functional diversification of Dicer-like proteins and small RNAs required for genome sculpting. *Dev. Cell* **28**, 174–188 (2014).
120. K. Mochizuki, N. A. Fine, T. Fujisawa, M. A. Gorovsky, Analysis of a piwi-related gene implicates small RNAs in genome rearrangement in *tetrahymena*. *Cell* **110**, 689–699 (2002).

121. K. Mochizuki, M. A. Gorovsky, A Dicer-like protein in *Tetrahymena* has distinct functions in genome rearrangement, chromosome segregation, and meiotic prophase. *Genes Dev.* **19**, 77–89 (2005).
122. G. Lepère, *et al.*, Silencing-associated and meiosis-specific small RNA pathways in *Paramecium tetraurelia*. *Nucleic Acids Res.* **37**, 903–915 (2009).
123. U. E. Schoeberl, H. M. Kurth, T. Noto, K. Mochizuki, Biased transcription and selective degradation of small RNAs shape the pattern of DNA elimination in *Tetrahymena. Genes Dev.* **26**, 1729–1742 (2012).
124. T. Noto, K. Mochizuki, Small RNA-mediated trans-nuclear and trans-element communications in *Tetrahymena* DNA elimination. *Curr. Biol.* **28**, 1938-1949.e5 (2018).
125. S. A. Shabalina, E. V. Koonin, Origins and evolution of eukaryotic RNA interference. *Trends Ecol. Evol.* **23**, 578–587 (2008).
126. U. Götz, *et al.*, Two sets of RNAi components are required for heterochromatin formation in trans triggered by truncated transgenes. *Nucleic Acids Res.* **44**, 5908–5923 (2016).
127. K. Bouhouche, J.-F. Gout, A. Kapusta, M. Bétermier, E. Meyer, Functional specialization of Piwi proteins in *Paramecium tetraurelia* from post-transcriptional gene silencing to genome remodelling. *Nucleic Acids Res.* **39**, 4249–4264 (2011).
128. W. Fang, X. Wang, J. R. Bracht, M. Nowacki, L. F. Landweber, Piwi-interacting RNAs protect DNA against loss during *Oxytricha* genome rearrangement. *Cell* **151**, 1243–1255 (2012).
129. C. P. Fetzer, D. J. Hogan, H. J. Lipps, A PIWI homolog is one of the proteins expressed exclusively during macronuclear development in the ciliate *Stylonychia lemnae*. *Nucleic Acids Res.* **30**, 4380–4386 (2002).
130. G. E. Zentner, S. Henikoff, Regulation of nucleosome dynamics by histone modifications. *Nat. Struct. Mol. Biol.* **20**, 259–266 (2013).
131. S. Bilokapic, M. Strauss, M. Halic, Structural rearrangements of the histone octamer translocate DNA. *Nat. Commun.* **9**, 1330 (2018).
132. Y. Liu, *et al.*, RNAi-dependent H3K27 methylation is required for heterochromatin formation and DNA elimination in Tetrahymena. *Genes Dev.* **21**, 1530–1545 (2007).
133. D. L. Chalker, Dynamic nuclear reorganization during genome remodeling of *Tetrahymena*. *Biochim. Biophys. Acta* **1783**, 2130–2136 (2008).
134. A. Frapporti, *et al.*, The Polycomb protein Ezl1 mediates H3K9 and H3K27 methylation to repress transposable elements in Paramecium. *Nat. Commun.* **10**, 2710 (2019).
135. J. Postberg, *et al.*, 27nt-RNAs guide histone variant deposition via “RNA-induced DNA replication interference” and thus transmit parental genome partitioning in Stylonychia. *Epigenetics Chromatin* **11**, 31 (2018).
136. H. S. Malik, S. Henikoff, Phylogenomics of the nucleosome. *Nat. Struct. Biol.* **10**, 882–891 (2003).
137. M. D. Cervantes, X. Xi, D. Vermaak, M.-C. Yao, H. S. Malik, The CNA1 histone of the ciliate *Tetrahymena thermophila* is essential for chromosome segregation in the germline micronucleus. *Mol. Biol. Cell* **17**, 485–497 (2006).
138. C. A. Whittaker, R. O. Hynes, Distribution and evolution of von Willebrand/integrin A domains: widely dispersed domains with roles in cell adhesion and elsewhere. *Mol. Biol. Cell* **13**, 3369–3387 (2002).
139. S. Forcob, A. Bulic, F. Jönsson, H. J. Lipps, J. Postberg, Differential expression of histone H3 genes and selective association of the variant H3.7 with a specific sequence class in *Stylonychia* macronuclear development. *Epigenetics Chromatin* **7**, 4 (2014).
140. R. S. Coyne, *et al.*, Comparative genomics of the pathogenic ciliate *Ichthyophthirius multifiliis*, its free-living relatives and a host species provide insights into adoption of a parasitic lifestyle and prospects for disease control. *Genome Biol.* **12**, R100 (2011).
141. R. R. Wick, M. B. Schultz, J. Zobel, K. E. Holt, Bandage: interactive visualization of de novo genome assemblies. *Bioinformatics* **31**, 3350–3352 (2015).
142. E. V. Kriventseva, *et al.*, OrthoDB v10: sampling the diversity of animal, plant, fungal, protist, bacterial and viral genomes for evolutionary and functional annotations of orthologs. *Nucleic Acids Res.* **47**, D807–D811 (2019).
